# Supplementary material for: Single-cell biological network inference using a heterogeneous graph transformer
Source: Nat Commun. 2023 Feb 21;14:964. doi: 10.1038/s41467-023-36559-0 (PMC9944243; doi:10.1038/s41467-023-36559-0)
Supplement: Supplementary file 1 — Supplementary Information [file 41467_2023_36559_MOESM1_ESM.docx]

**Supplementary Information of**

**Single-cell biological network inference using a heterogeneous graph transformer**

Anjun Ma^1,2,^*, Xiaoying Wang^3,^*, Jingxian Li^3^, Cankun Wang^1^, Tong Xiao^2^, Yuntao Liu^3^, Hao Cheng^1^, Juexin Wang^4,5^, Yang Li^1^, Yuzhou Chang^1,2^, Jinpu Li^5,6^, Duolin Wang^4,5^, Yuexu Jiang^4,5^, Li Su^5,6^, Gang Xin^2^, Shaopeng Gu^1^, Zihai Li^2^, Bingqiang Liu^3,$^, Dong Xu^4,5,6, $^, Qin Ma^1,2,$^

^1^ Department of Biomedical Informatics, College of Medicine, The Ohio State University, Columbus, OH, 43210, USA

^2^ Pelotonia Institute for Immuno-Oncology, The James Comprehensive Cancer Center, The Ohio State University, Columbus, OH 43210, USA

^3^ School of Mathematics, Shandong University, Jinan, Shandong, 250100, China

^4^ Department of Electrical Engineering and Computer Science, University of Missouri, Columbia, MO 65211, USA

^5^ Christopher S. Bond Life Sciences Center, University of Missouri, Columbia, MO 65211, USA.

^6^ Institute for Data Science and Informatics, University of Missouri, Columbia, MO 65211, USA

^*^ These authors contributed equally

^$^ These authors jointly supervised this work: Qin Ma: [qin.ma@osumc.edu](mailto:qin.ma@osumc.edu); Dong Xu: [xudong@missouri.edu](mailto:xudong@missouri.edu); Bingqiang Liu: [bingqiang@sdu.edu.cn](mailto:bingqiang@sdu.edu.cn).


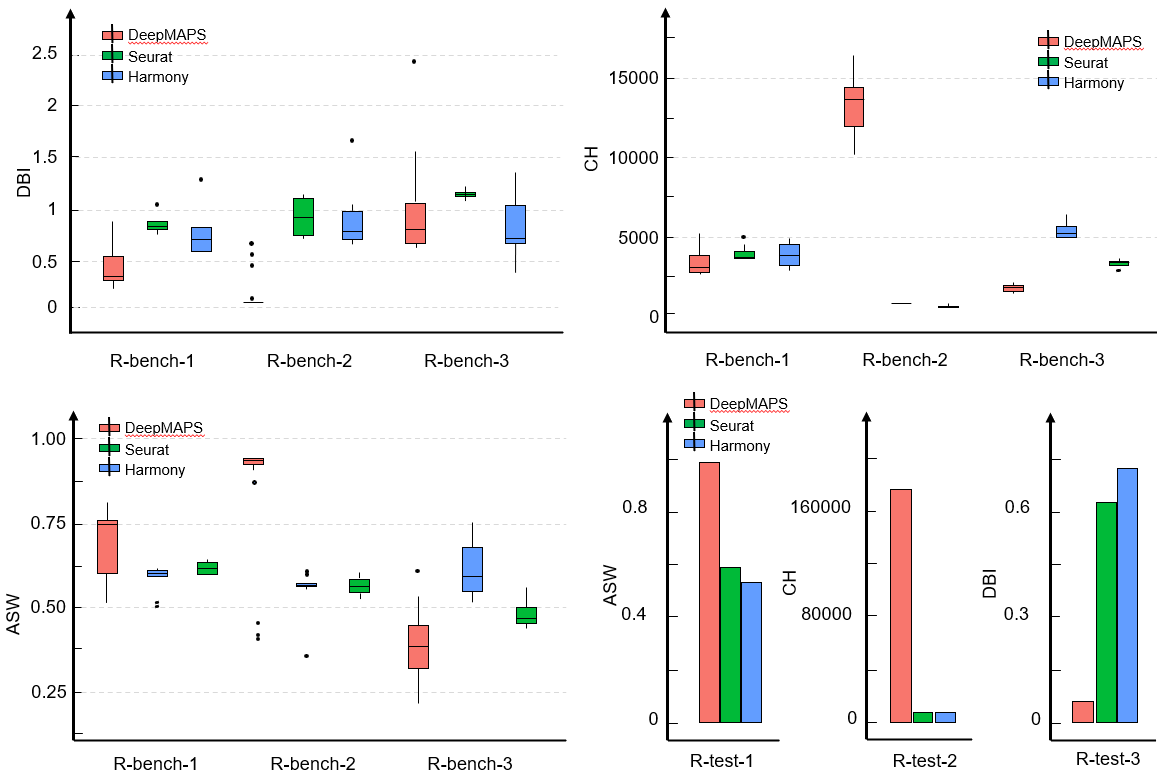


**Supplementary Fig. 1.** Benchmark cell clustering results of three multiple scRNA-seq data in terms of DBI, CH, and ASW scores. Each box showcases the minimum, first quartile, median, third quartile, and maximum DBI, CH, and ASW results of a tool using different parameter settings. Higher ASW and CH score indicate better tool performance; lower DBI score indicates better tool performance. DeepMAPS: n=96, Seurat: n=16, Harmony: n=36. Dots represent outliers. Source data are provided as a Source Data file.


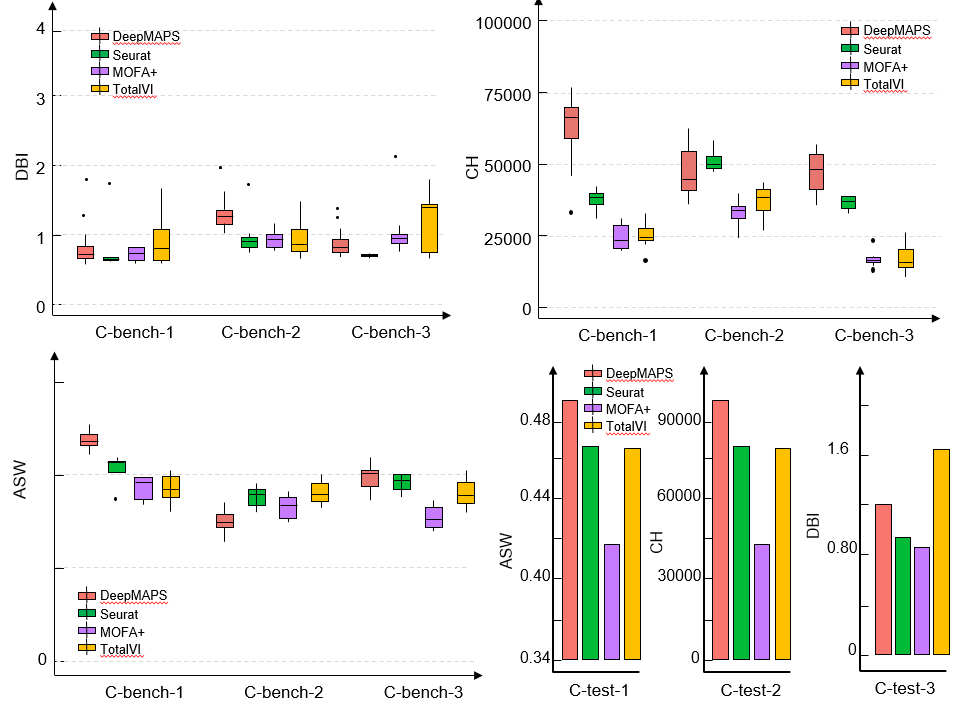


**Supplementary Fig. 2.** Benchmark cell clustering results of CITE-seq data in terms of DBI, CH, and ASW scores. Each box showcases the minimum, first quartile, median, third quartile, and maximum DBI, CH, and ASW results of a tool using different parameter settings. Higher ASW and CH score indicate better tool performance; lower DBI score indicates better tool performance. DeepMAPS: n=96, Seurat: n=16, MOFA+: n=36, TotalVI: n=48. Dots represent outliers. Source data are provided as a Source Data file.


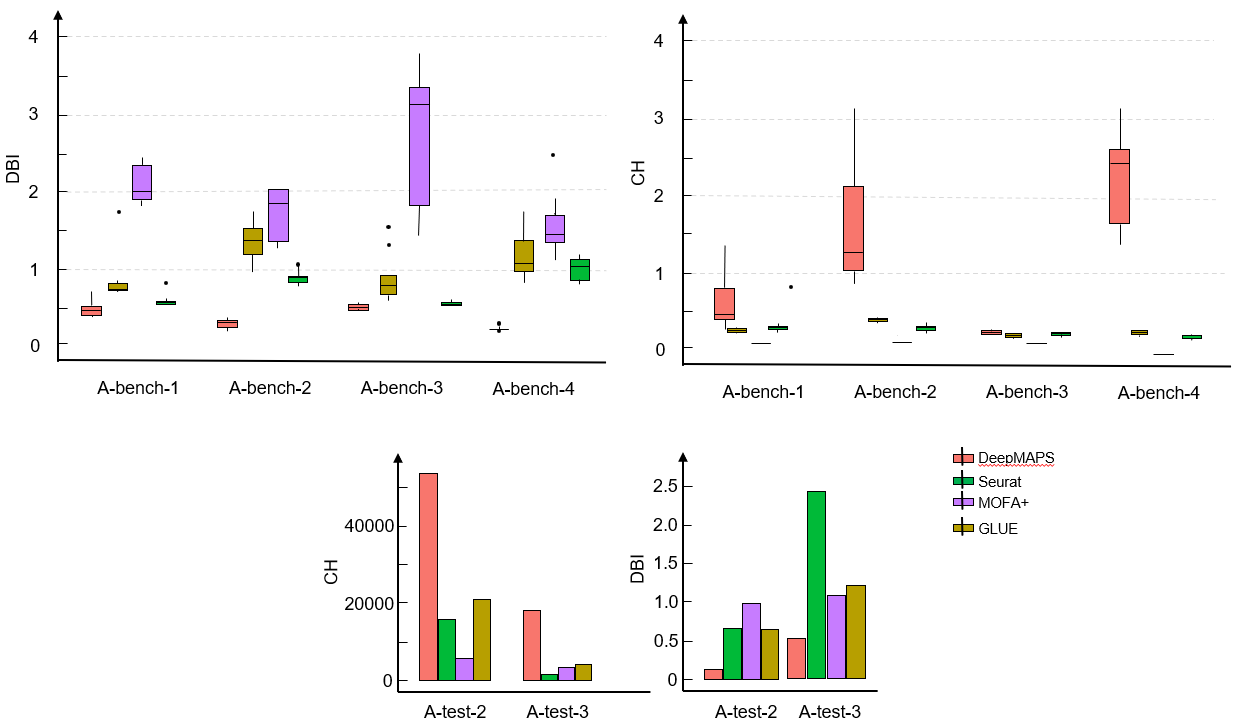


**Supplementary Fig. 3.** Benchmark cell clustering results of scRNA-seq and scATAC-seq integration in terms of DBI, CH, and ASW scores. Each box showcases the minimum, first quartile, median, third quartile, and maximum DBI, CH, and ASW results of a tool using different parameter settings. Higher ASW and CH score indicate better tool performance; lower DBI score indicates better tool performance. DeepMAPS: n=96, Seurat: n= 36, MOFA+: n=36, and GLUE: n=72. Dots represent outliers. Source data are provided as a Source Data file.

**Supplementary Fig. 4.** Evaluation of integration methods deployed in DeepMAPS. P-values were calculated by t-test. For each box, n=36 and dots represent outliers. Each box showcases the minimum, first quartile, median, third quartile, and maximum score of the corresponding criteria. The comparisons are performed by two-tail t-test. Source data are provided as a Source Data file.


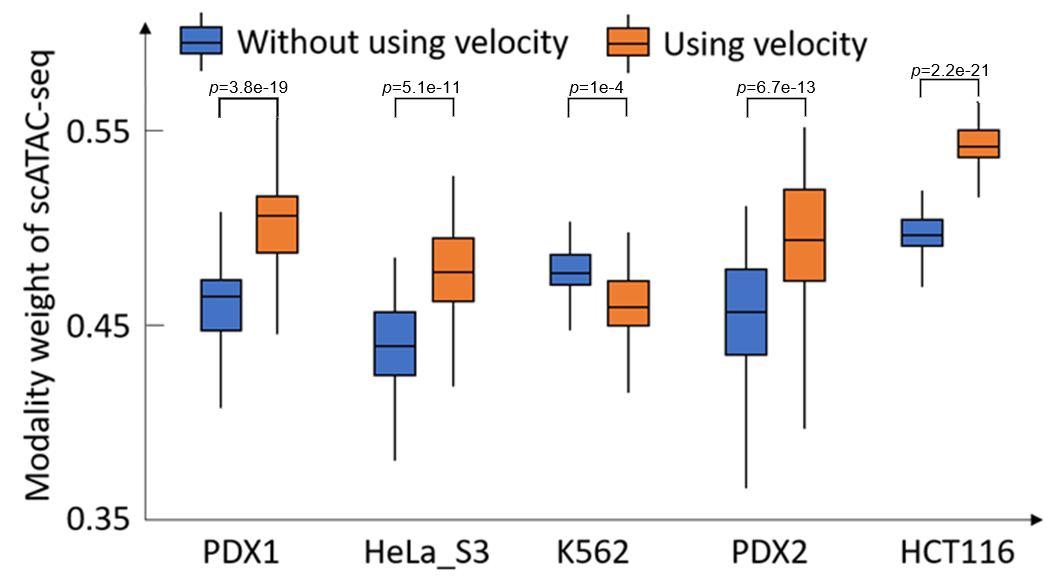


**Supplementary Fig. 5**. The comparison of modality weight of scATAC-seq in different cell clusters by using or without using the velocity-weighted balance method. The sum of the modality weight of chromatin accessibility (scATAC-seq) and gene expression (scRNA-seq) of a cell equals 1. Each box represents the distribution of chromatin accessibility weight of all cells in the corresponding cell cluster (A-test-1): n=176 (PDX1), 42 (HeLa_S3), 72 (K562), 161 (PDX2), and 63 (HCT116). Each box showcases the minimum, first quartile, median, third quartile, and maximum score of the corresponding criteria. The comparisons are performed by one-tail t-test. Source data are provided as a Source Data file.

**Supplementary Fig. 6.** Evaluation of cell clustering methods deployed in DeepMAPS. All comparisons between any of the two methods in the same dataset was performed by one-tail t-test, and none of the comparison is significant (p-value>0.1). For each box, n=36 and dots represent outliers. Each box showcases the minimum, first quartile, median, third quartile, and maximum score of the corresponding criteria. Source data are provided as a Source Data file.


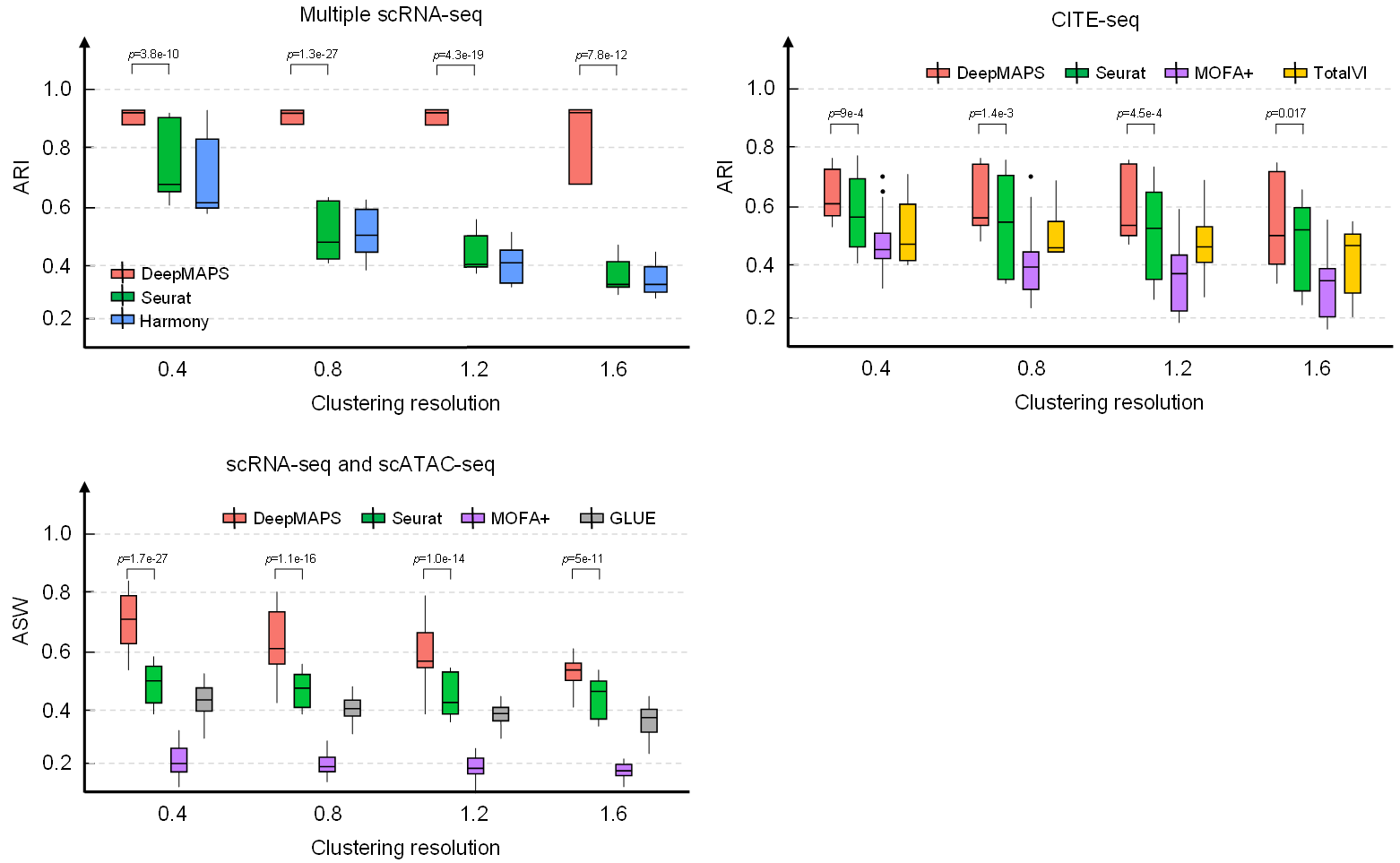


**Supplementary Fig. 7.** Grid-search test of cell clustering resolution between DeepMAPS and benchmark tools. Each box represents results of all parameter combinations on all benchmark datasets in the same data type. Multiple scRNA-seq DeepMAPS (n=108); Multiple scRNA-seq Seurat (n=30); Multiple scRNA-seq Harmony (n=27); CITE-seq DeepMAPS (n=108); CITE-seq Seurat (n=30); CITE-seq MOFA+ (n=27); CITE-seq TotalVI (n=36); scRNA-seq and scATAC-seq DeepMAPS (n=72); scRNA-seq and scATAC-seq Seurat (n=30); scRNA-seq and scATAC-seq MOFA+ (n=27); scRNA-seq and scATAC-seq GLUE (n=54). and Each box showcases the minimum, first quartile, median, third quartile, and maximum score of the corresponding criteria. Dots represent outliers. Two-tail t.test was used. Source data are provided as a Source Data file.

**Supplementary Fig. 8.** UMAPs of selected nine embeddings of the CITE-seq data. Each embedding showed signals that helped separate cell clusters. The sequence of embedding is not ranked. All 128 embedding figures can be found in the Additional material.

**Supplementary Fig. 9.** Gene-associated networks generated in the four clusters based on the CITE-seq case 1 data. Each dot presents a gene with high attention scores in the cluster. Each edge represents the correlation coefficient of the HGT embedding of the two genes—a higher correlation coefficient results in a thicker edge. Only correlation coefficients higher than 0.8 were kept.

**Supplementary Fig. 10.** Regulon activity heatmap of regulons in DSLL state-1 that are differentially active compared to normal B cells and DSLL state-2. Source data are provided as a Source Data file.

**Supplementary Fig. 11.** Gene expression, chromatin accessibility, GAS, and attention score of genes regulated by JUN in DSLL-1 and DSLL-2 states. Genes were compared among normal B cells (blue), DSLL state-1 (green), and DSLL state-2 (pink).

**Supplementary Fig. 12.** Gene expression, chromatin accessibility, GAS, and attention score of genes regulated by JUN in normal B cells and shared between two DSLL states. Genes were compared among normal B cells (blue), DSLL state-1 (green), and DSLL state-2 (pink).
