# Supplementary material for: Single-cell biological network inference using a heterogeneous graph transformer
Source: Nat Commun. 2023 Feb 21;14:964. doi: 10.1038/s41467-023-36559-0 (PMC9944243; doi:10.1038/s41467-023-36559-0)
Supplement: Supplementary file 3 — Description to Additional Supplementary Information [file 41467_2023_36559_MOESM3_ESM.pdf]

Description of Additional Supplementary Files

Supplementary Data 1. All datasets collection in DeepMAPS.

Supplementary Data 2. Curated marker for cell cluster annotation in CITE-seq case study

Supplementary Data 3. UMAPs of all 128 embeddings of CITE-seq case data

Supplementary Data 4. Curated marker genes for cell cluster annotation in the scRNA ATAC-seq case study

Supplementary Data 5. Comparison of public single-cell analysis servers

Supplementary Data 6. Computing time comparison (h/m/s)
