# Supplementary figures and images for: Single-cell biological network inference using a heterogeneous graph transformer (part 2 of 2)
Source: Nat Commun. 2023 Feb 21;14:964. doi: 10.1038/s41467-023-36559-0 (PMC9944243; doi:10.1038/s41467-023-36559-0)

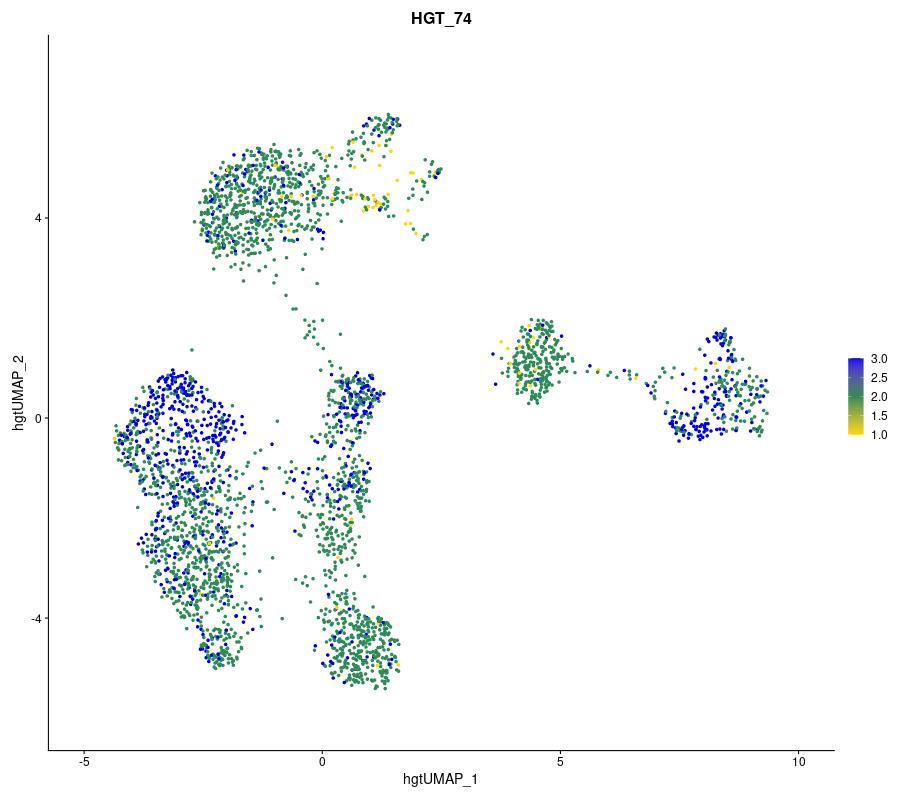

Supplement: Supplementary file 5 — Supplementary Data 3 [file 41467_2023_36559_MOESM5_ESM.zip › all128embedding/74 .jpg]

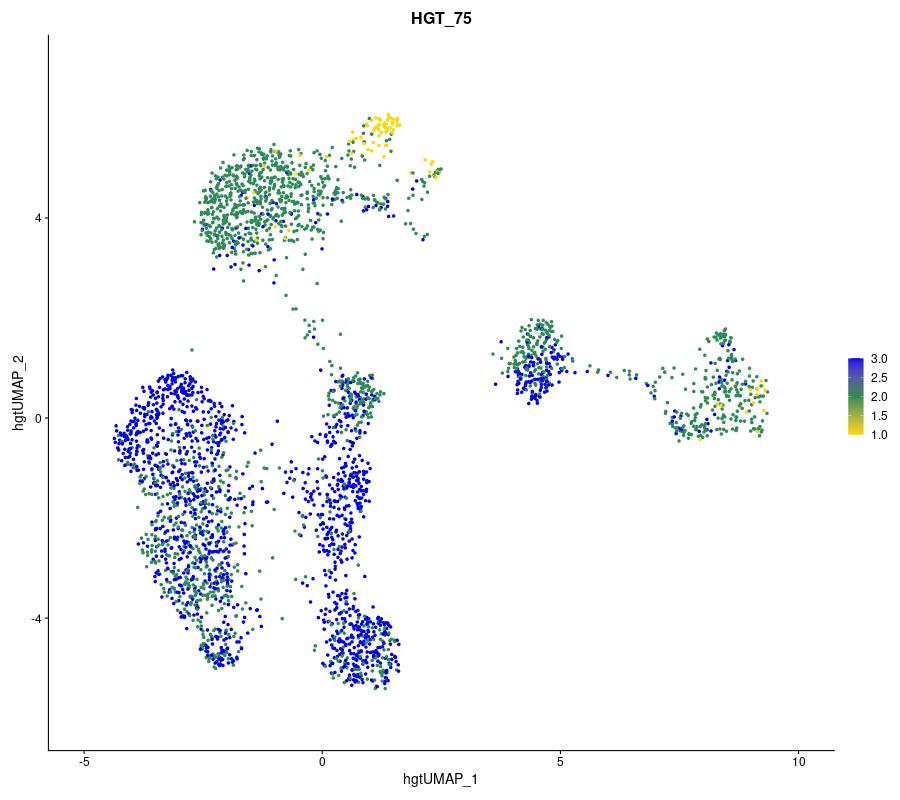

Supplement: Supplementary file 5 — Supplementary Data 3 [file 41467_2023_36559_MOESM5_ESM.zip › all128embedding/75 .jpg]

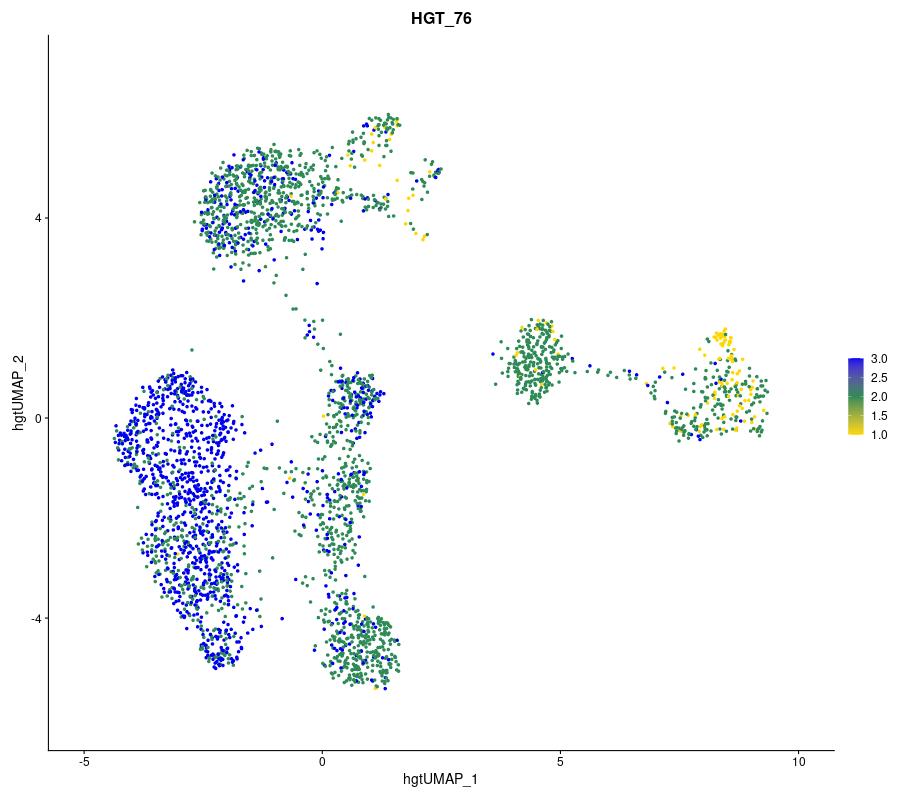

Supplement: Supplementary file 5 — Supplementary Data 3 [file 41467_2023_36559_MOESM5_ESM.zip › all128embedding/76 .jpg]

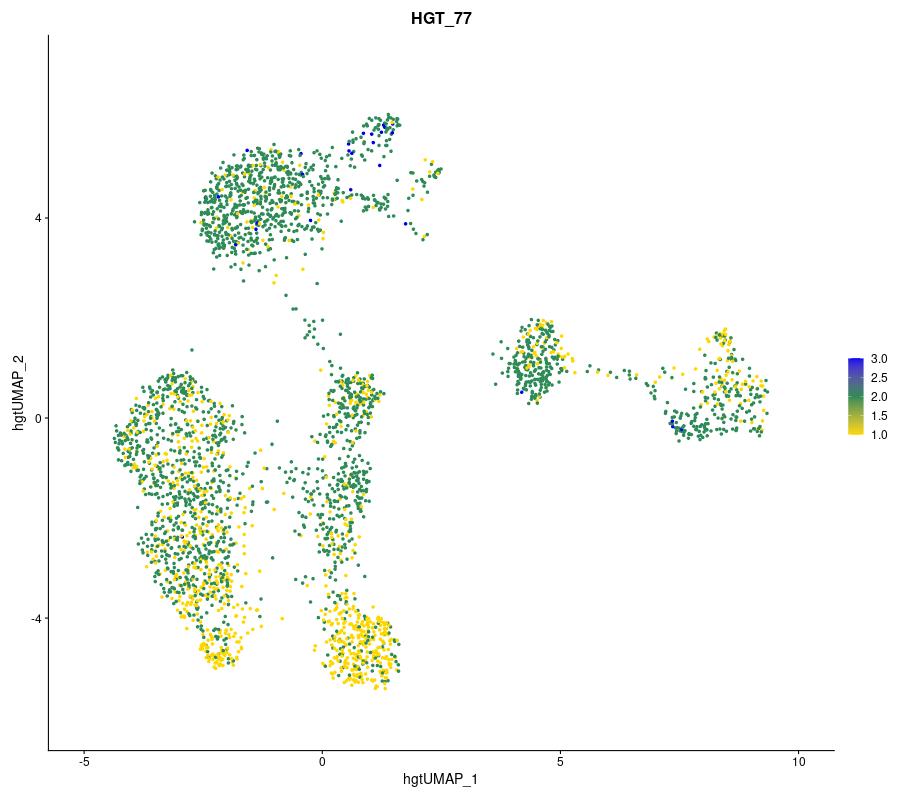

Supplement: Supplementary file 5 — Supplementary Data 3 [file 41467_2023_36559_MOESM5_ESM.zip › all128embedding/77 .jpg]

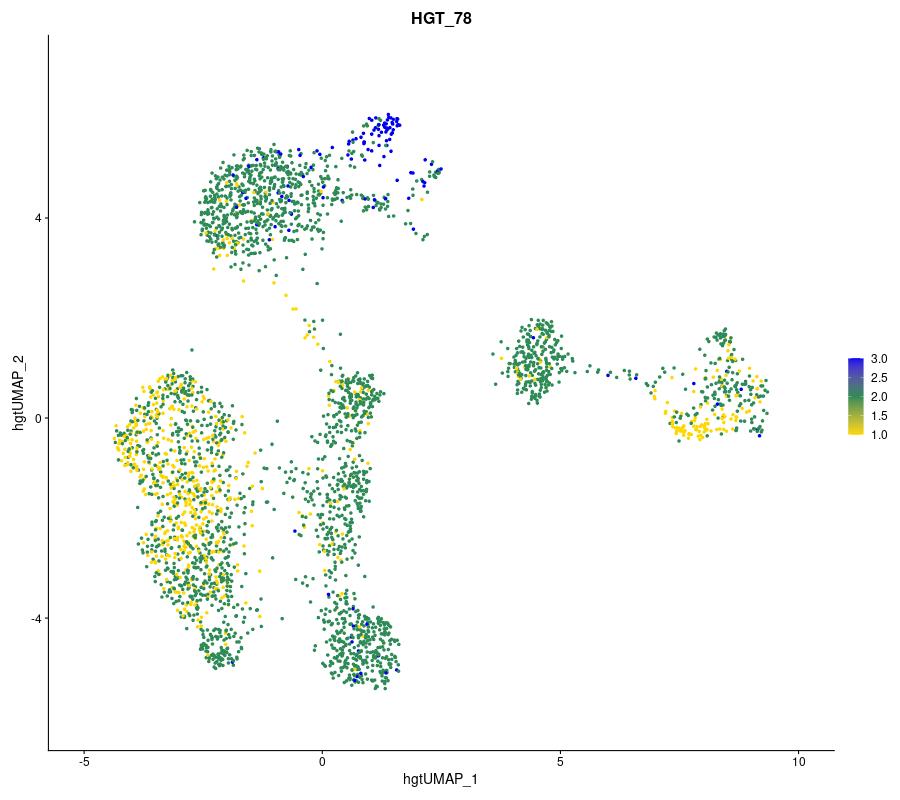

Supplement: Supplementary file 5 — Supplementary Data 3 [file 41467_2023_36559_MOESM5_ESM.zip › all128embedding/78 .jpg]

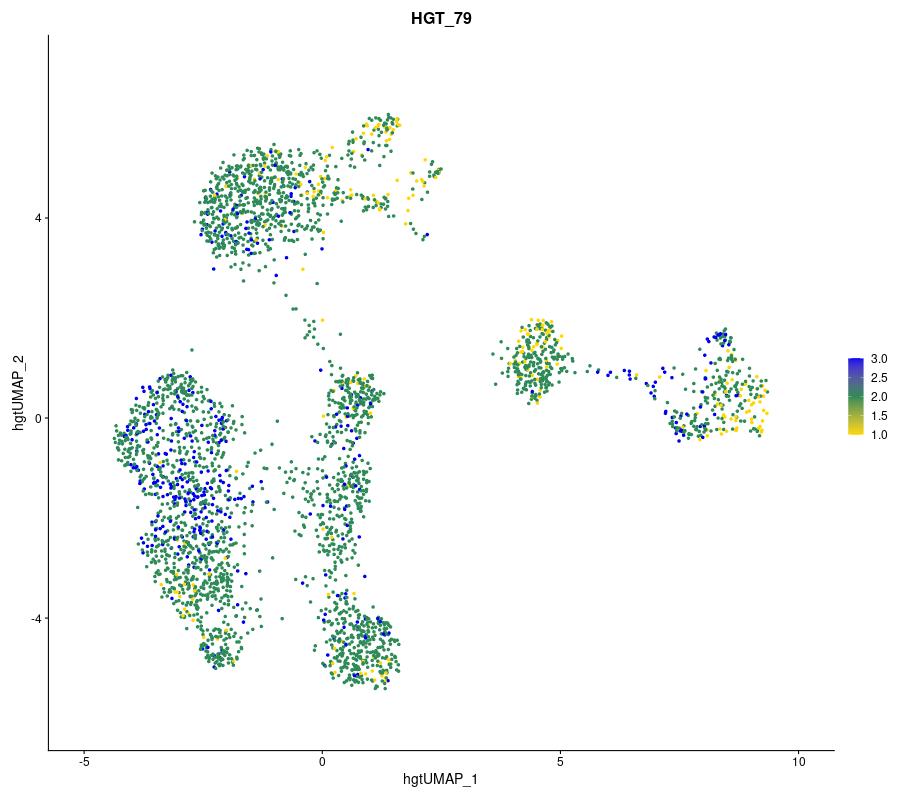

Supplement: Supplementary file 5 — Supplementary Data 3 [file 41467_2023_36559_MOESM5_ESM.zip › all128embedding/79 .jpg]

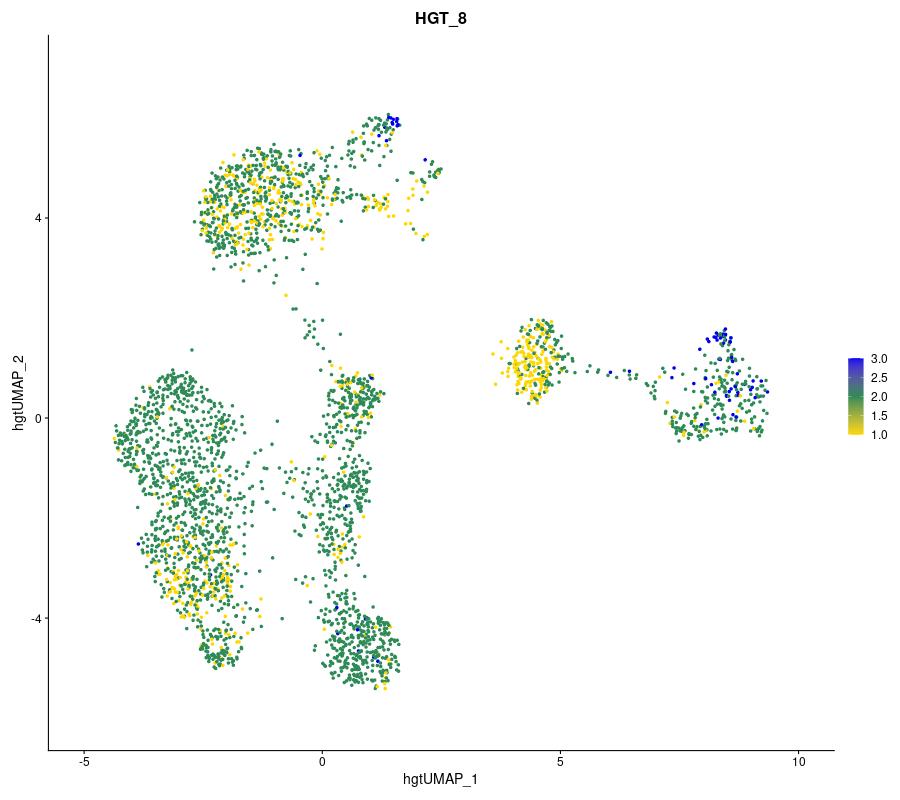

Supplement: Supplementary file 5 — Supplementary Data 3 [file 41467_2023_36559_MOESM5_ESM.zip › all128embedding/8 .jpg]

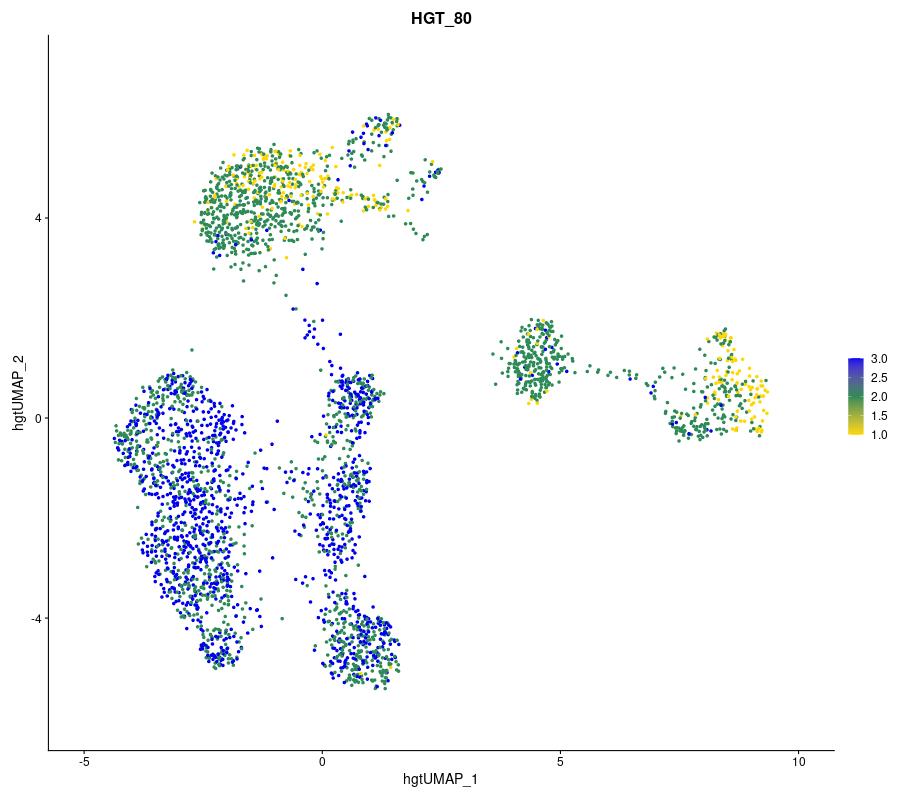

Supplement: Supplementary file 5 — Supplementary Data 3 [file 41467_2023_36559_MOESM5_ESM.zip › all128embedding/80 .jpg]

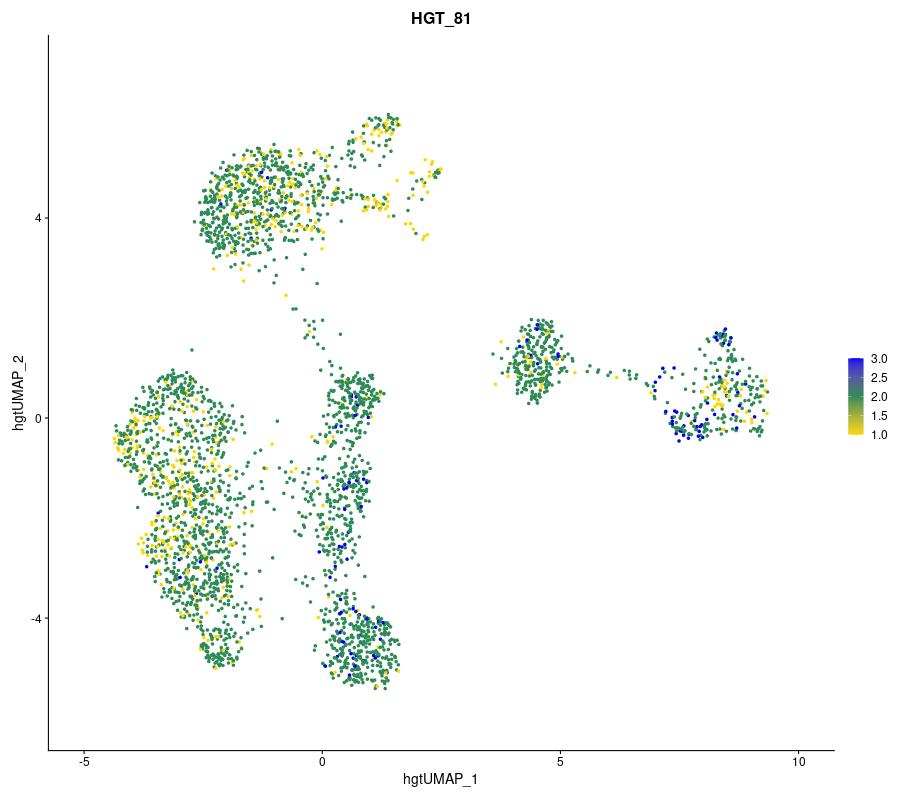

Supplement: Supplementary file 5 — Supplementary Data 3 [file 41467_2023_36559_MOESM5_ESM.zip › all128embedding/81 .jpg]

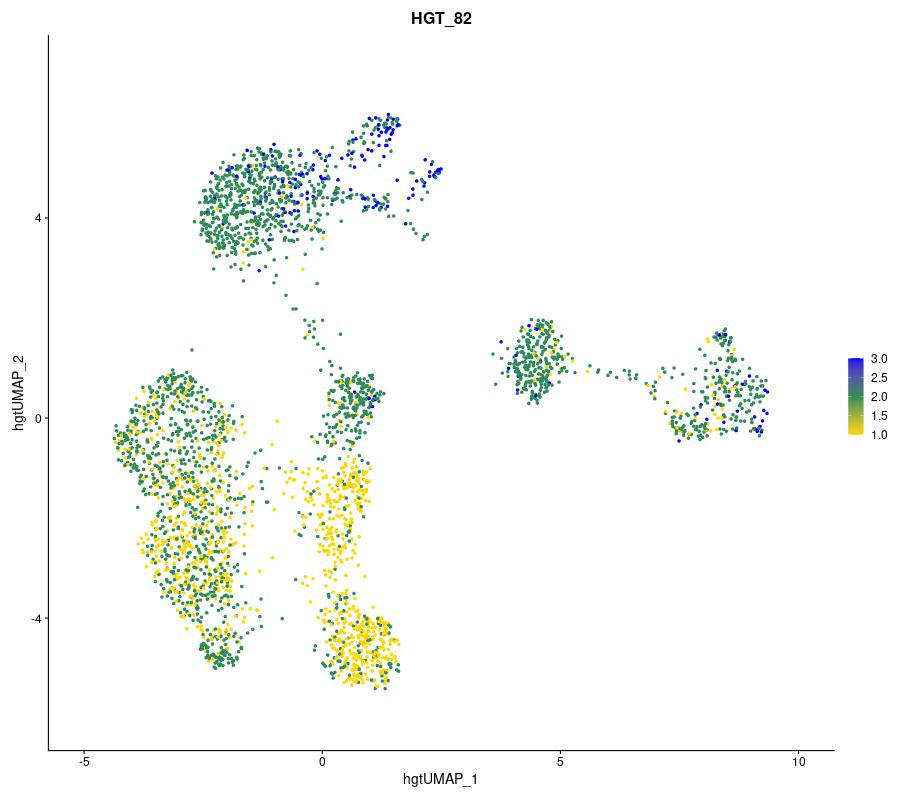

Supplement: Supplementary file 5 — Supplementary Data 3 [file 41467_2023_36559_MOESM5_ESM.zip › all128embedding/82 .jpg]

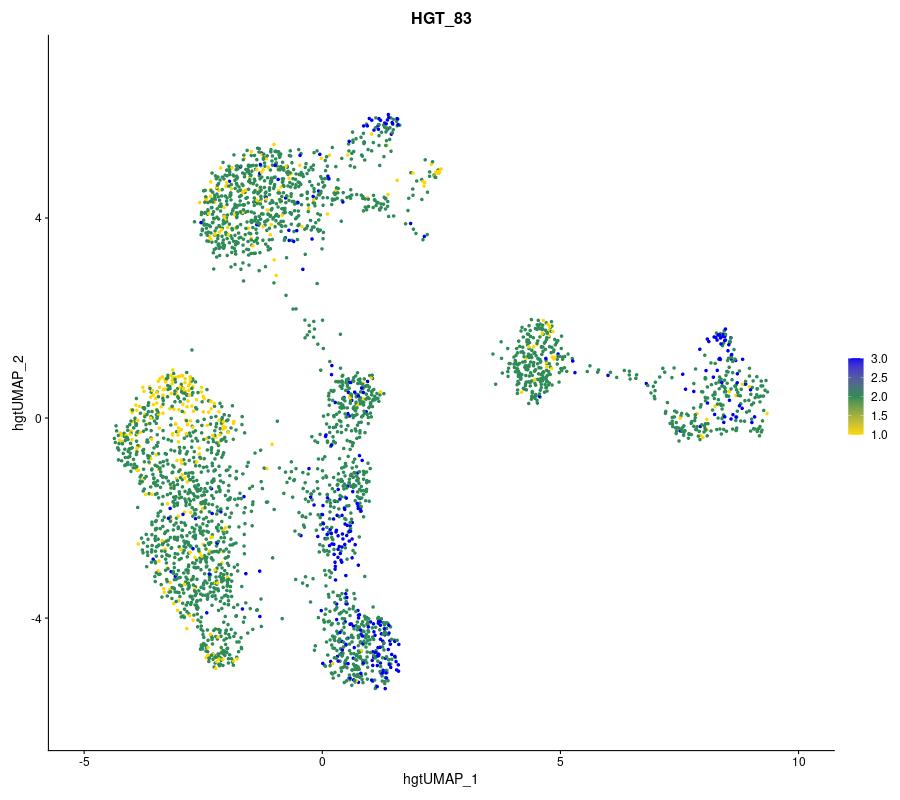

Supplement: Supplementary file 5 — Supplementary Data 3 [file 41467_2023_36559_MOESM5_ESM.zip › all128embedding/83 .jpg]

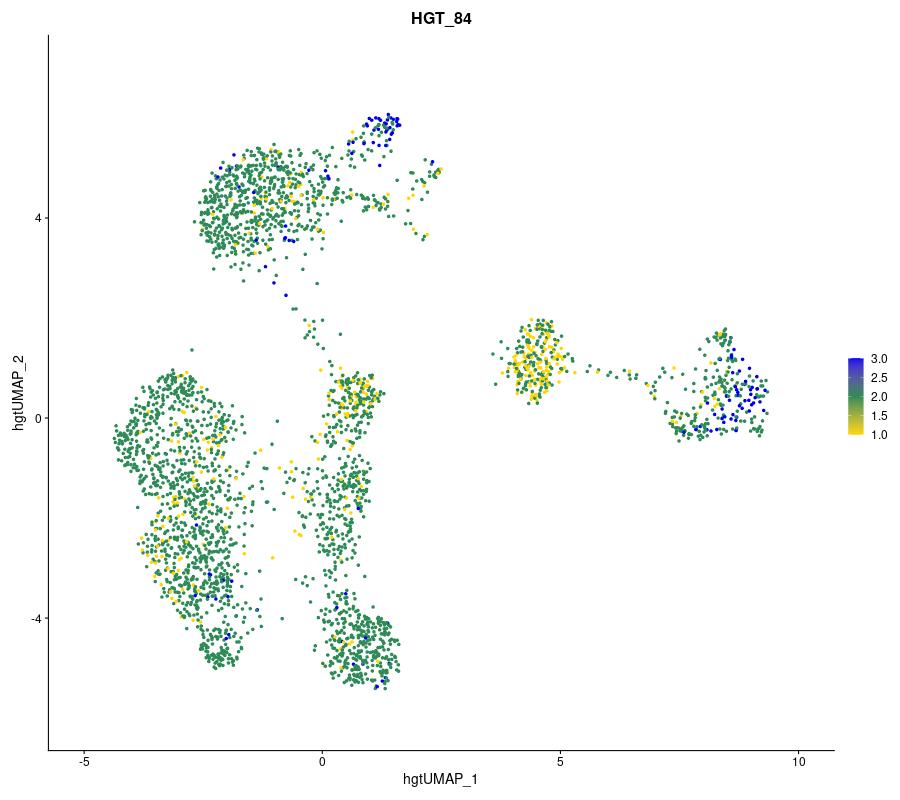

Supplement: Supplementary file 5 — Supplementary Data 3 [file 41467_2023_36559_MOESM5_ESM.zip › all128embedding/84 .jpg]

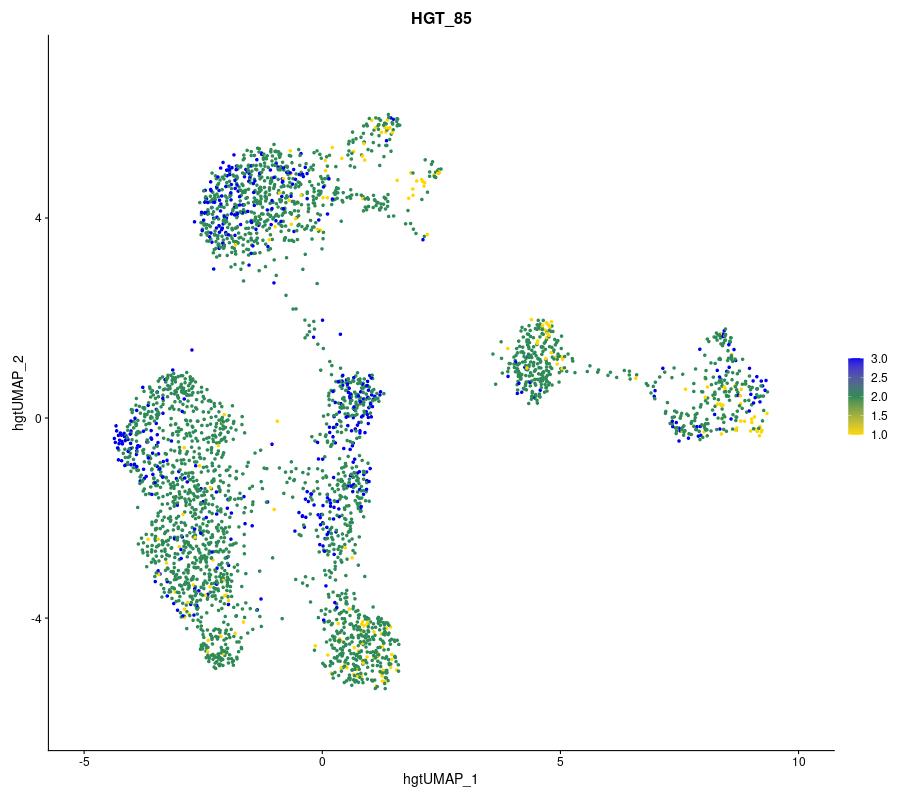

Supplement: Supplementary file 5 — Supplementary Data 3 [file 41467_2023_36559_MOESM5_ESM.zip › all128embedding/85 .jpg]

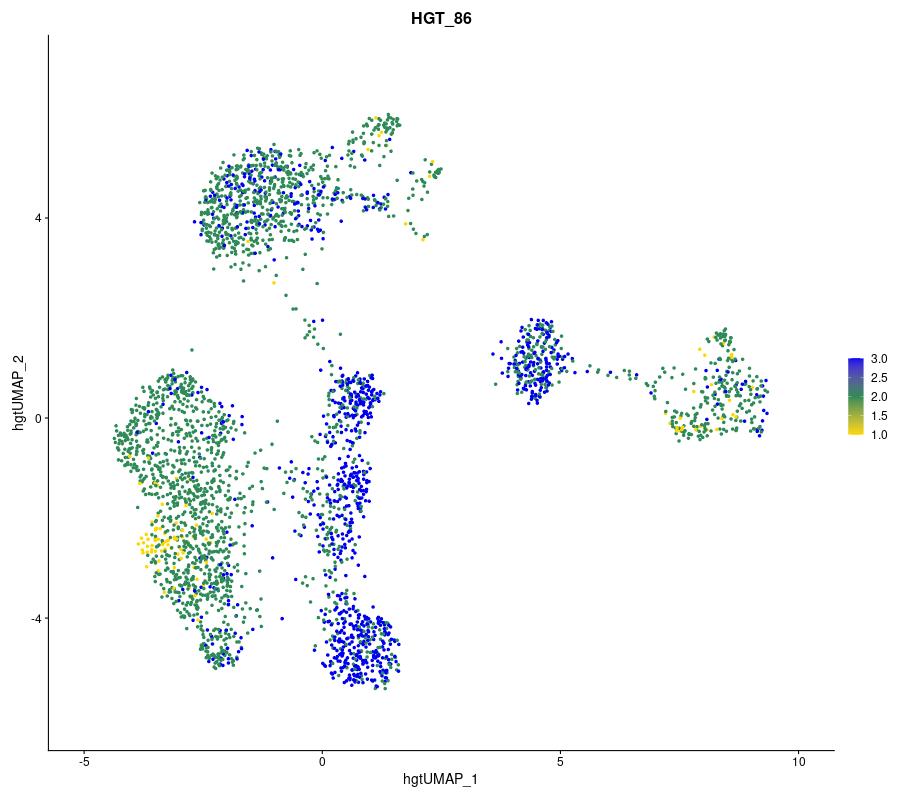

Supplement: Supplementary file 5 — Supplementary Data 3 [file 41467_2023_36559_MOESM5_ESM.zip › all128embedding/86 .jpg]

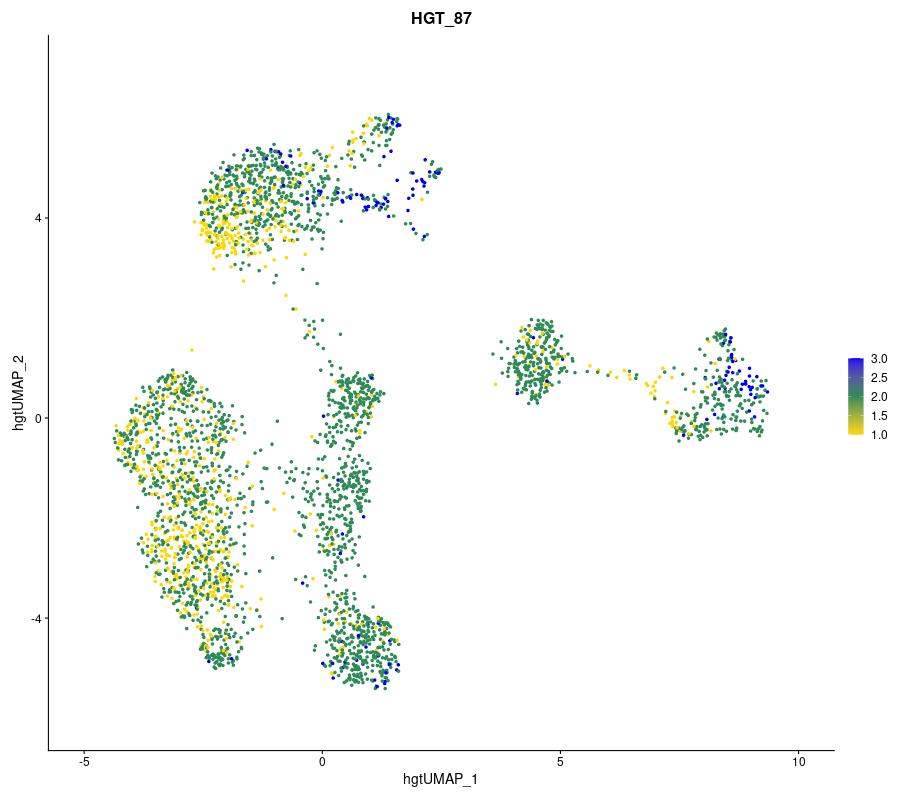

Supplement: Supplementary file 5 — Supplementary Data 3 [file 41467_2023_36559_MOESM5_ESM.zip › all128embedding/87 .jpg]

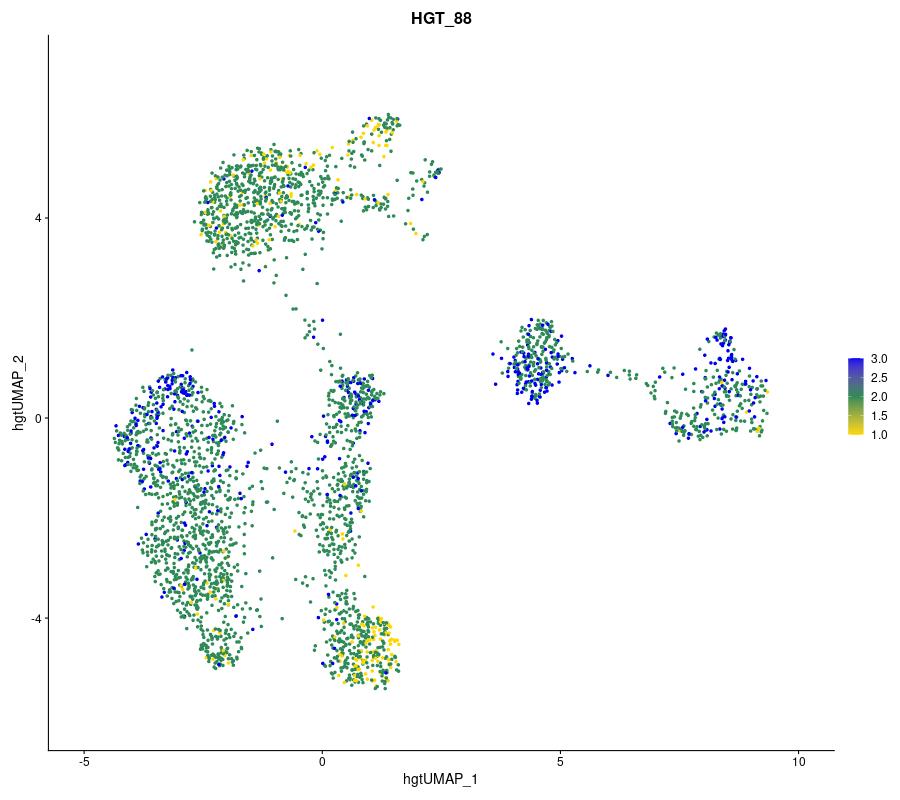

Supplement: Supplementary file 5 — Supplementary Data 3 [file 41467_2023_36559_MOESM5_ESM.zip › all128embedding/88 .jpg]

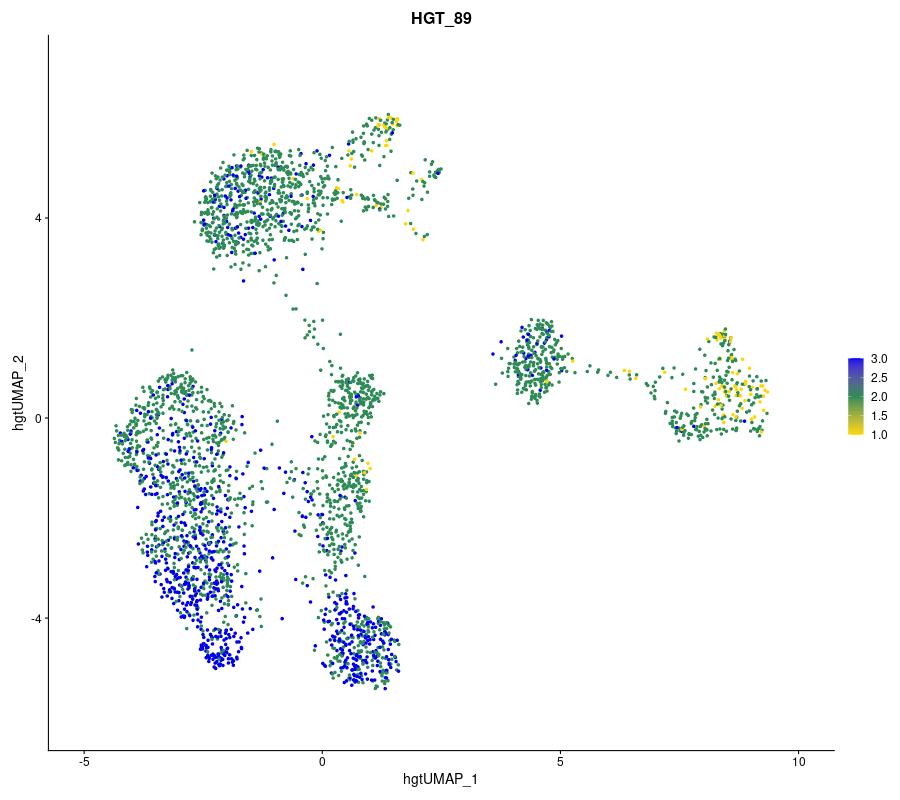

Supplement: Supplementary file 5 — Supplementary Data 3 [file 41467_2023_36559_MOESM5_ESM.zip › all128embedding/89 .jpg]

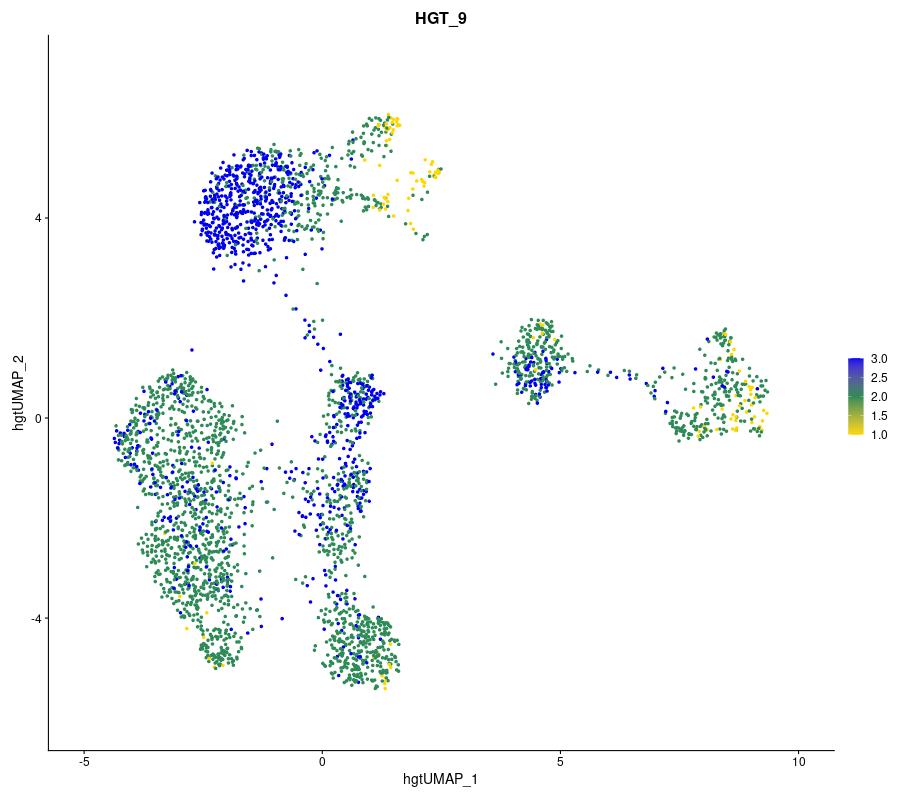

Supplement: Supplementary file 5 — Supplementary Data 3 [file 41467_2023_36559_MOESM5_ESM.zip › all128embedding/9 .jpg]

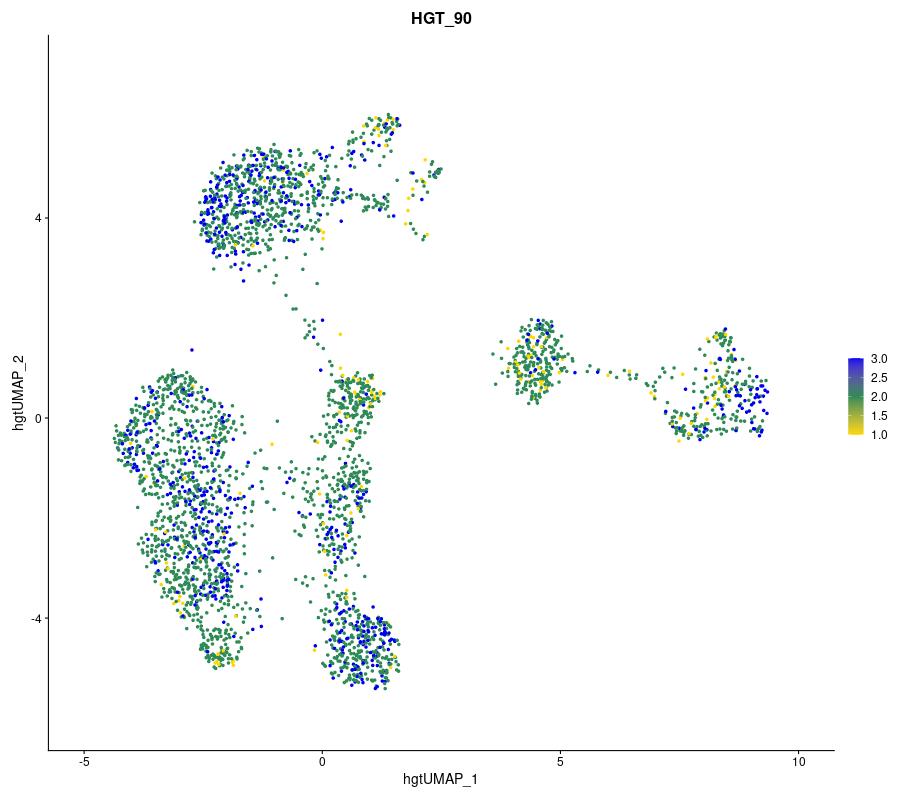

Supplement: Supplementary file 5 — Supplementary Data 3 [file 41467_2023_36559_MOESM5_ESM.zip › all128embedding/90 .jpg]

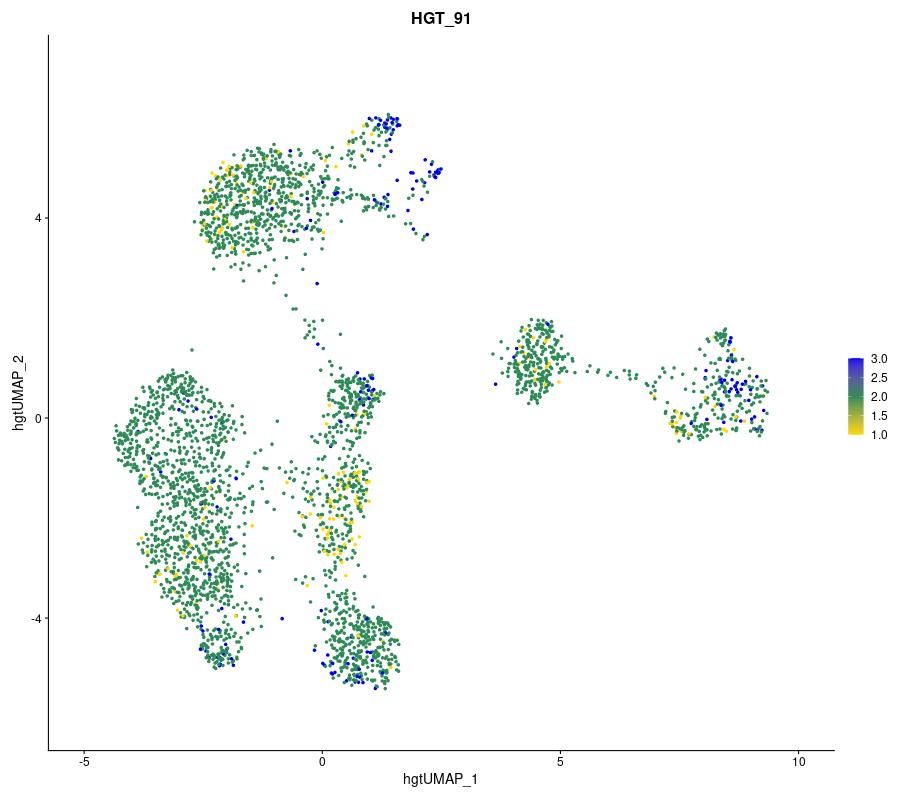

Supplement: Supplementary file 5 — Supplementary Data 3 [file 41467_2023_36559_MOESM5_ESM.zip › all128embedding/91 .jpg]

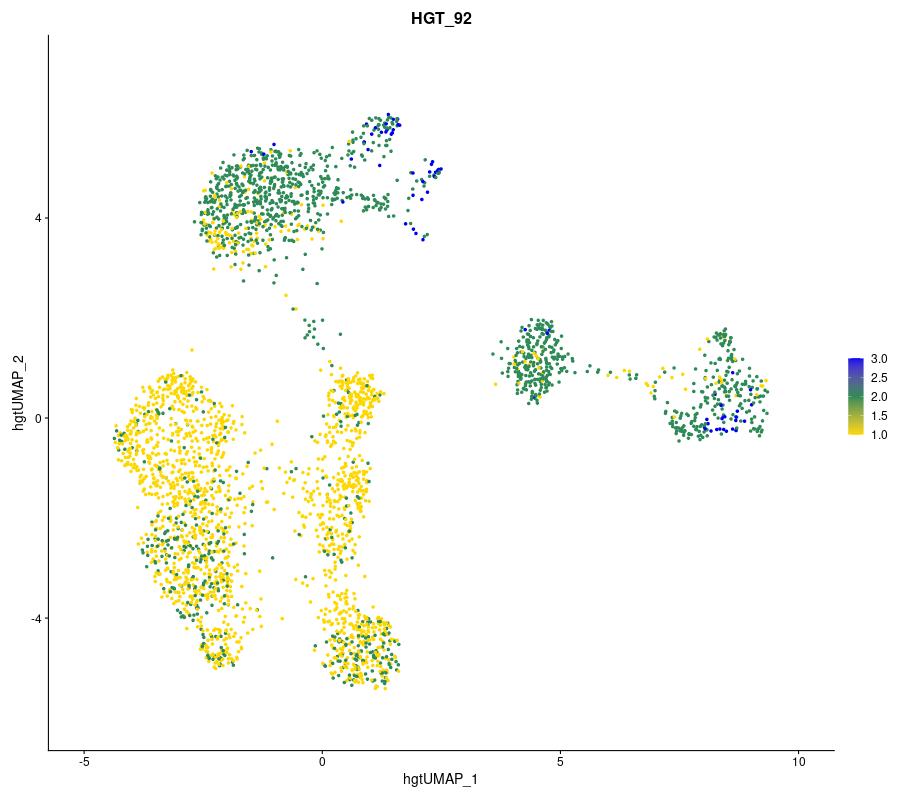

Supplement: Supplementary file 5 — Supplementary Data 3 [file 41467_2023_36559_MOESM5_ESM.zip › all128embedding/92 .jpg]

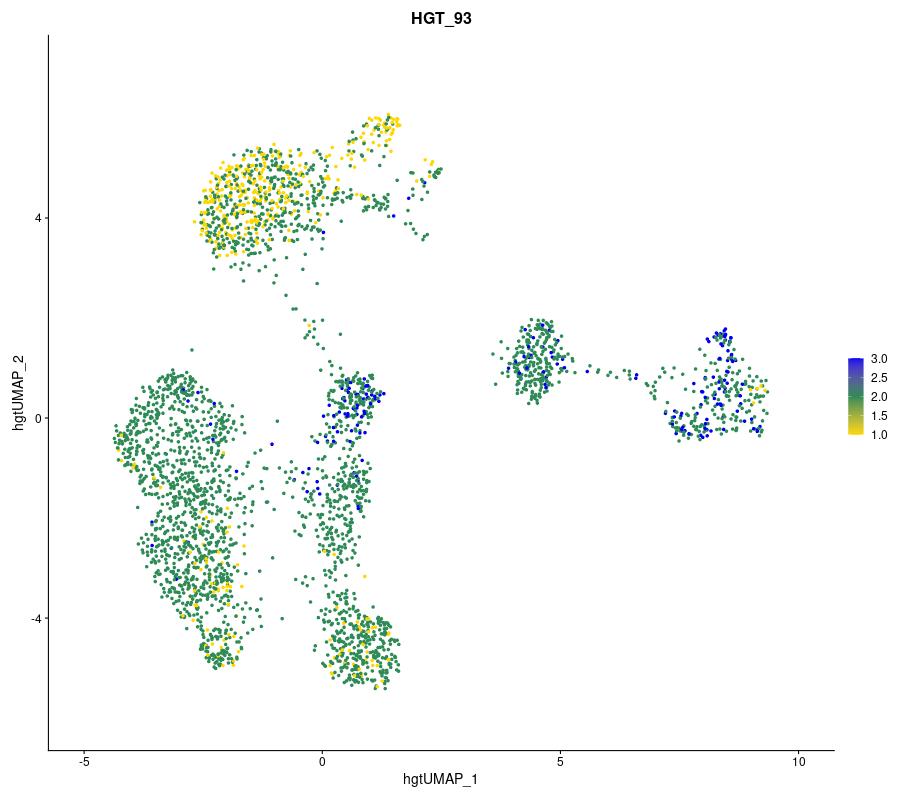

Supplement: Supplementary file 5 — Supplementary Data 3 [file 41467_2023_36559_MOESM5_ESM.zip › all128embedding/93 .jpg]

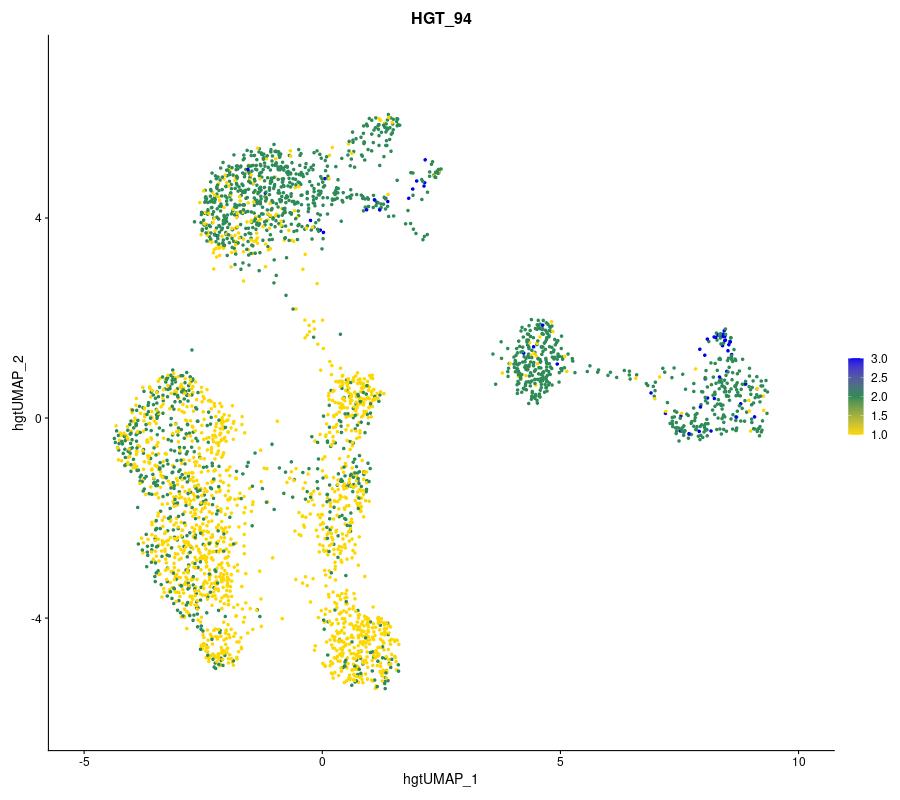

Supplement: Supplementary file 5 — Supplementary Data 3 [file 41467_2023_36559_MOESM5_ESM.zip › all128embedding/94 .jpg]

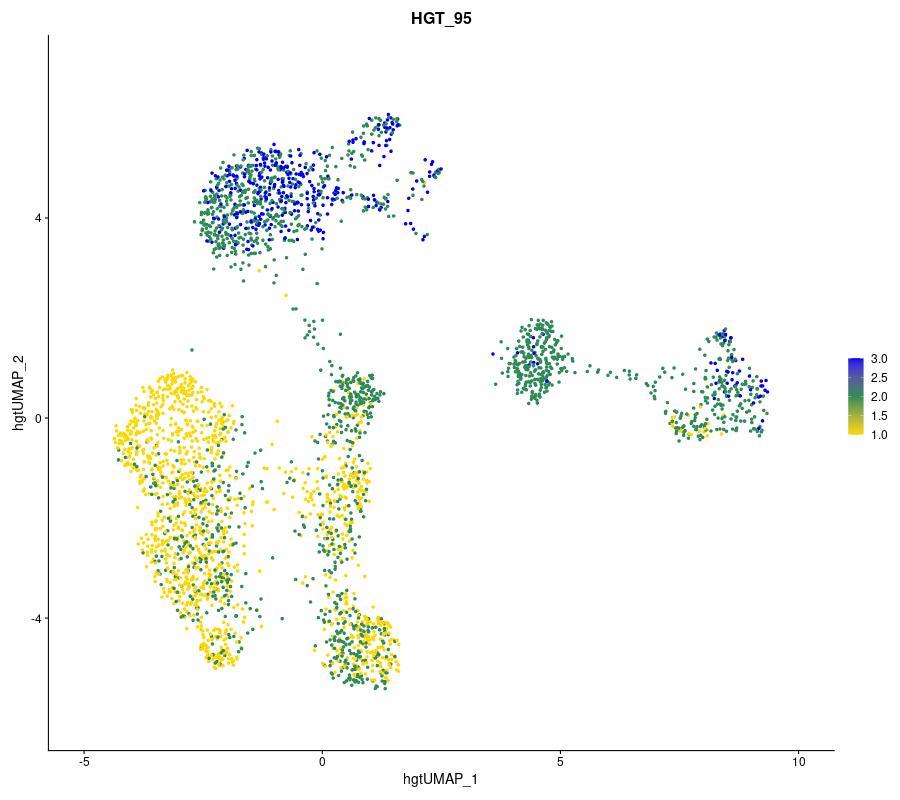

Supplement: Supplementary file 5 — Supplementary Data 3 [file 41467_2023_36559_MOESM5_ESM.zip › all128embedding/95 .jpg]

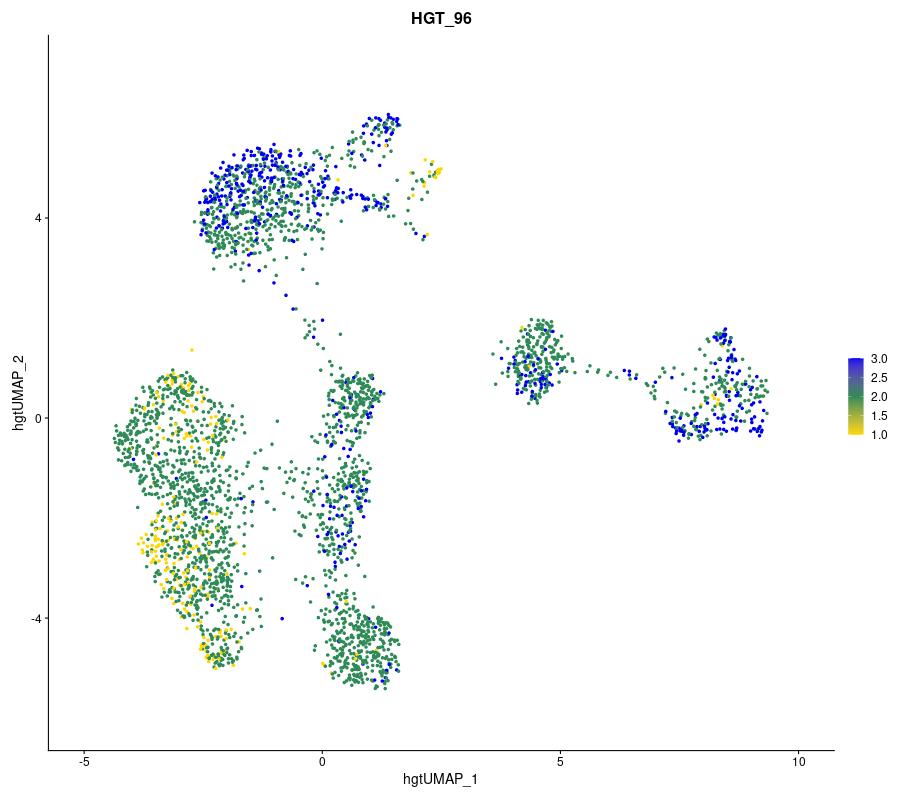

Supplement: Supplementary file 5 — Supplementary Data 3 [file 41467_2023_36559_MOESM5_ESM.zip › all128embedding/96 .jpg]

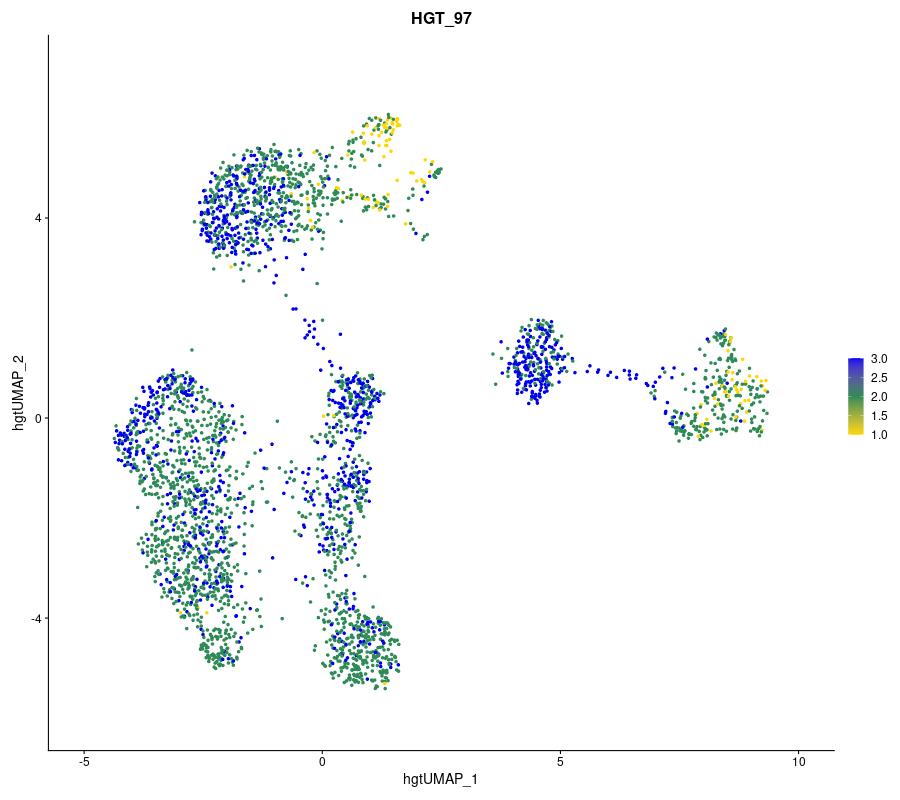

Supplement: Supplementary file 5 — Supplementary Data 3 [file 41467_2023_36559_MOESM5_ESM.zip › all128embedding/97 .jpg]

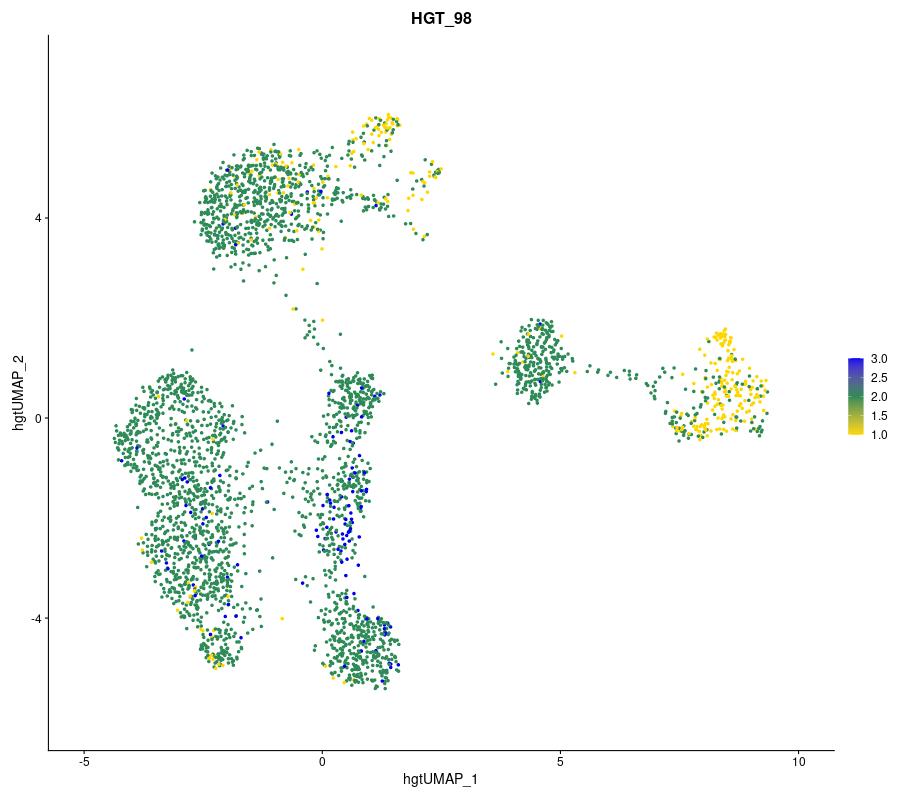

Supplement: Supplementary file 5 — Supplementary Data 3 [file 41467_2023_36559_MOESM5_ESM.zip › all128embedding/98 .jpg]

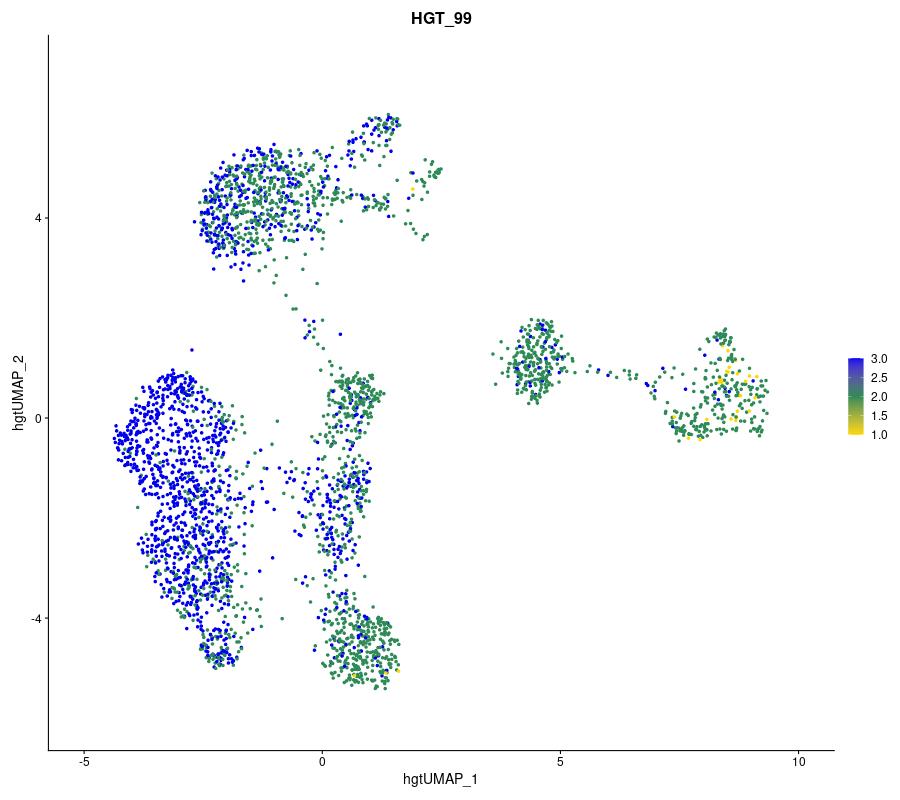

Supplement: Supplementary file 5 — Supplementary Data 3 [file 41467_2023_36559_MOESM5_ESM.zip › all128embedding/99 .jpg]
