# Supplementary figures and images for: Single-cell biological network inference using a heterogeneous graph transformer (part 1 of 2)
Source: Nat Commun. 2023 Feb 21;14:964. doi: 10.1038/s41467-023-36559-0 (PMC9944243; doi:10.1038/s41467-023-36559-0)

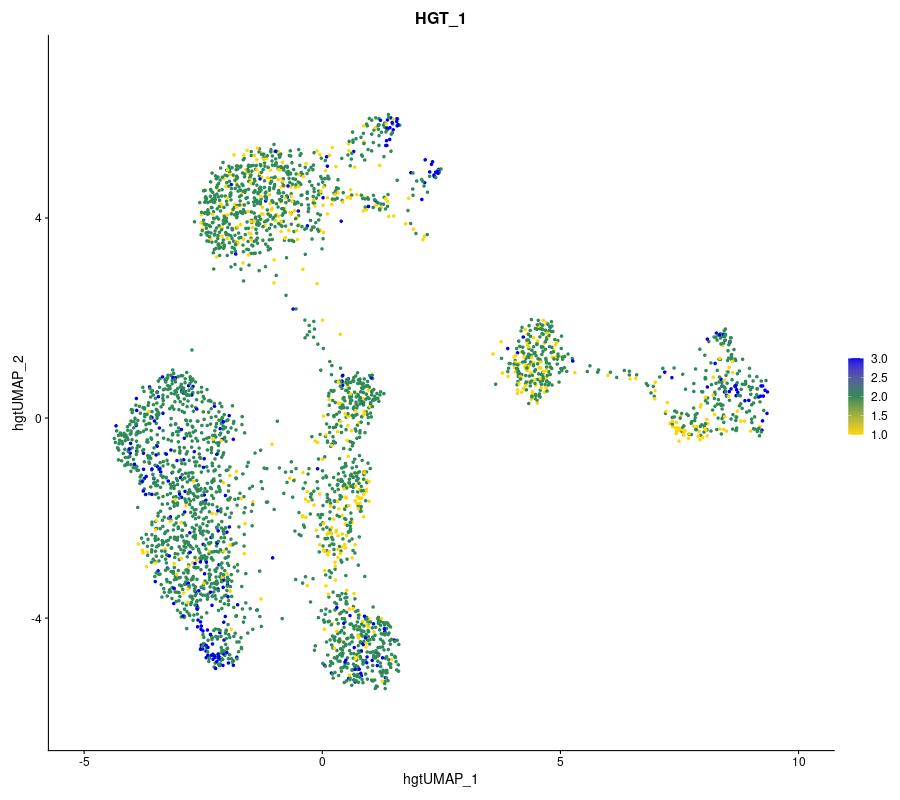

Supplement: Supplementary file 5 — Supplementary Data 3 [file 41467_2023_36559_MOESM5_ESM.zip › all128embedding/1 .jpg]

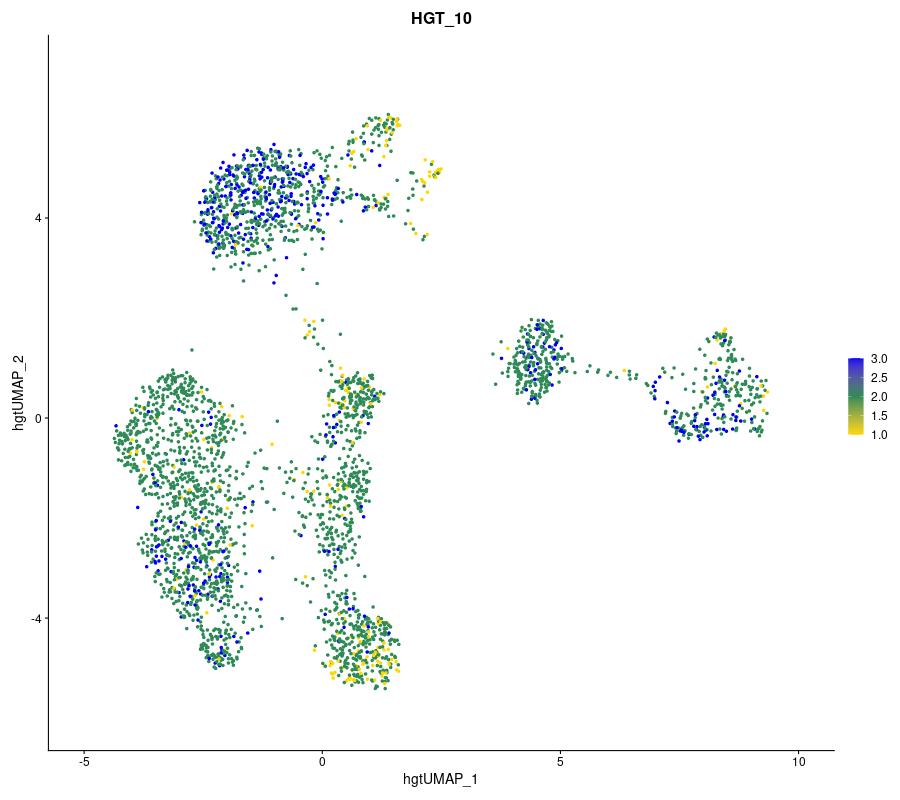

Supplement: Supplementary file 5 — Supplementary Data 3 [file 41467_2023_36559_MOESM5_ESM.zip › all128embedding/10 .jpg]

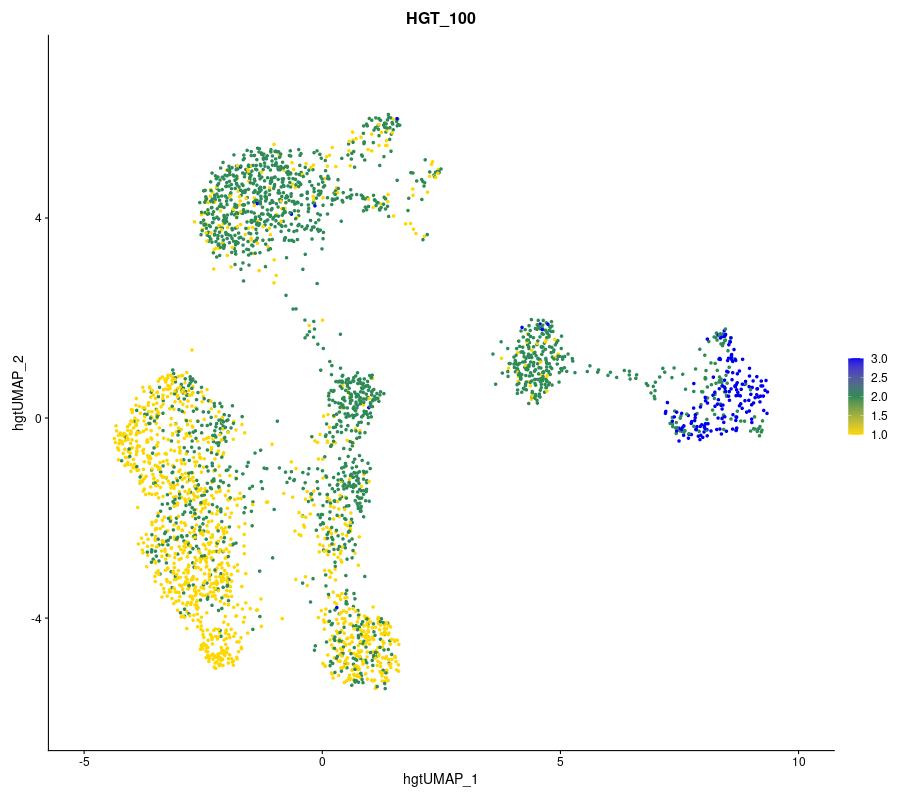

Supplement: Supplementary file 5 — Supplementary Data 3 [file 41467_2023_36559_MOESM5_ESM.zip › all128embedding/100 .jpg]

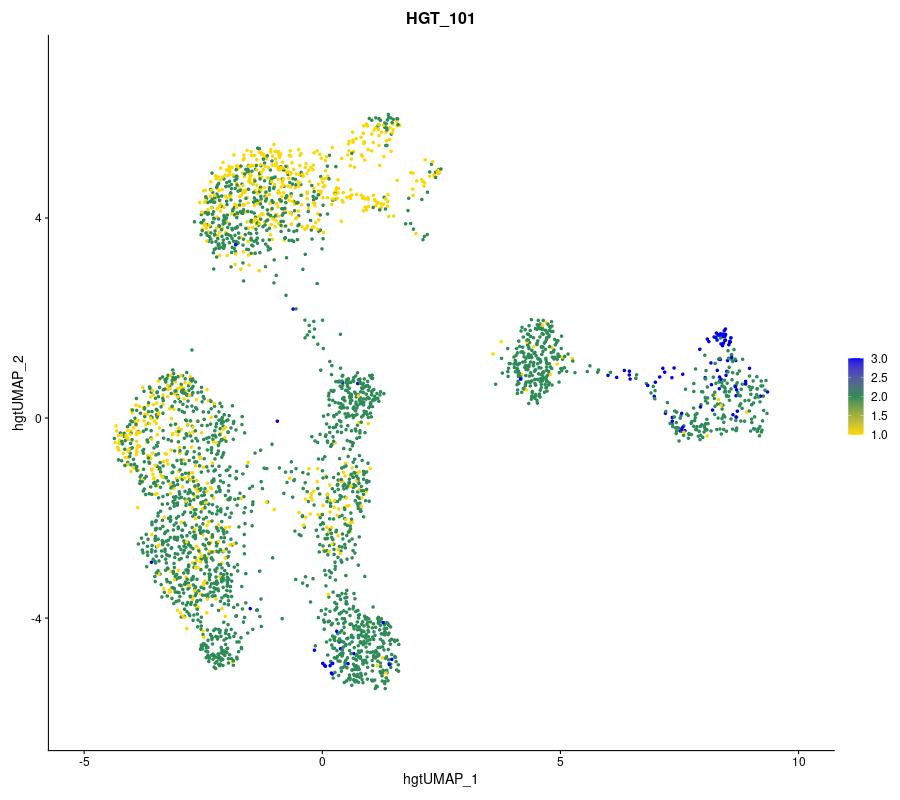

Supplement: Supplementary file 5 — Supplementary Data 3 [file 41467_2023_36559_MOESM5_ESM.zip › all128embedding/101 .jpg]

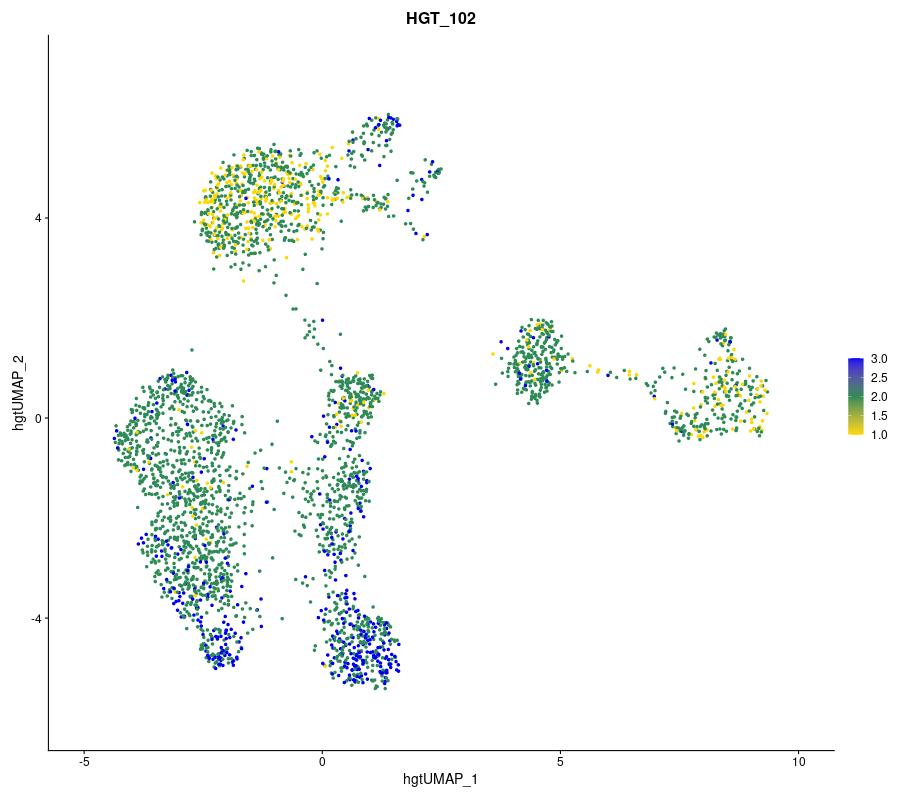

Supplement: Supplementary file 5 — Supplementary Data 3 [file 41467_2023_36559_MOESM5_ESM.zip › all128embedding/102 .jpg]

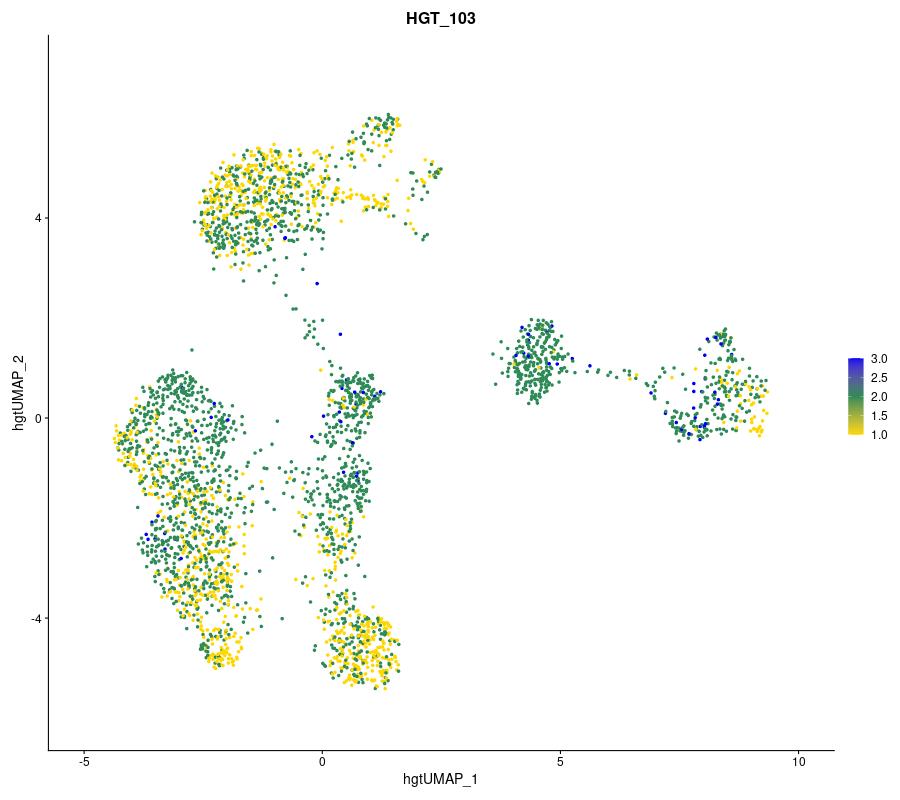

Supplement: Supplementary file 5 — Supplementary Data 3 [file 41467_2023_36559_MOESM5_ESM.zip › all128embedding/103 .jpg]

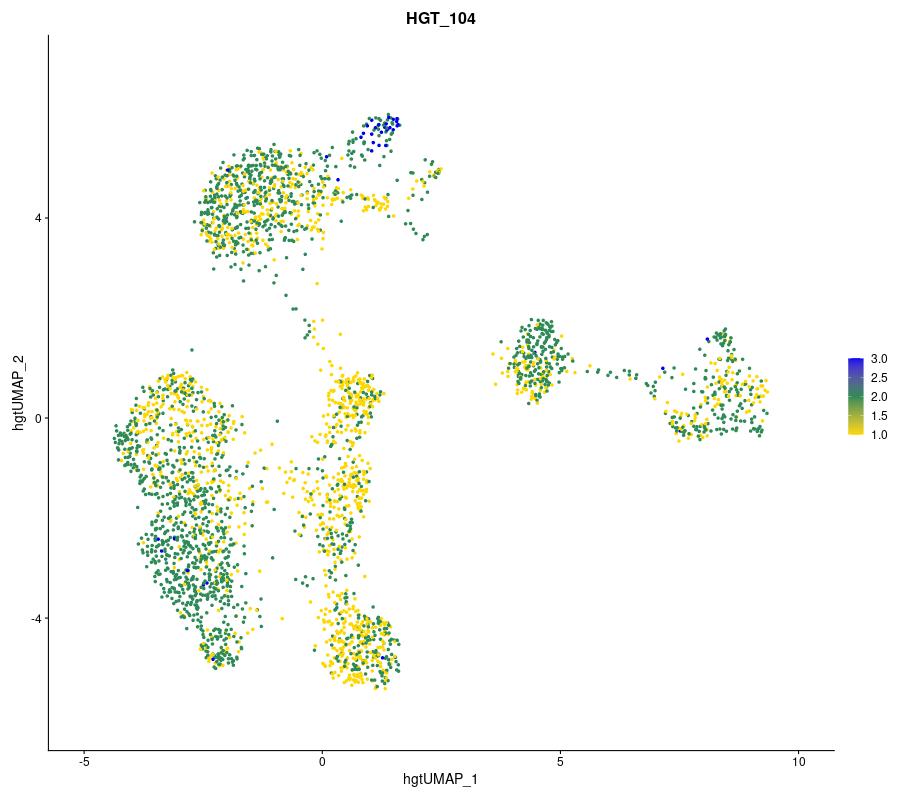

Supplement: Supplementary file 5 — Supplementary Data 3 [file 41467_2023_36559_MOESM5_ESM.zip › all128embedding/104 .jpg]

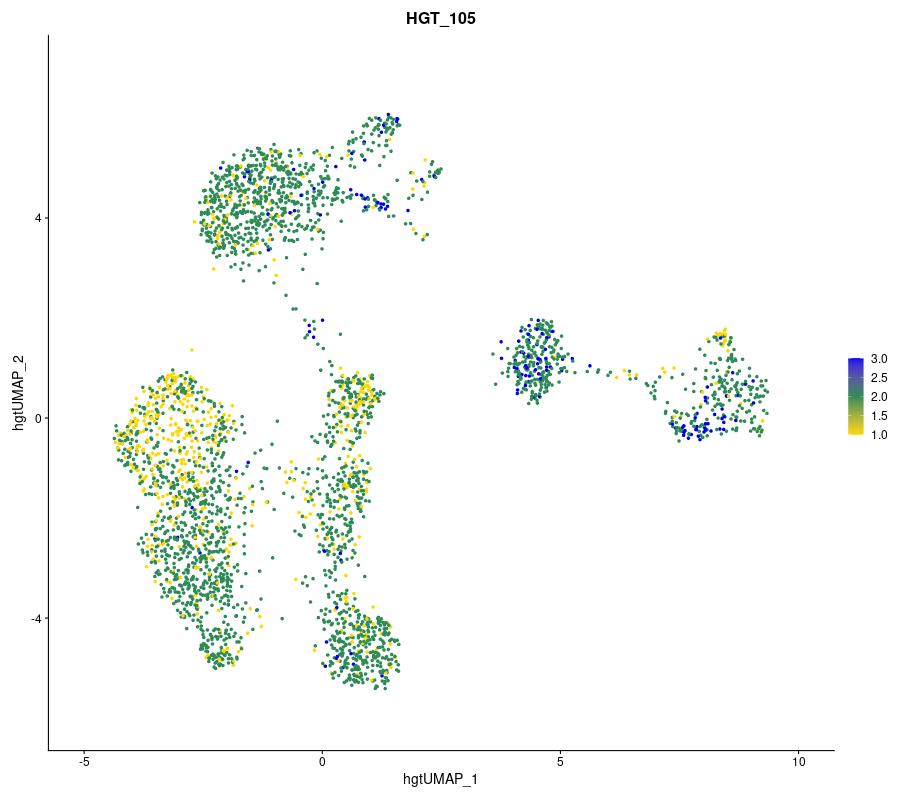

Supplement: Supplementary file 5 — Supplementary Data 3 [file 41467_2023_36559_MOESM5_ESM.zip › all128embedding/105 .jpg]

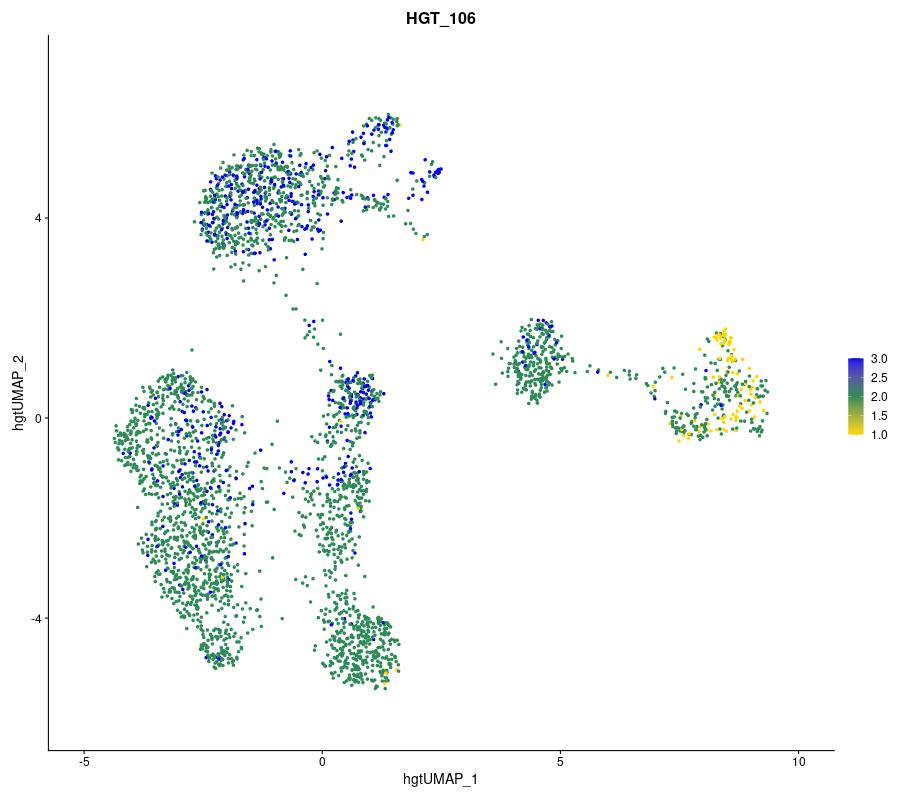

Supplement: Supplementary file 5 — Supplementary Data 3 [file 41467_2023_36559_MOESM5_ESM.zip › all128embedding/106 .jpg]

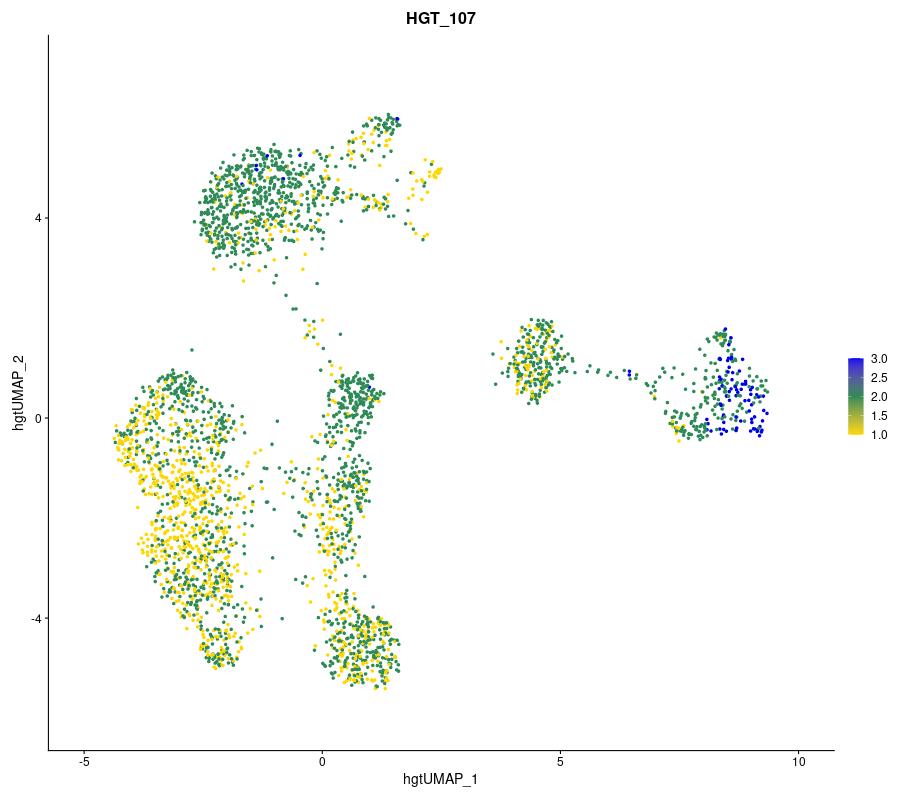

Supplement: Supplementary file 5 — Supplementary Data 3 [file 41467_2023_36559_MOESM5_ESM.zip › all128embedding/107 .jpg]

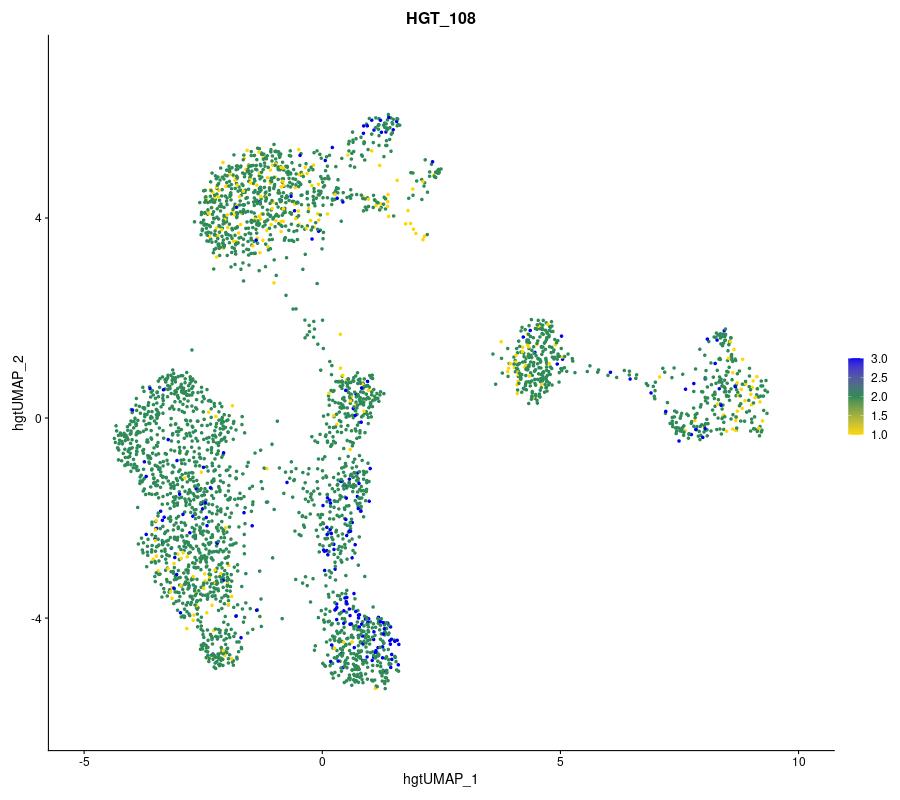

Supplement: Supplementary file 5 — Supplementary Data 3 [file 41467_2023_36559_MOESM5_ESM.zip › all128embedding/108 .jpg]

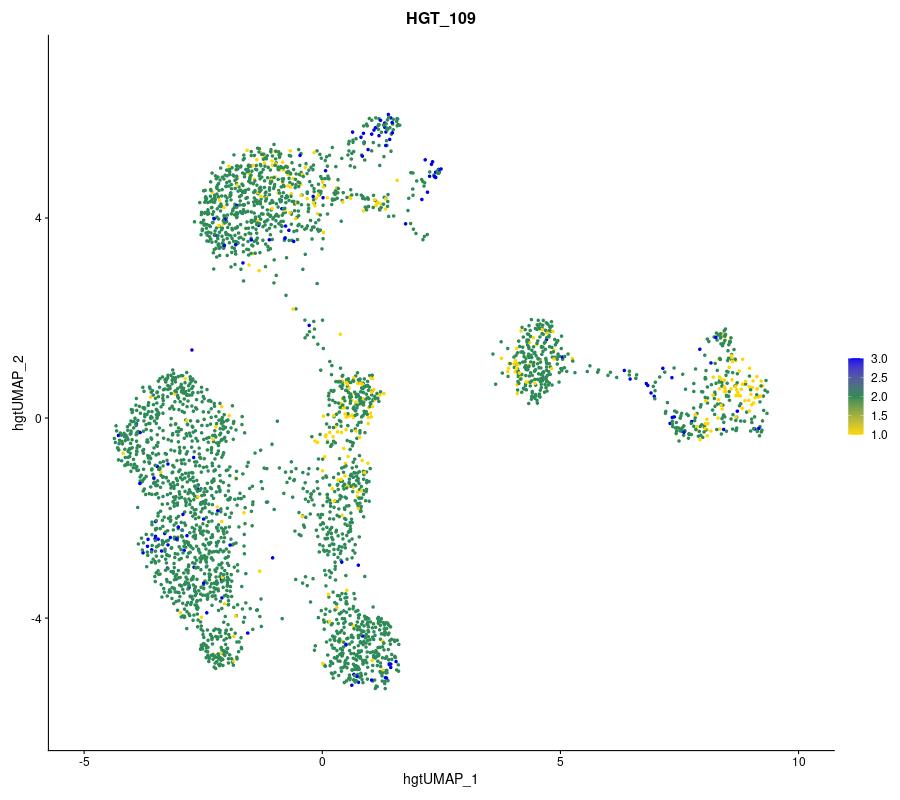

Supplement: Supplementary file 5 — Supplementary Data 3 [file 41467_2023_36559_MOESM5_ESM.zip › all128embedding/109 .jpg]

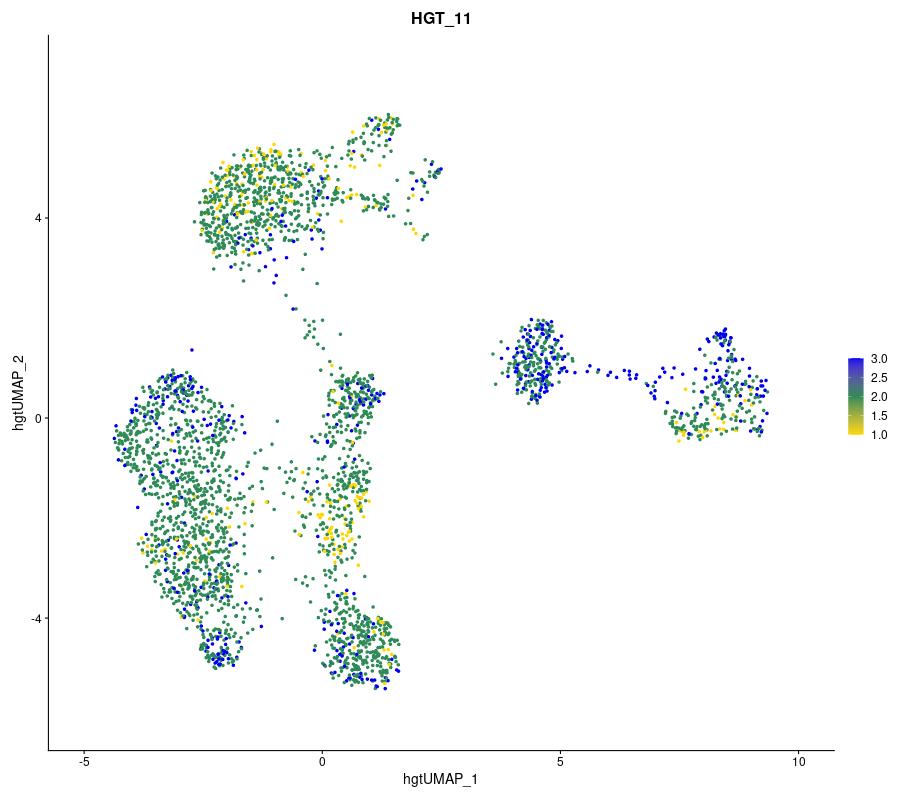

Supplement: Supplementary file 5 — Supplementary Data 3 [file 41467_2023_36559_MOESM5_ESM.zip › all128embedding/11 .jpg]

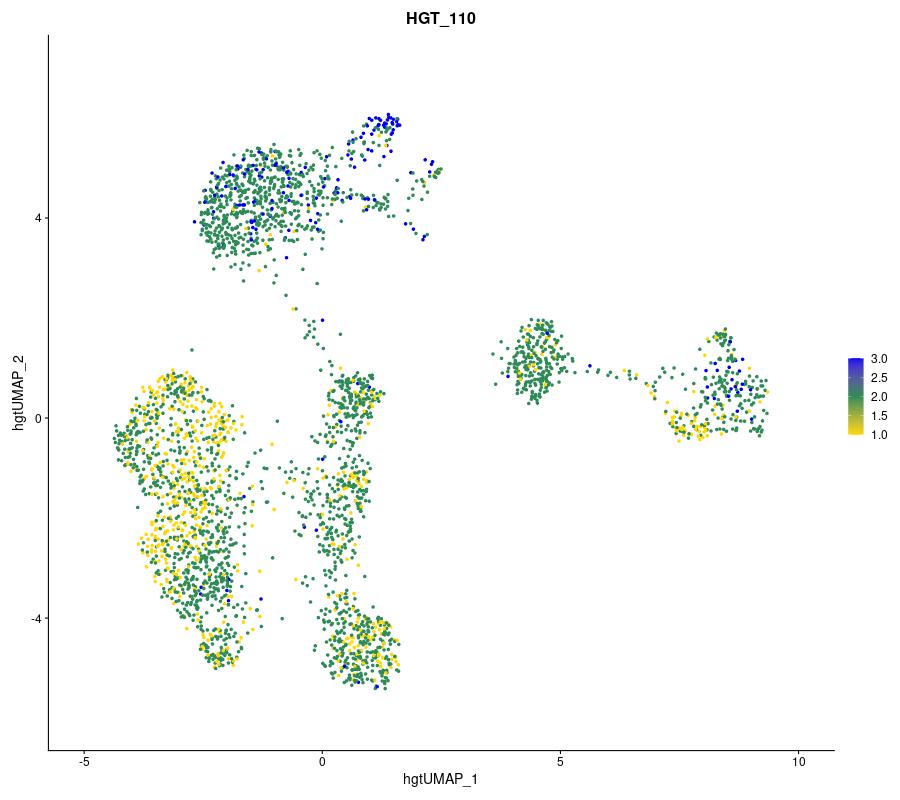

Supplement: Supplementary file 5 — Supplementary Data 3 [file 41467_2023_36559_MOESM5_ESM.zip › all128embedding/110 .jpg]

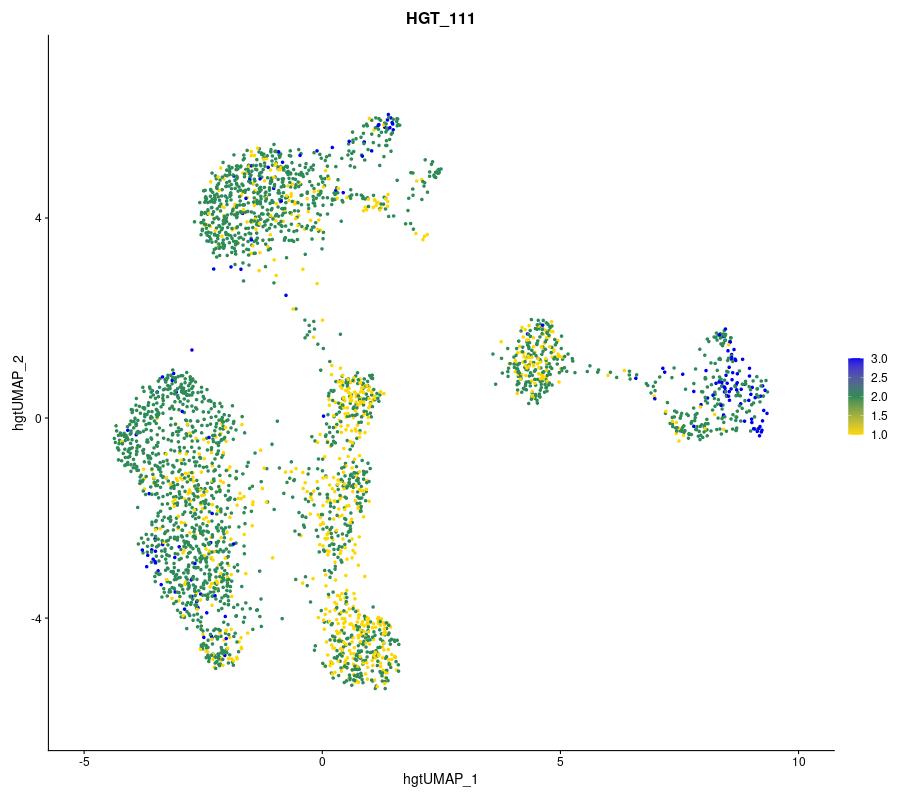

Supplement: Supplementary file 5 — Supplementary Data 3 [file 41467_2023_36559_MOESM5_ESM.zip › all128embedding/111 .jpg]

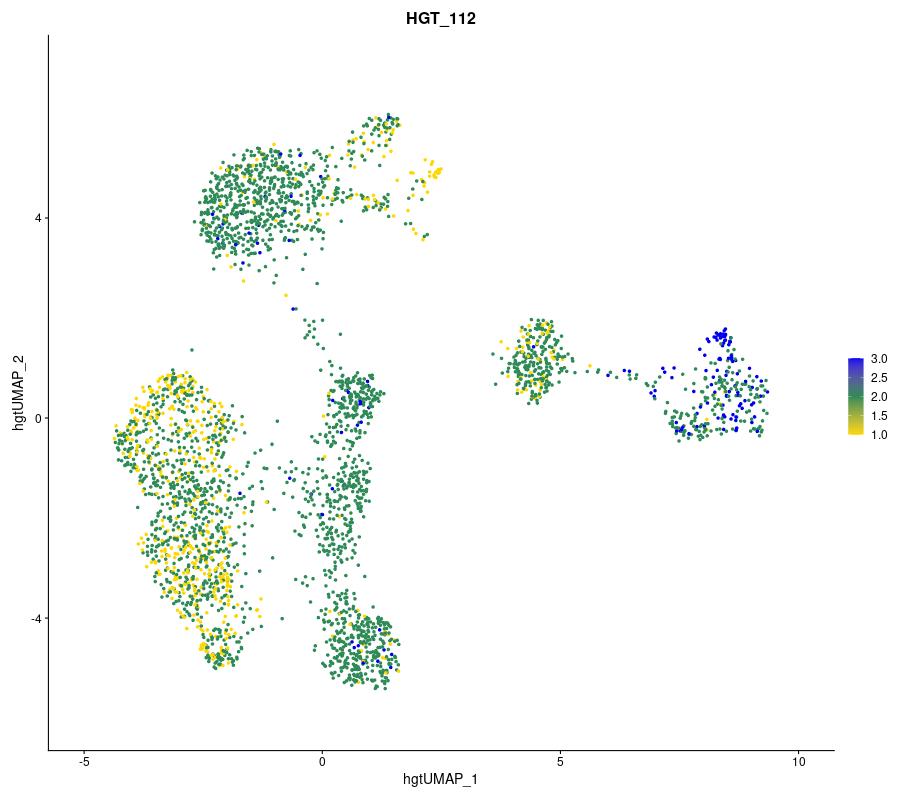

Supplement: Supplementary file 5 — Supplementary Data 3 [file 41467_2023_36559_MOESM5_ESM.zip › all128embedding/112 .jpg]

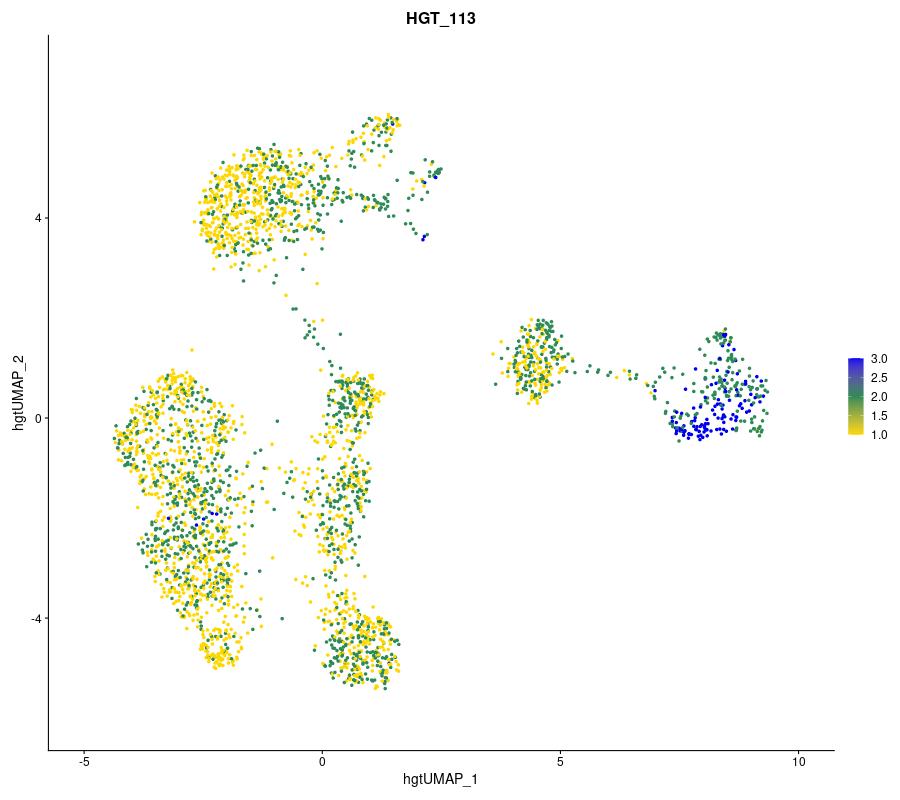

Supplement: Supplementary file 5 — Supplementary Data 3 [file 41467_2023_36559_MOESM5_ESM.zip › all128embedding/113 .jpg]

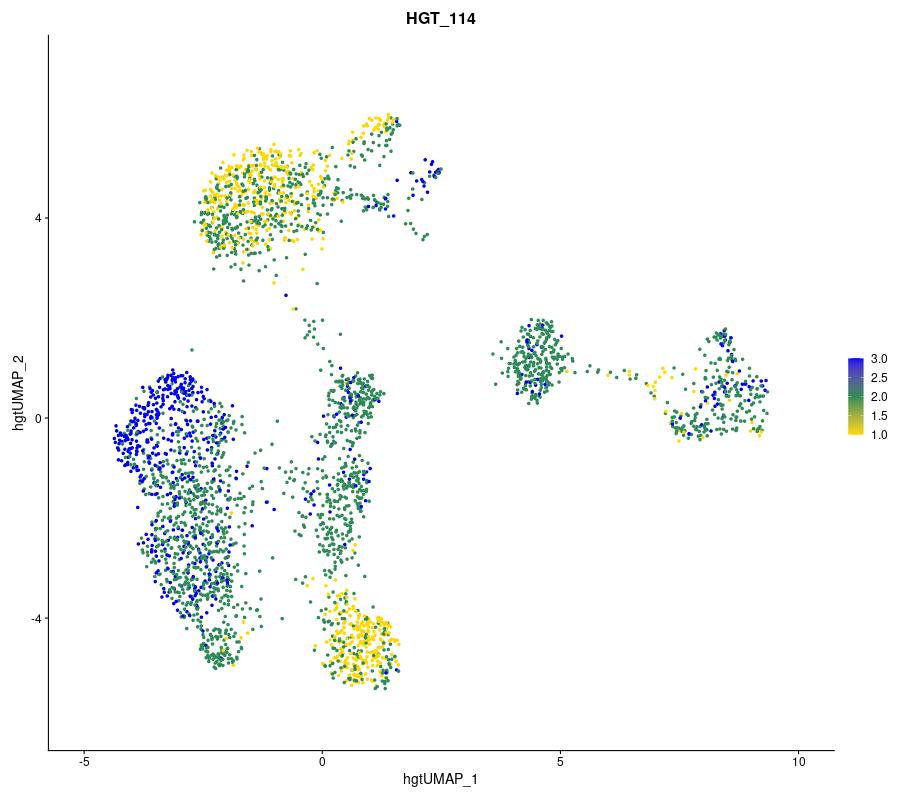

Supplement: Supplementary file 5 — Supplementary Data 3 [file 41467_2023_36559_MOESM5_ESM.zip › all128embedding/114 .jpg]

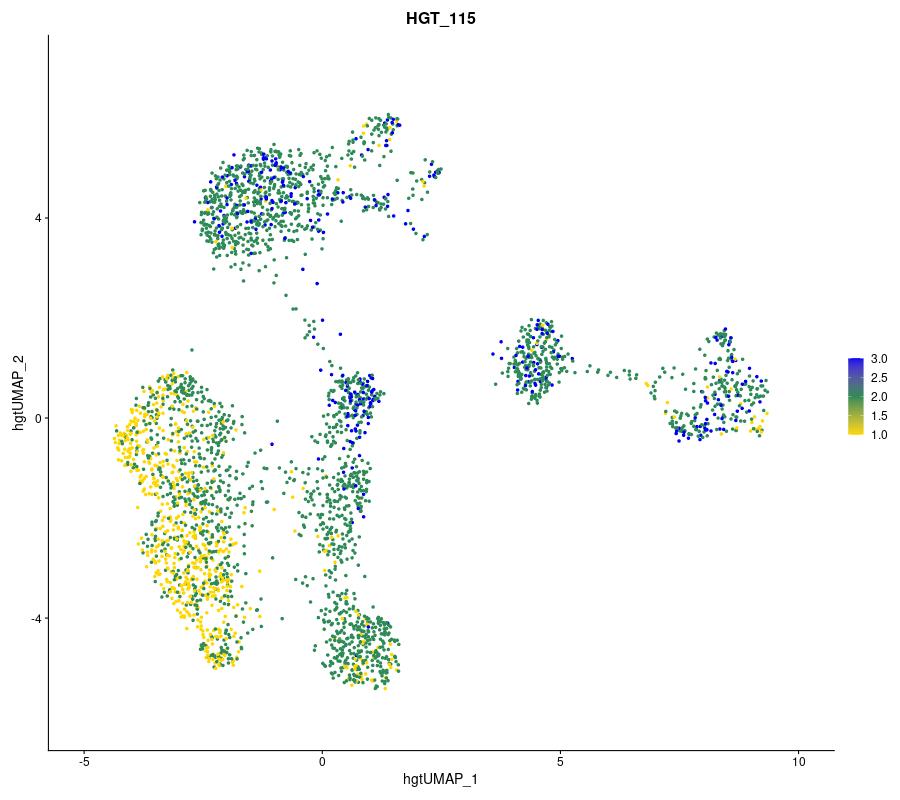

Supplement: Supplementary file 5 — Supplementary Data 3 [file 41467_2023_36559_MOESM5_ESM.zip › all128embedding/115 .jpg]

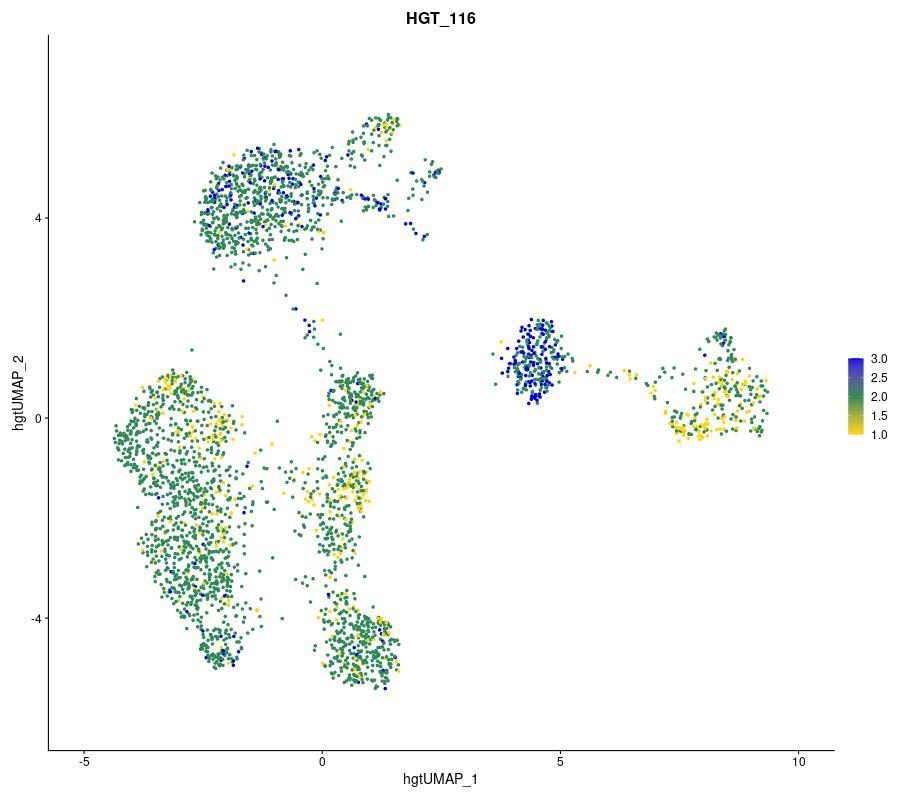

Supplement: Supplementary file 5 — Supplementary Data 3 [file 41467_2023_36559_MOESM5_ESM.zip › all128embedding/116 .jpg]

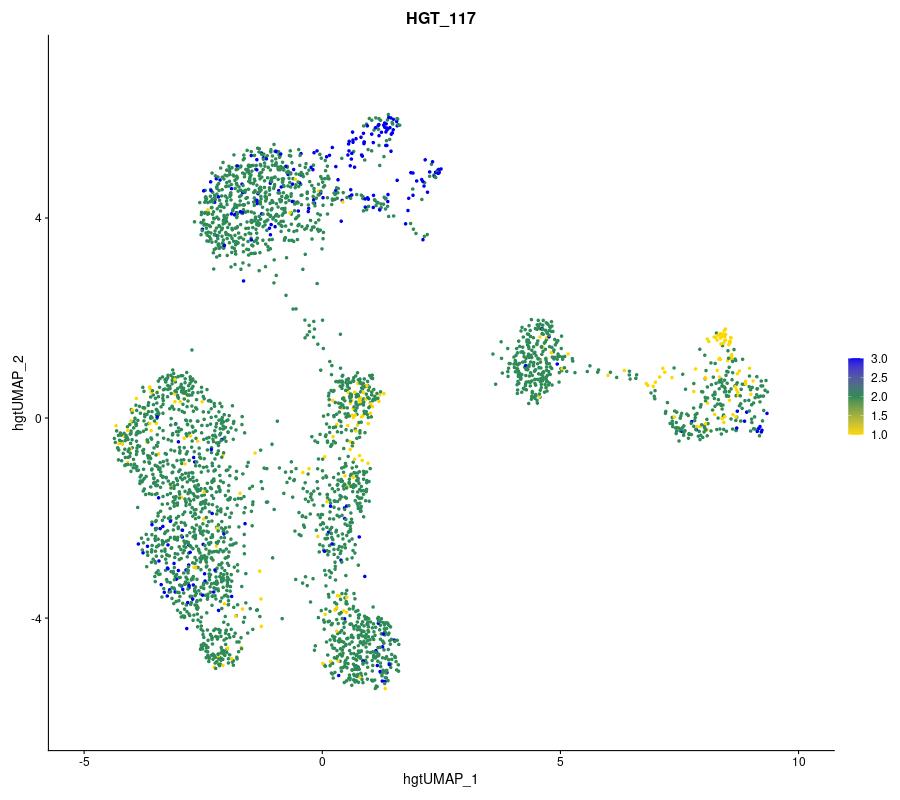

Supplement: Supplementary file 5 — Supplementary Data 3 [file 41467_2023_36559_MOESM5_ESM.zip › all128embedding/117 .jpg]

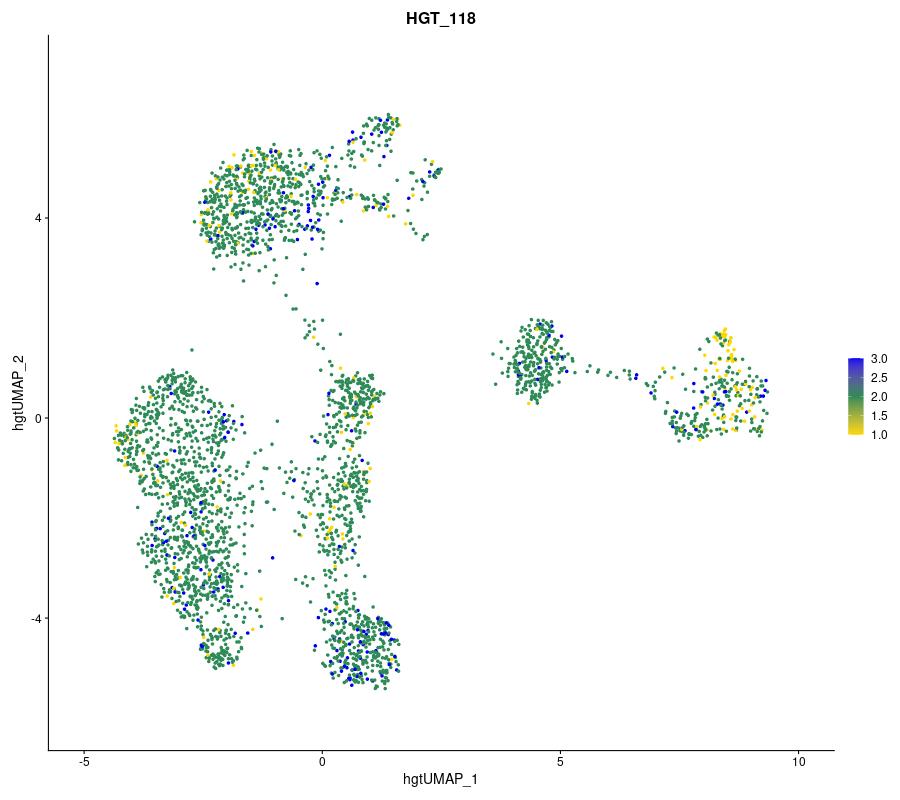

Supplement: Supplementary file 5 — Supplementary Data 3 [file 41467_2023_36559_MOESM5_ESM.zip › all128embedding/118 .jpg]

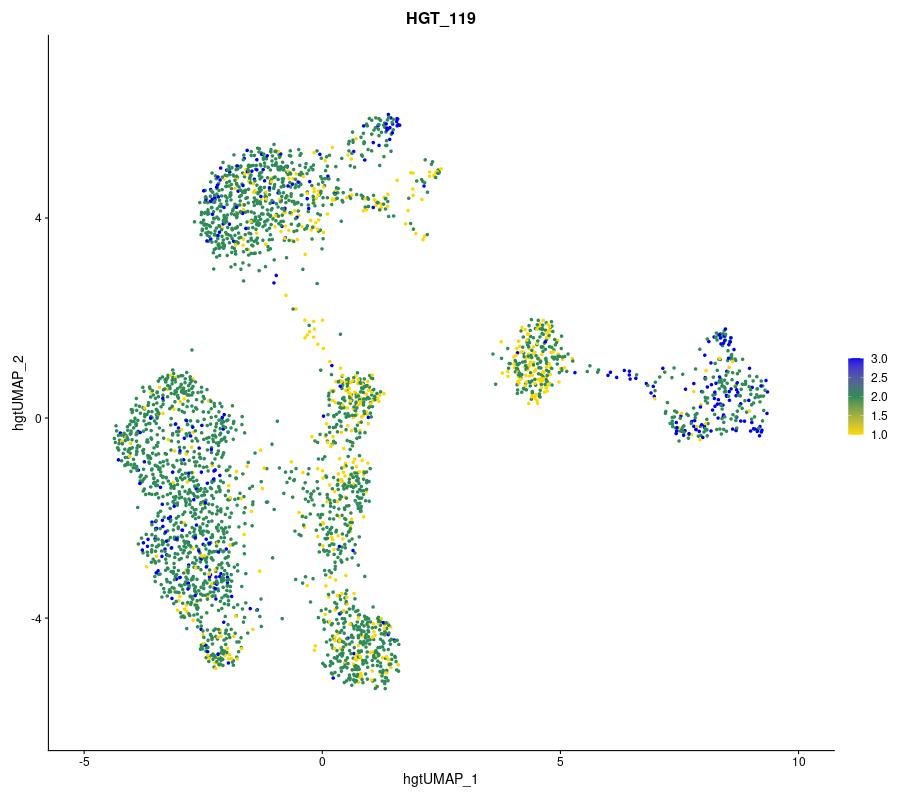

Supplement: Supplementary file 5 — Supplementary Data 3 [file 41467_2023_36559_MOESM5_ESM.zip › all128embedding/119 .jpg]

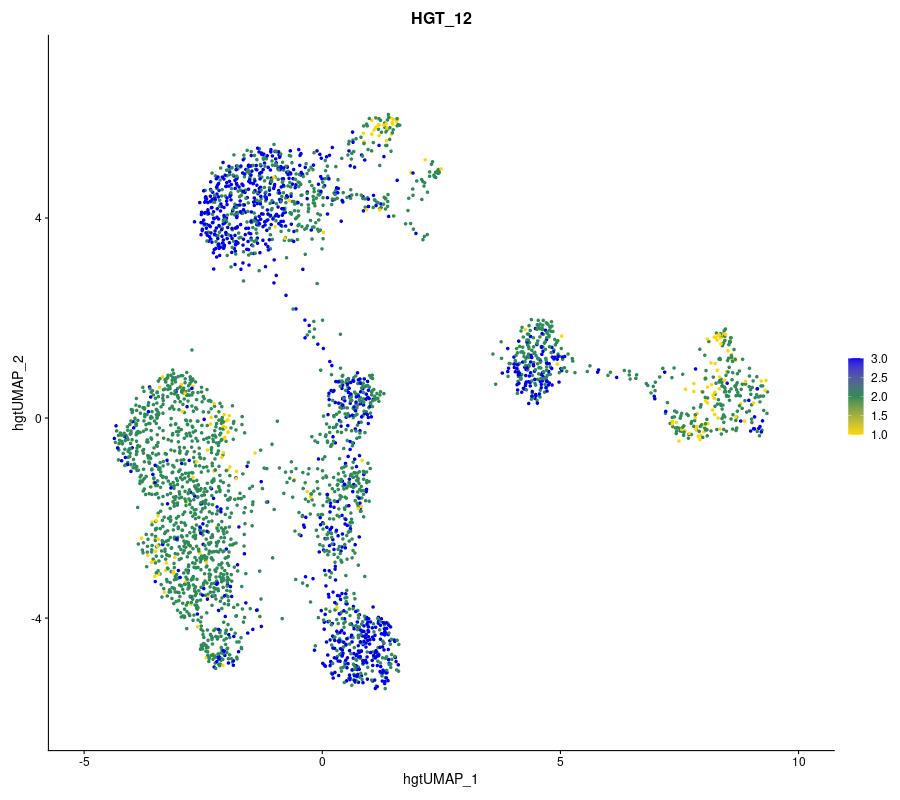

Supplement: Supplementary file 5 — Supplementary Data 3 [file 41467_2023_36559_MOESM5_ESM.zip › all128embedding/12 .jpg]

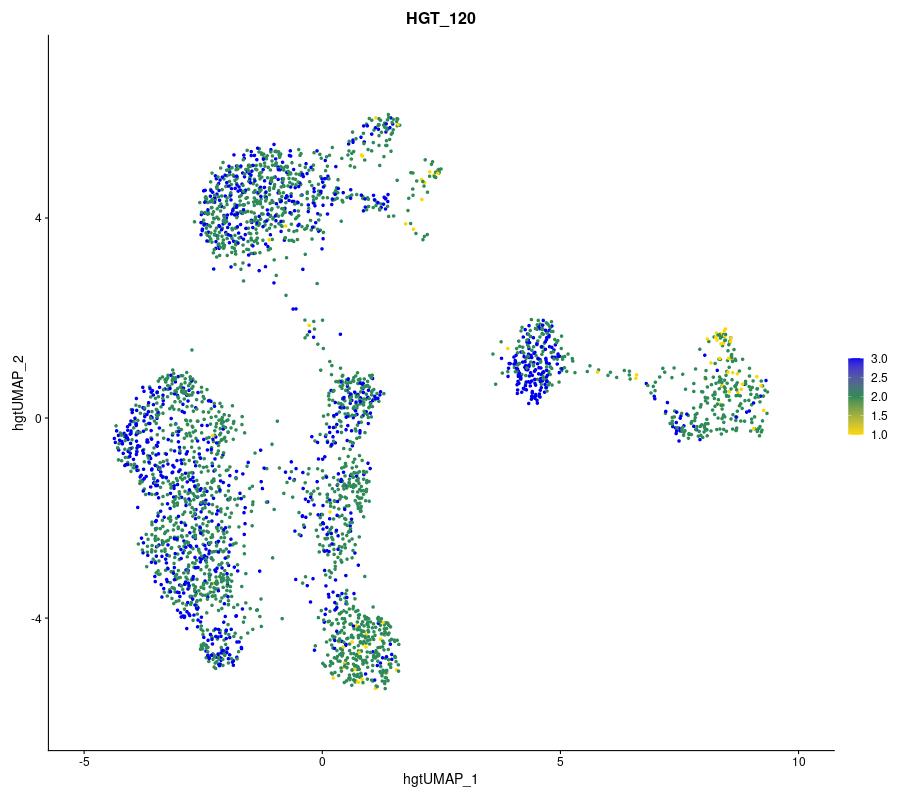

Supplement: Supplementary file 5 — Supplementary Data 3 [file 41467_2023_36559_MOESM5_ESM.zip › all128embedding/120 .jpg]

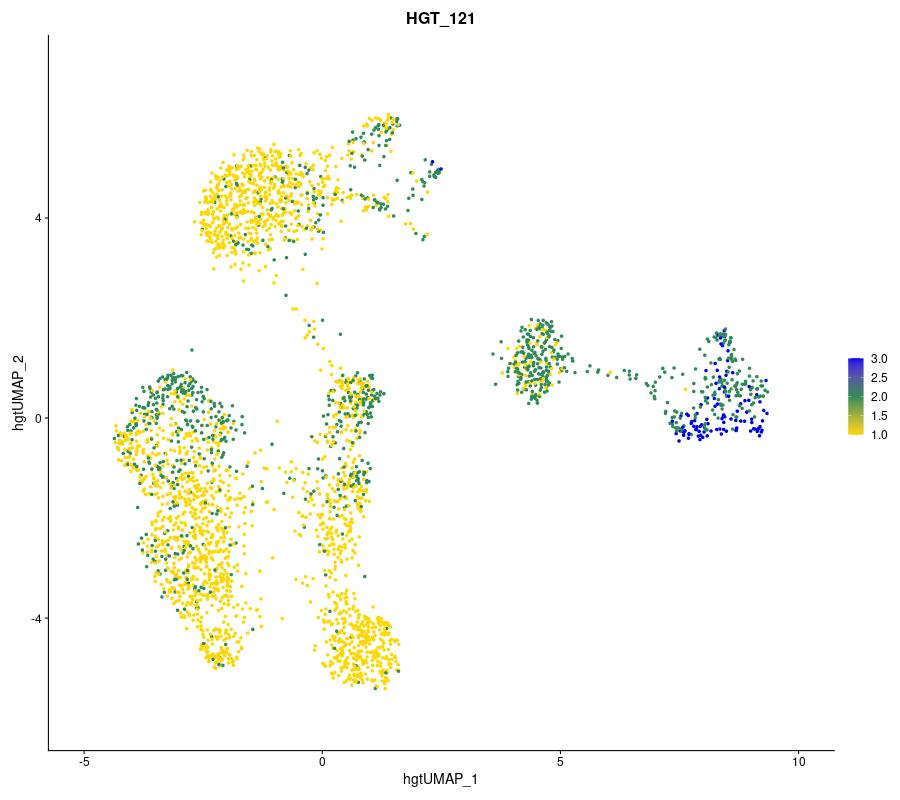

Supplement: Supplementary file 5 — Supplementary Data 3 [file 41467_2023_36559_MOESM5_ESM.zip › all128embedding/121 .jpg]

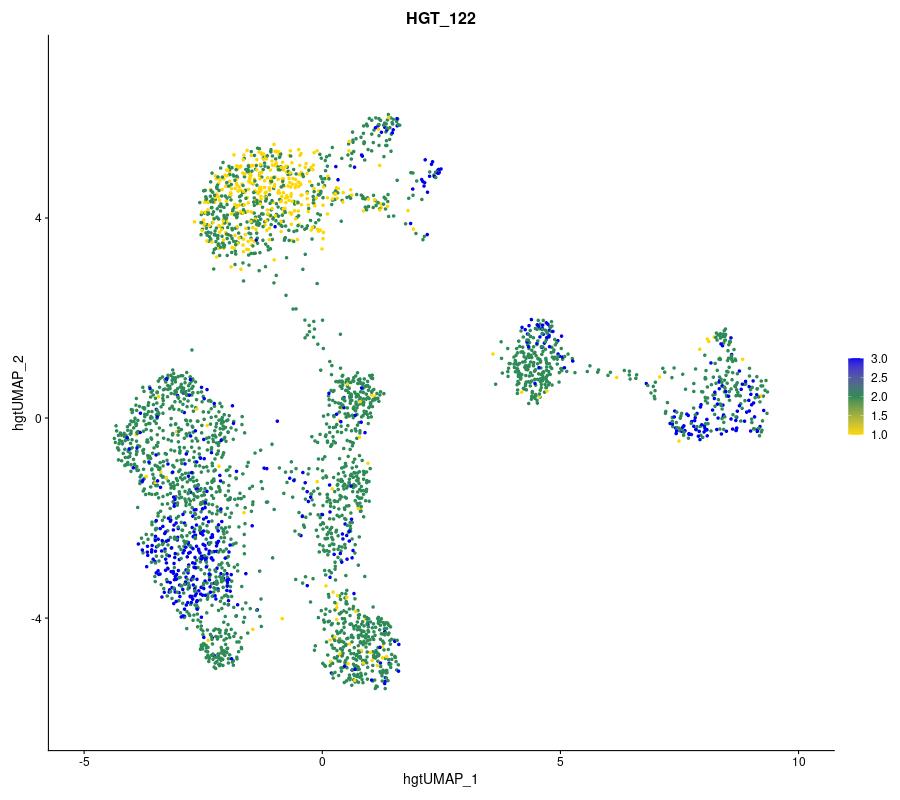

Supplement: Supplementary file 5 — Supplementary Data 3 [file 41467_2023_36559_MOESM5_ESM.zip › all128embedding/122 .jpg]

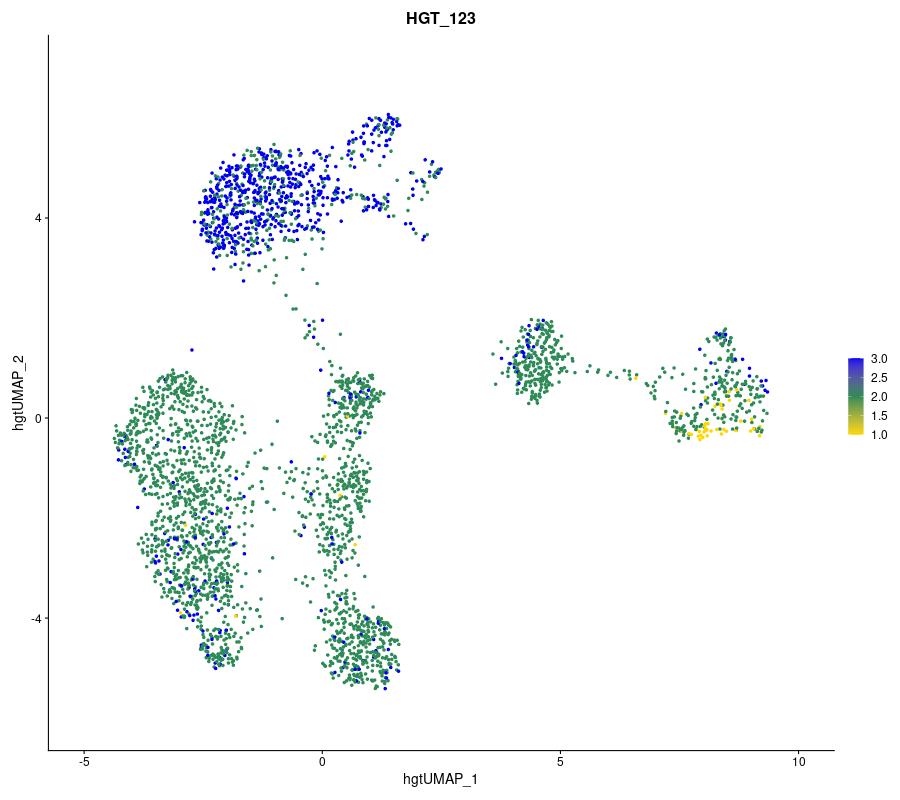

Supplement: Supplementary file 5 — Supplementary Data 3 [file 41467_2023_36559_MOESM5_ESM.zip › all128embedding/123 .jpg]

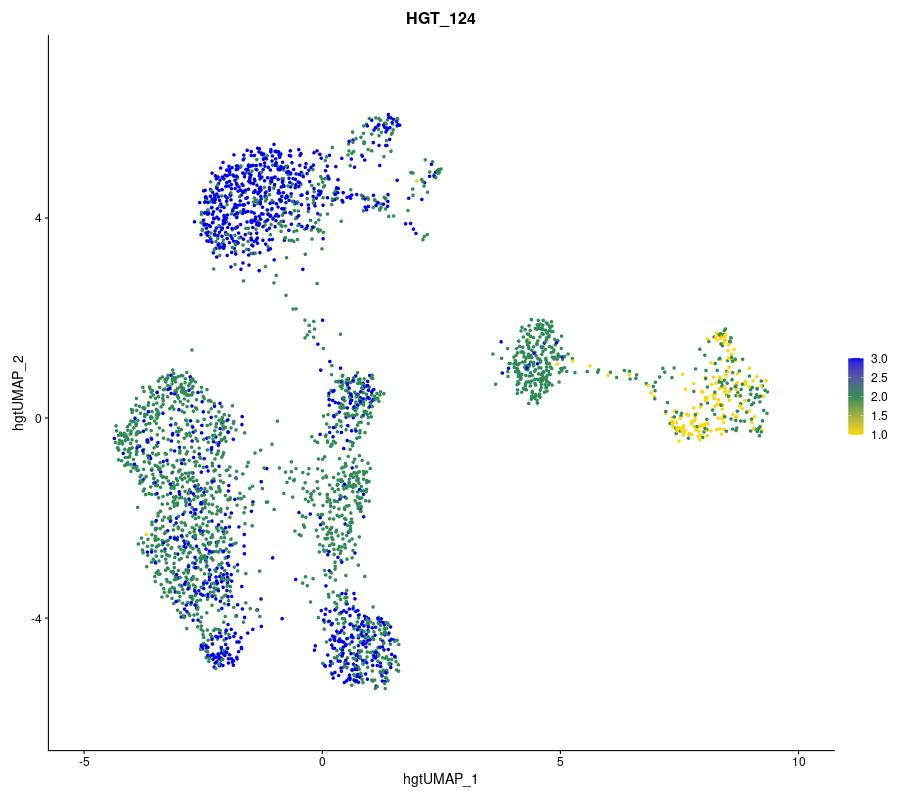

Supplement: Supplementary file 5 — Supplementary Data 3 [file 41467_2023_36559_MOESM5_ESM.zip › all128embedding/124 .jpg]

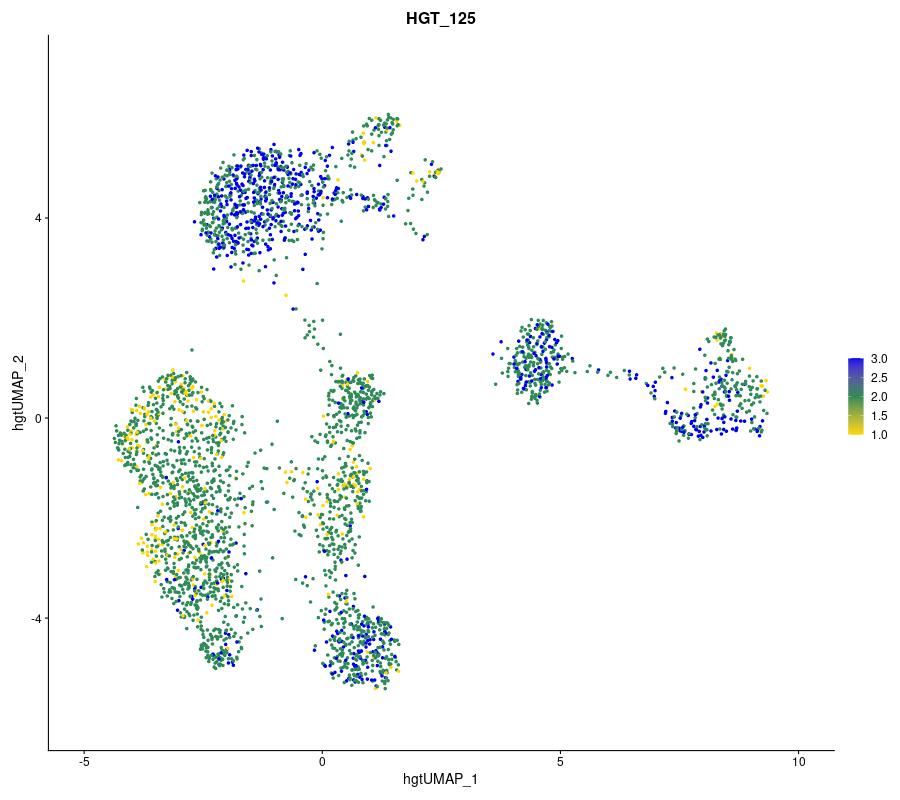

Supplement: Supplementary file 5 — Supplementary Data 3 [file 41467_2023_36559_MOESM5_ESM.zip › all128embedding/125 .jpg]

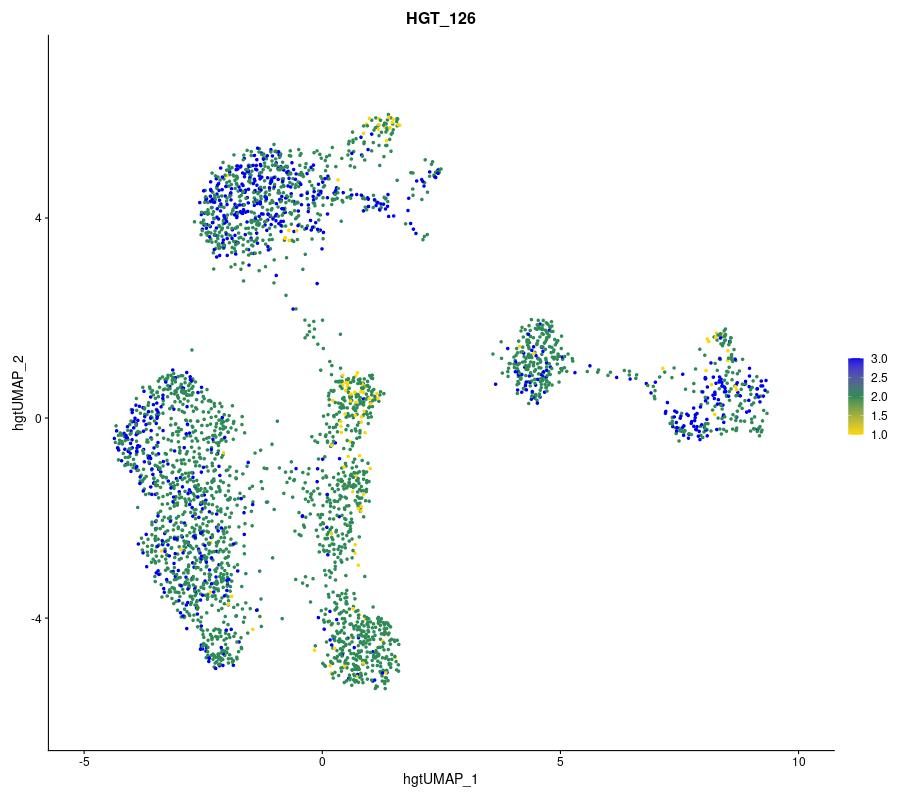

Supplement: Supplementary file 5 — Supplementary Data 3 [file 41467_2023_36559_MOESM5_ESM.zip › all128embedding/126 .jpg]

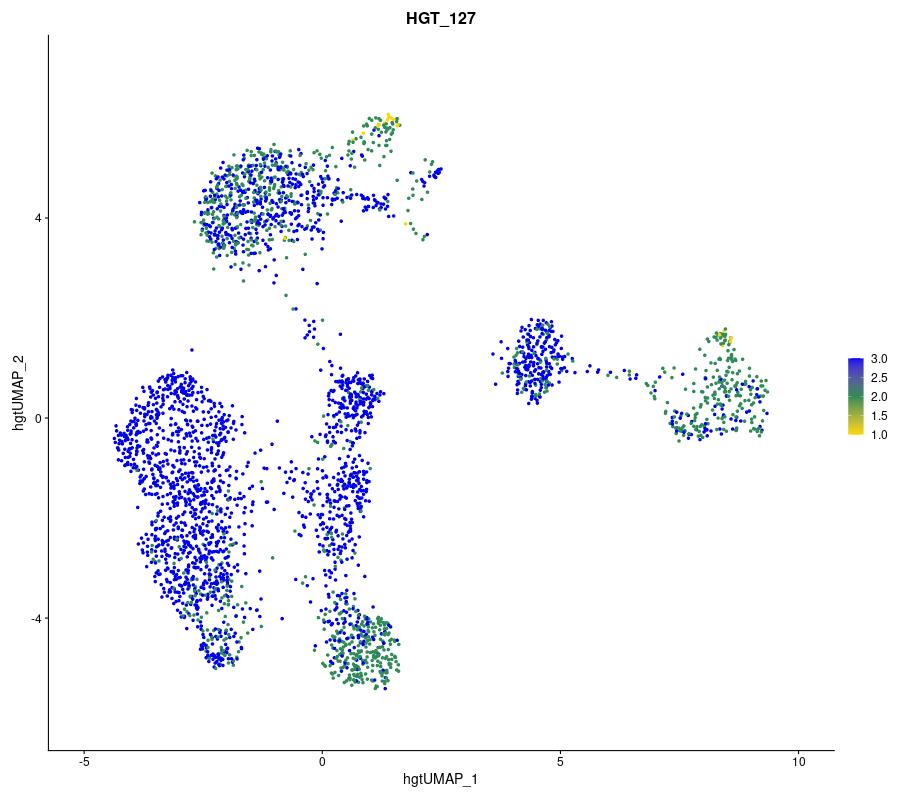

Supplement: Supplementary file 5 — Supplementary Data 3 [file 41467_2023_36559_MOESM5_ESM.zip › all128embedding/127 .jpg]

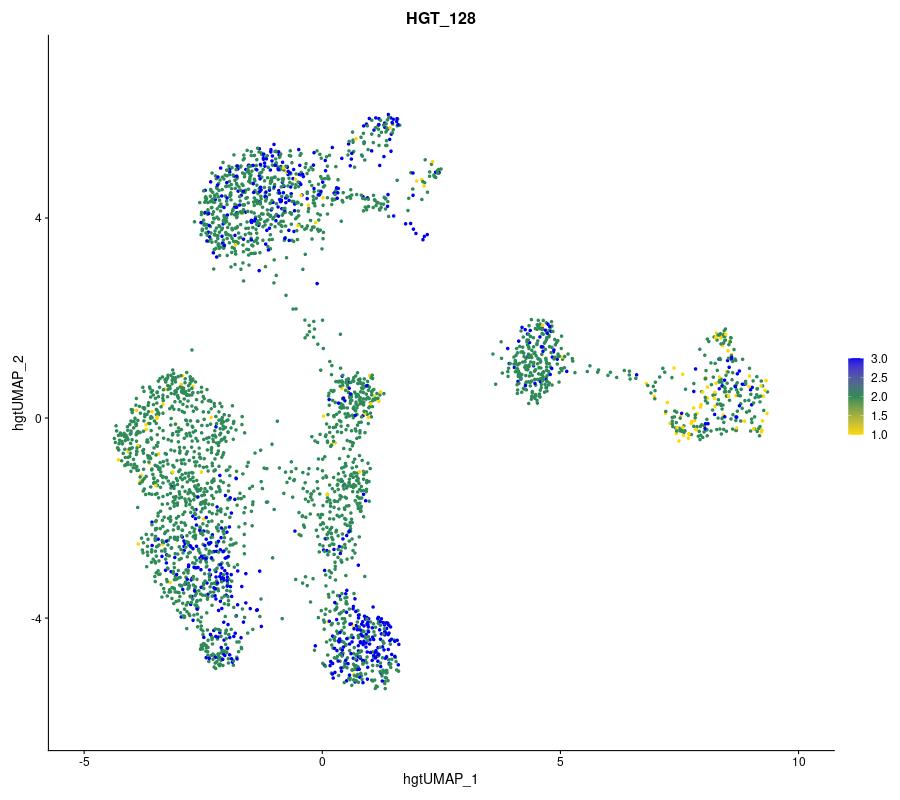

Supplement: Supplementary file 5 — Supplementary Data 3 [file 41467_2023_36559_MOESM5_ESM.zip › all128embedding/128 .jpg]

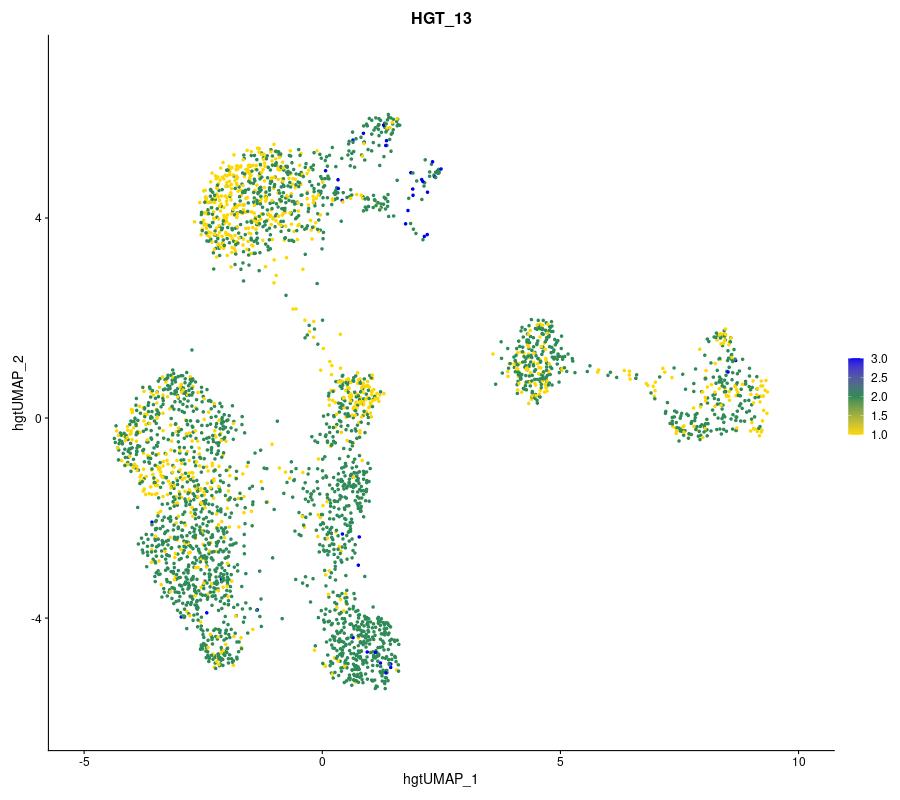

Supplement: Supplementary file 5 — Supplementary Data 3 [file 41467_2023_36559_MOESM5_ESM.zip › all128embedding/13 .jpg]

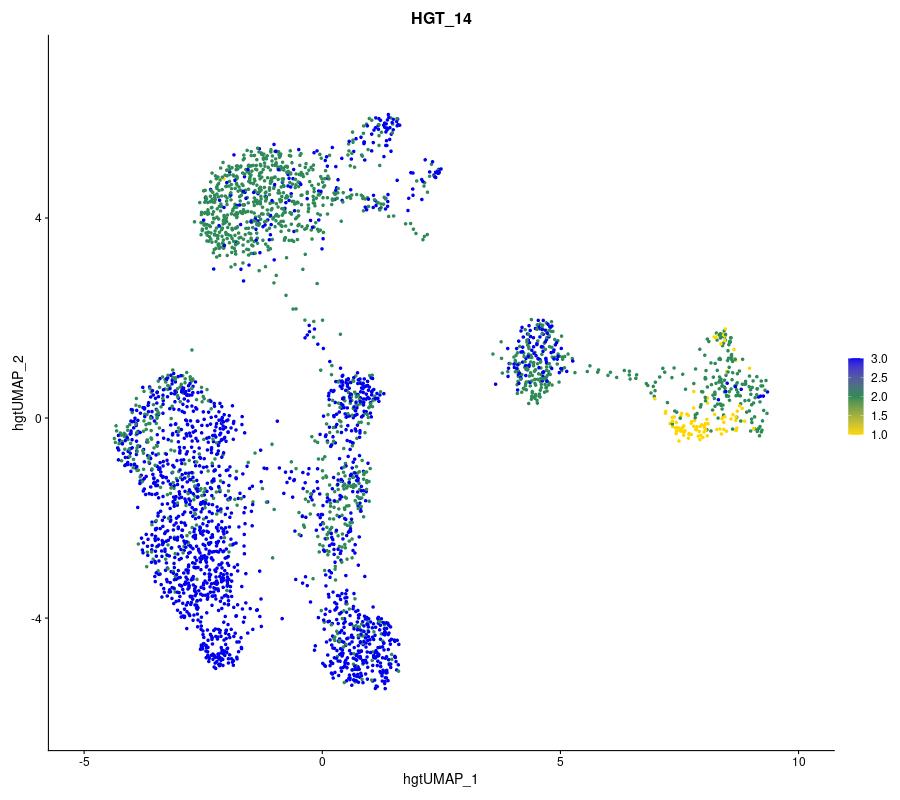

Supplement: Supplementary file 5 — Supplementary Data 3 [file 41467_2023_36559_MOESM5_ESM.zip › all128embedding/14 .jpg]

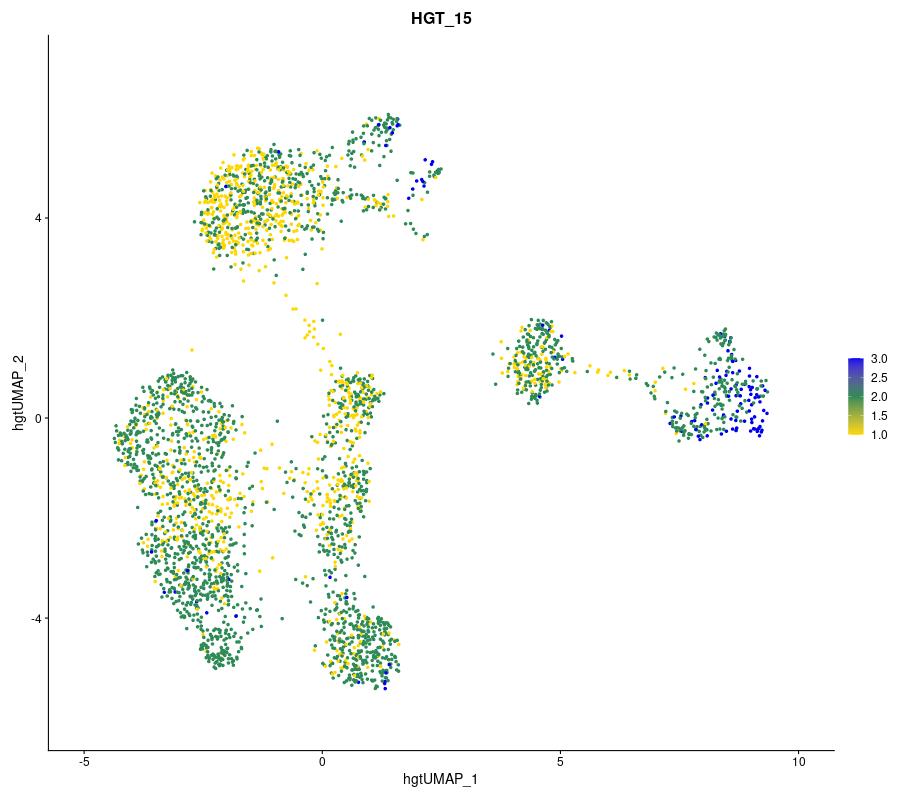

Supplement: Supplementary file 5 — Supplementary Data 3 [file 41467_2023_36559_MOESM5_ESM.zip › all128embedding/15 .jpg]

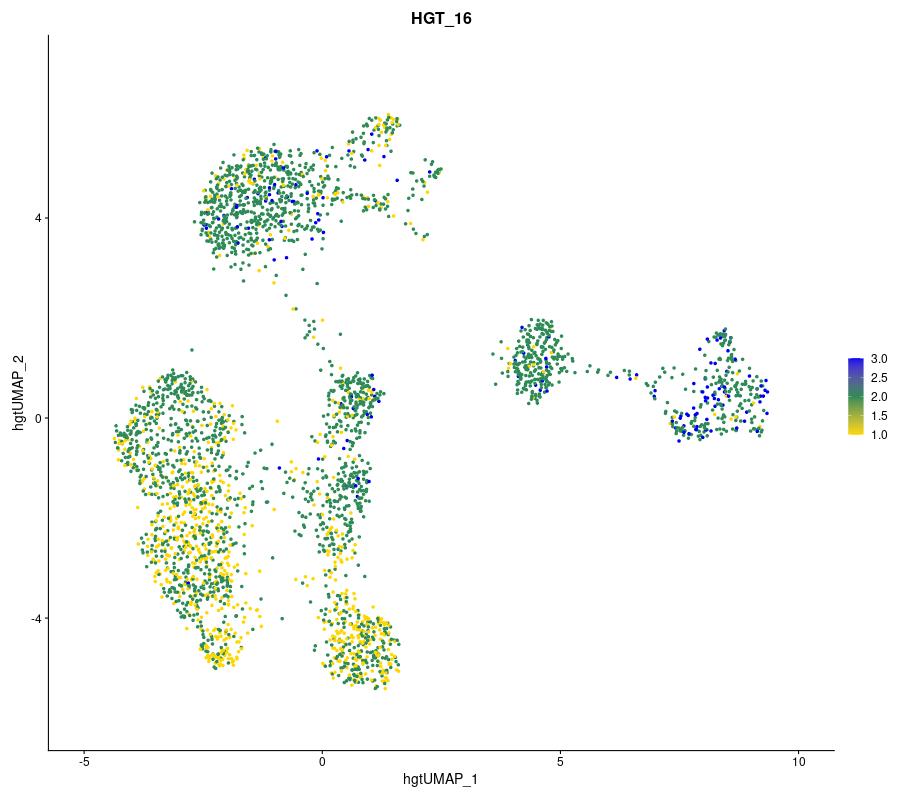

Supplement: Supplementary file 5 — Supplementary Data 3 [file 41467_2023_36559_MOESM5_ESM.zip › all128embedding/16 .jpg]

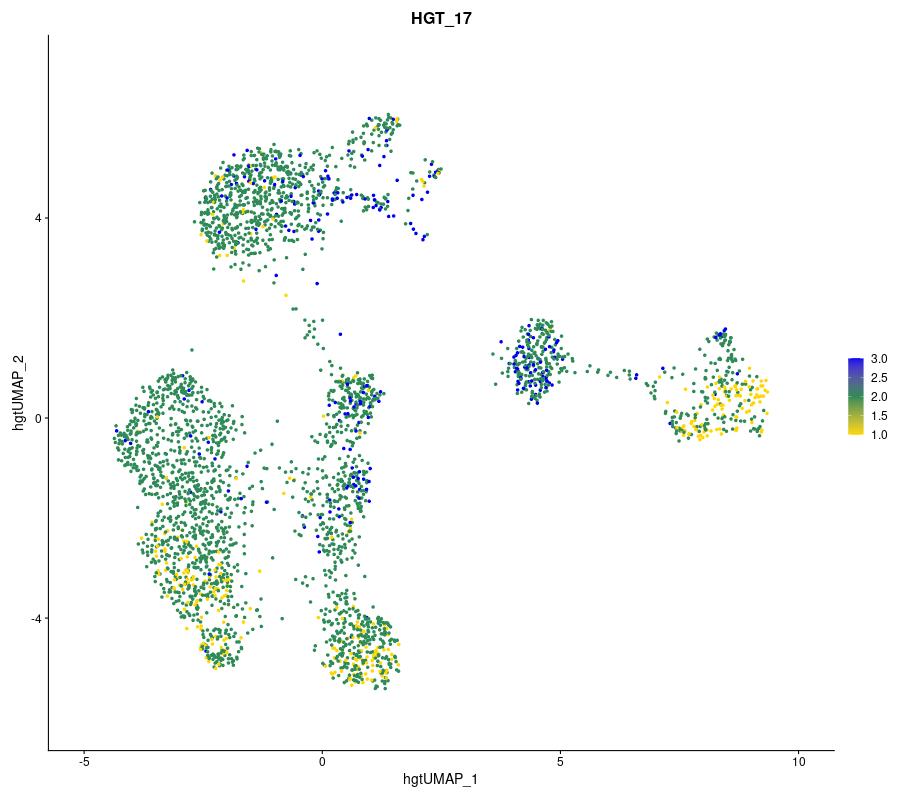

Supplement: Supplementary file 5 — Supplementary Data 3 [file 41467_2023_36559_MOESM5_ESM.zip › all128embedding/17 .jpg]

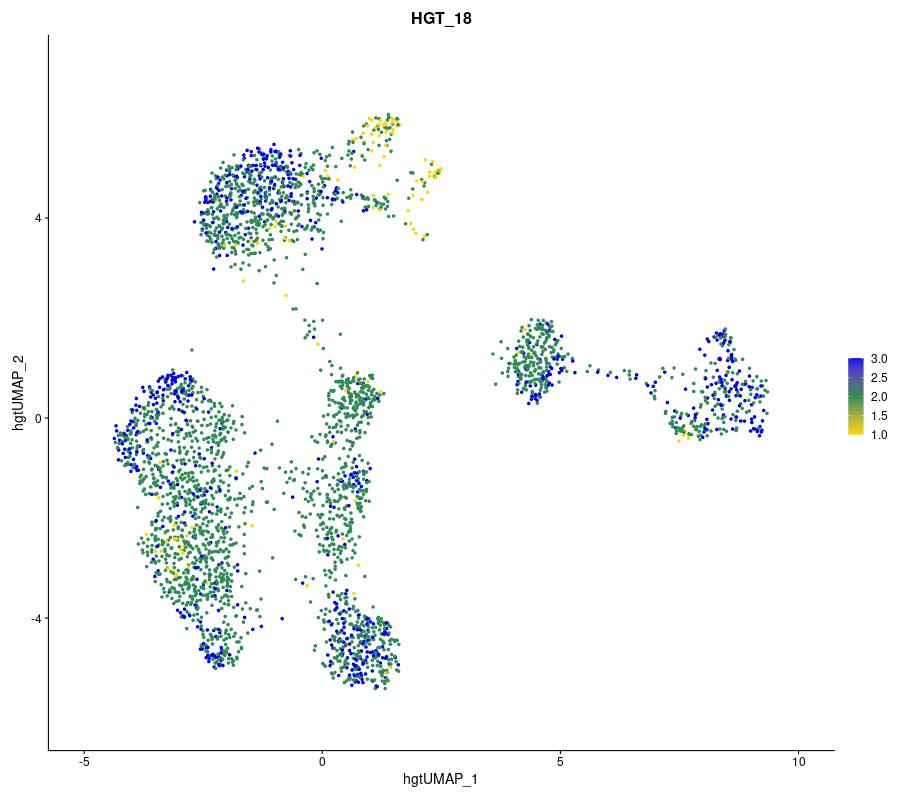

Supplement: Supplementary file 5 — Supplementary Data 3 [file 41467_2023_36559_MOESM5_ESM.zip › all128embedding/18 .jpg]

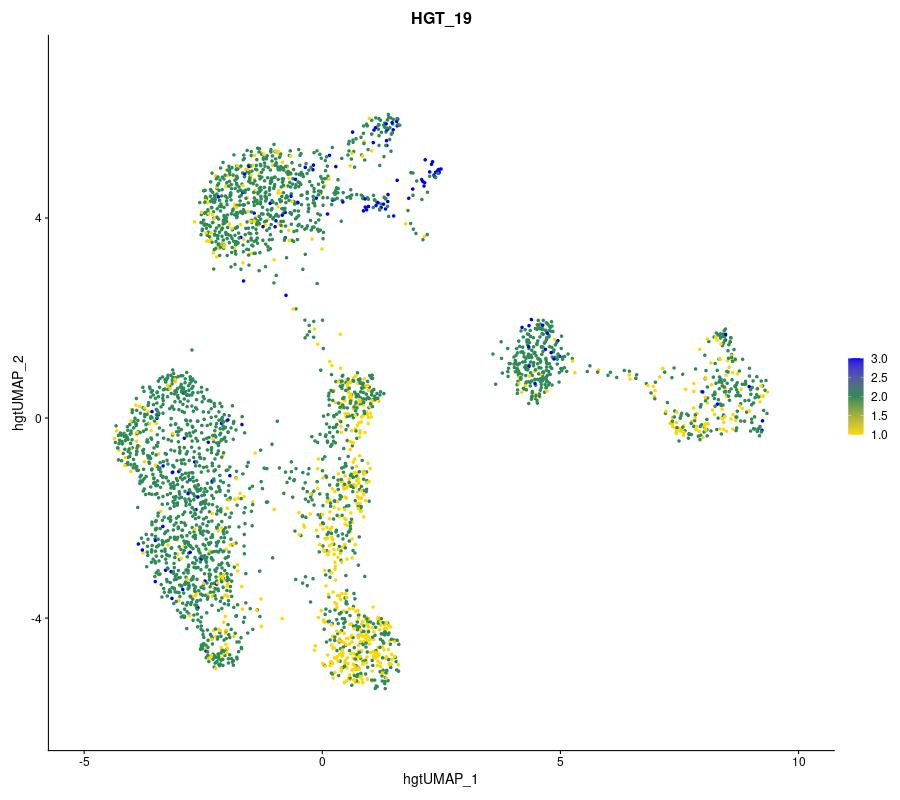

Supplement: Supplementary file 5 — Supplementary Data 3 [file 41467_2023_36559_MOESM5_ESM.zip › all128embedding/19 .jpg]

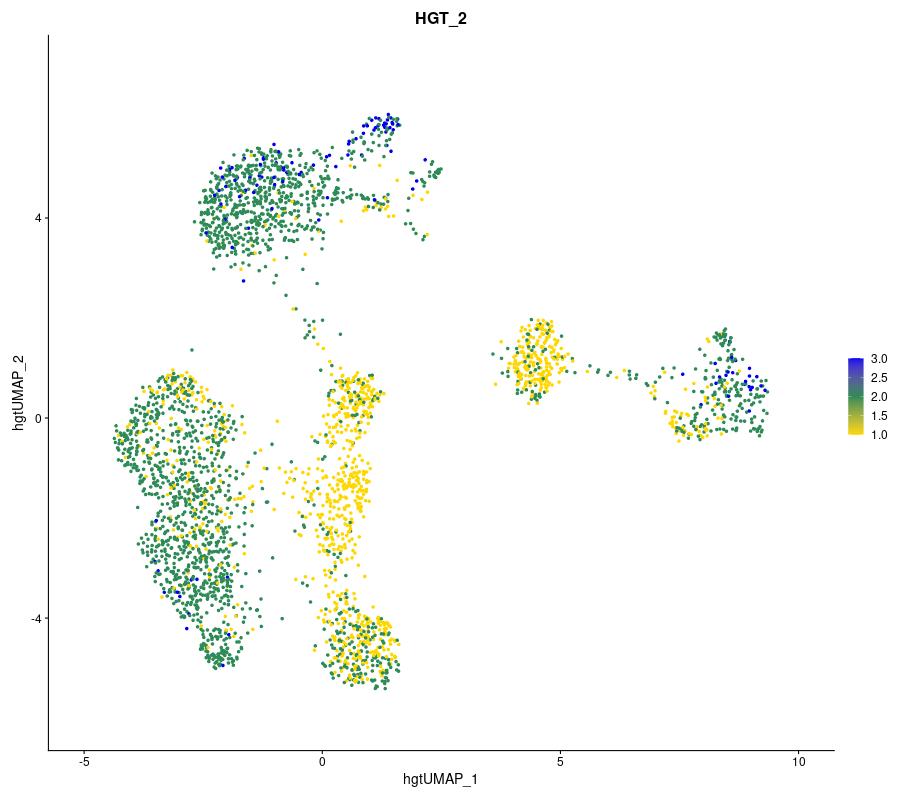

Supplement: Supplementary file 5 — Supplementary Data 3 [file 41467_2023_36559_MOESM5_ESM.zip › all128embedding/2 .jpg]

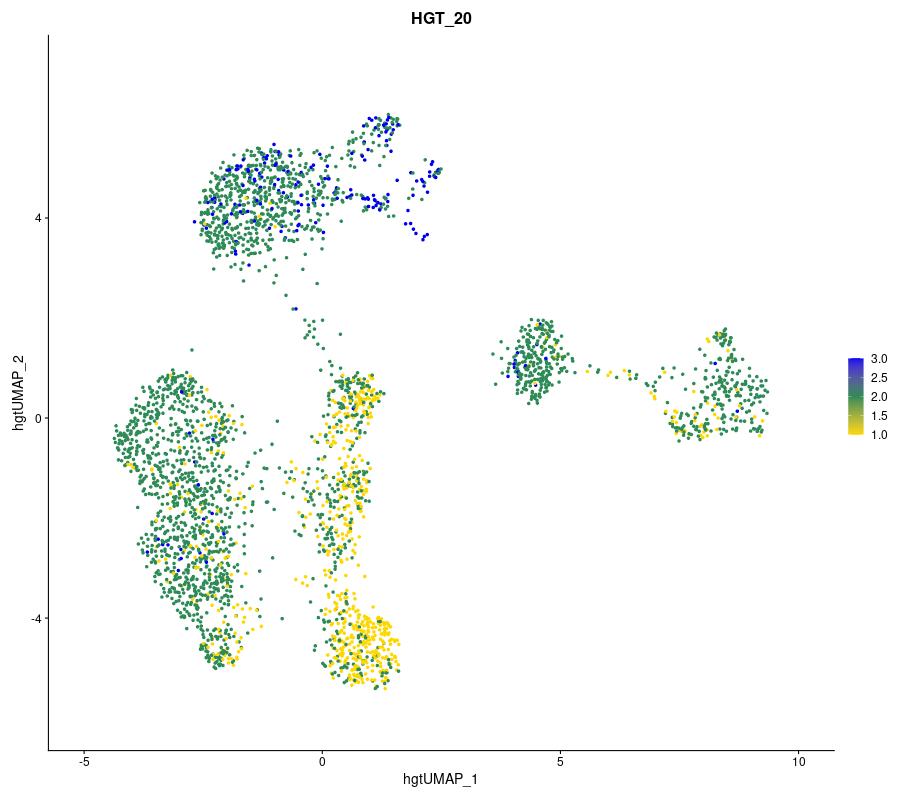

Supplement: Supplementary file 5 — Supplementary Data 3 [file 41467_2023_36559_MOESM5_ESM.zip › all128embedding/20 .jpg]

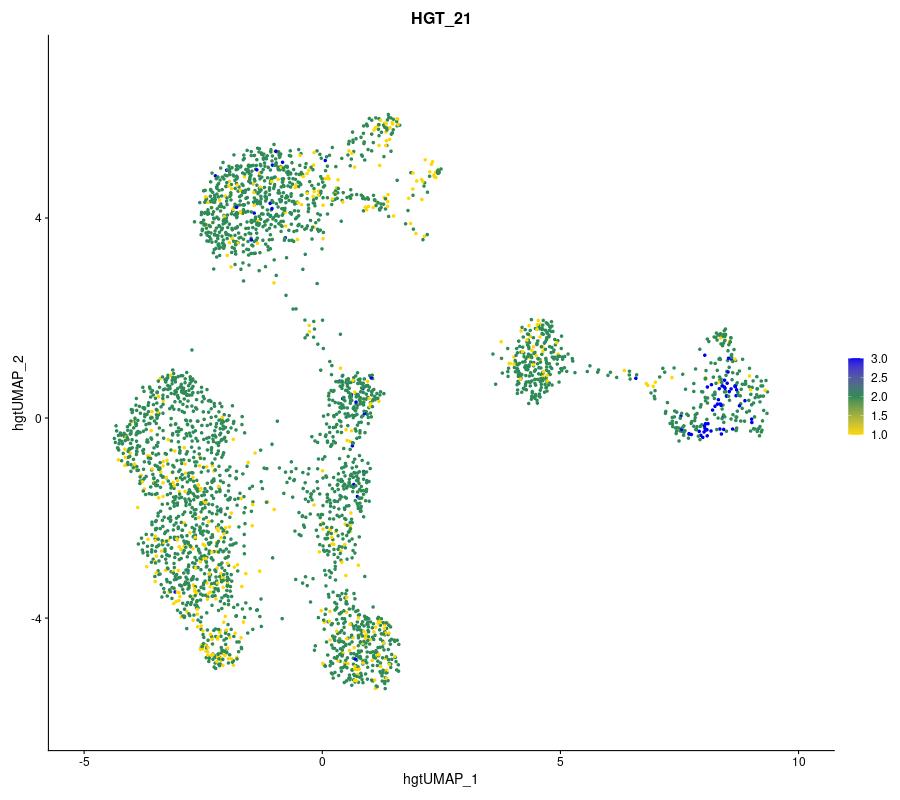

Supplement: Supplementary file 5 — Supplementary Data 3 [file 41467_2023_36559_MOESM5_ESM.zip › all128embedding/21 .jpg]

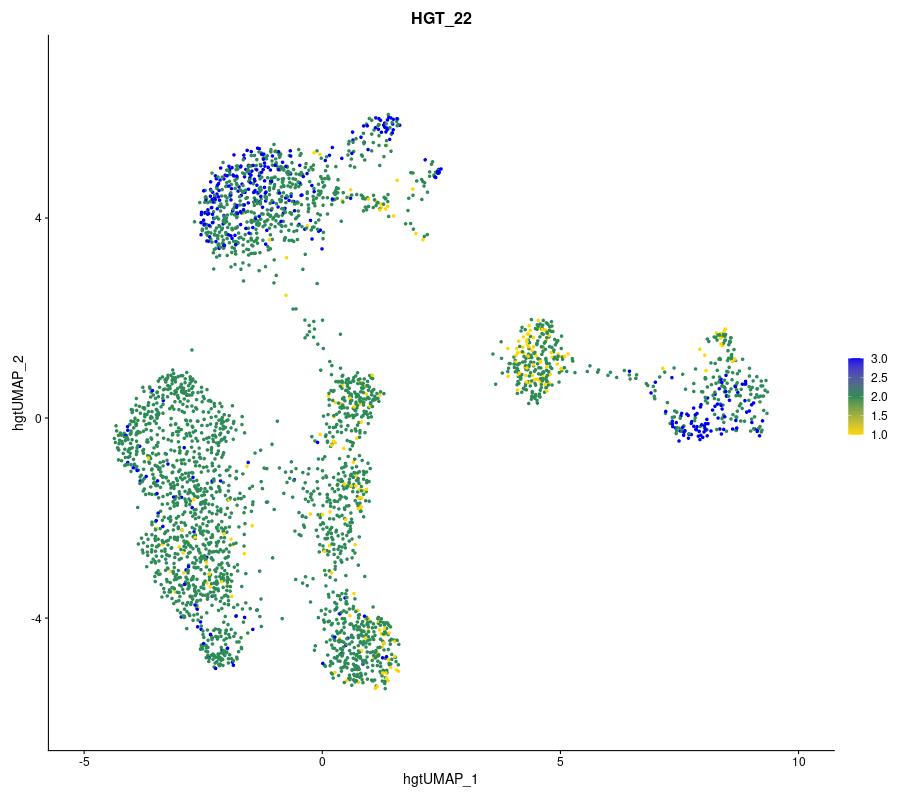

Supplement: Supplementary file 5 — Supplementary Data 3 [file 41467_2023_36559_MOESM5_ESM.zip › all128embedding/22 .jpg]

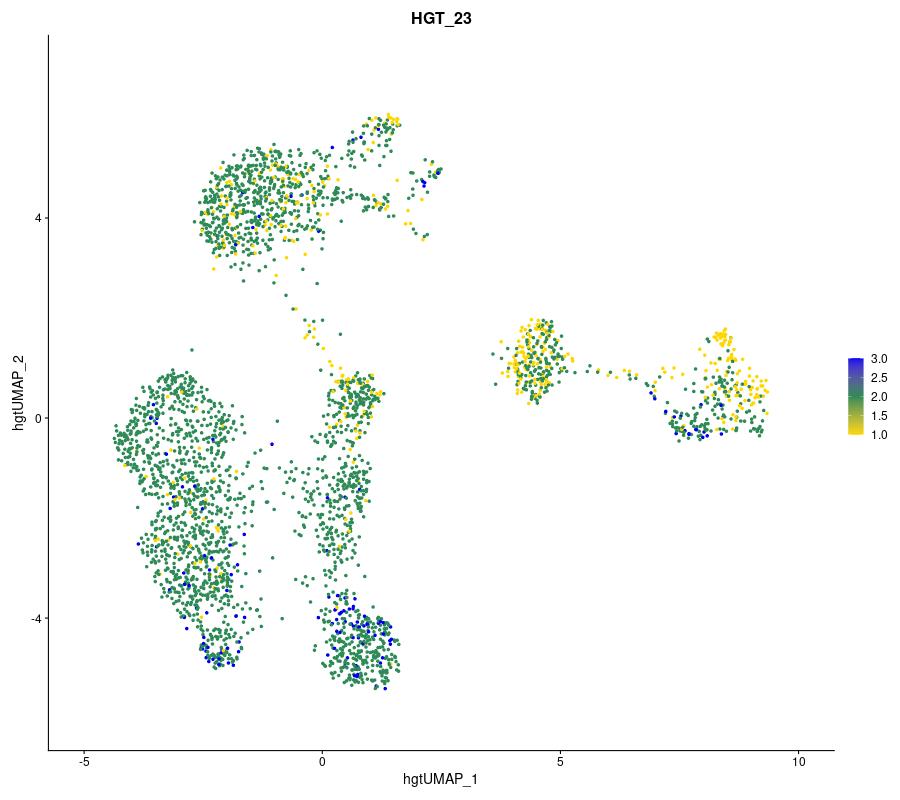

Supplement: Supplementary file 5 — Supplementary Data 3 [file 41467_2023_36559_MOESM5_ESM.zip › all128embedding/23 .jpg]

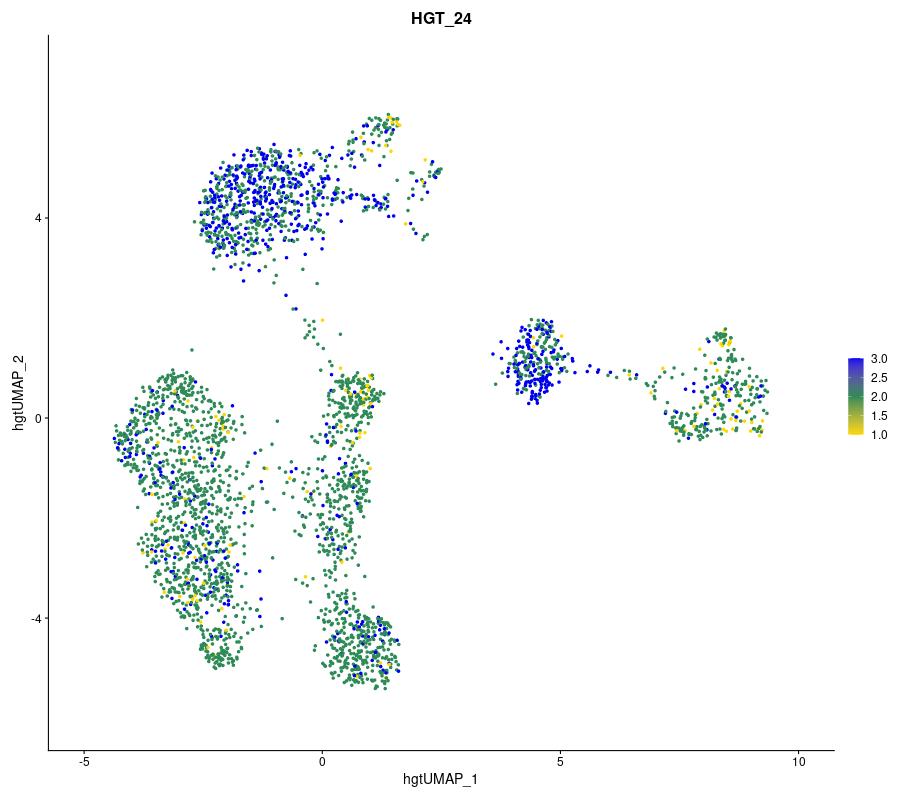

Supplement: Supplementary file 5 — Supplementary Data 3 [file 41467_2023_36559_MOESM5_ESM.zip › all128embedding/24 .jpg]

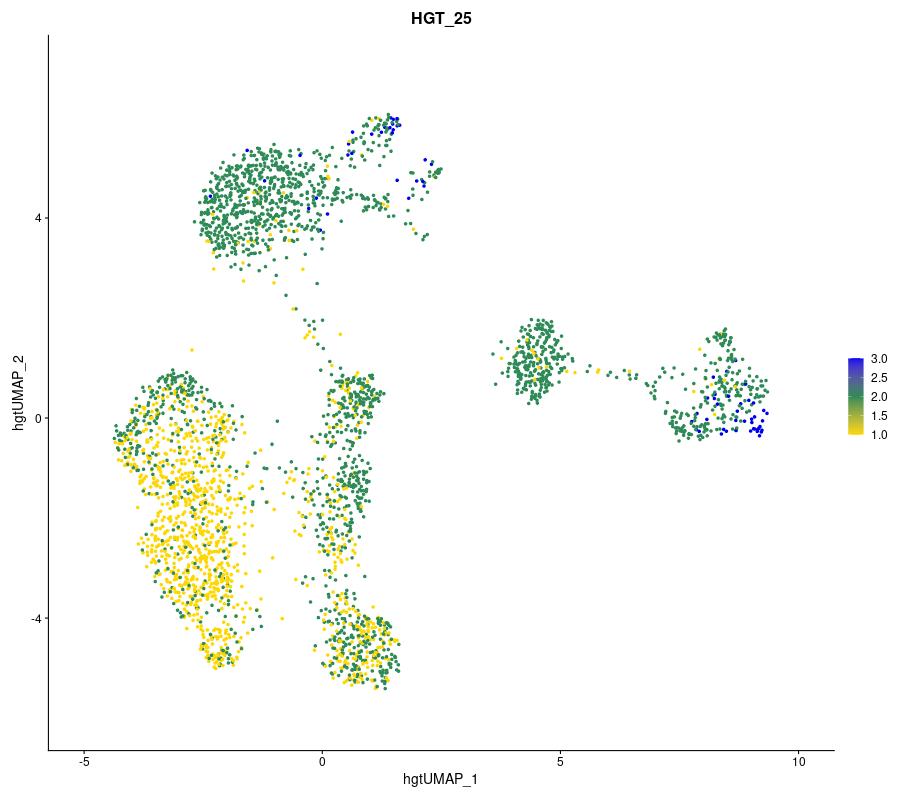

Supplement: Supplementary file 5 — Supplementary Data 3 [file 41467_2023_36559_MOESM5_ESM.zip › all128embedding/25 .jpg]

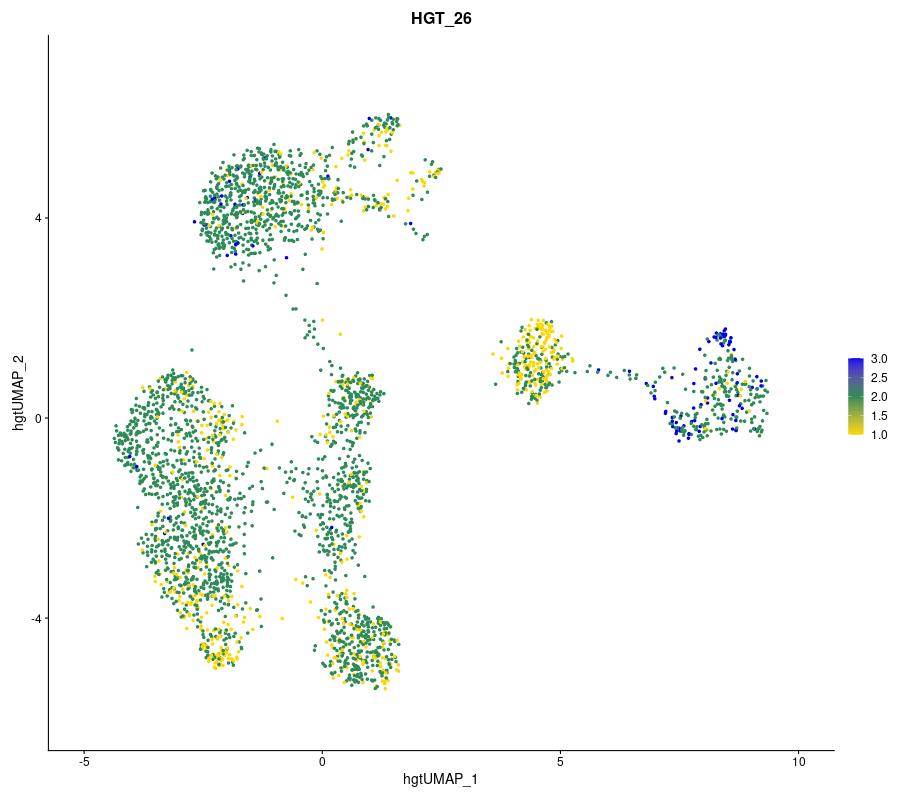

Supplement: Supplementary file 5 — Supplementary Data 3 [file 41467_2023_36559_MOESM5_ESM.zip › all128embedding/26 .jpg]

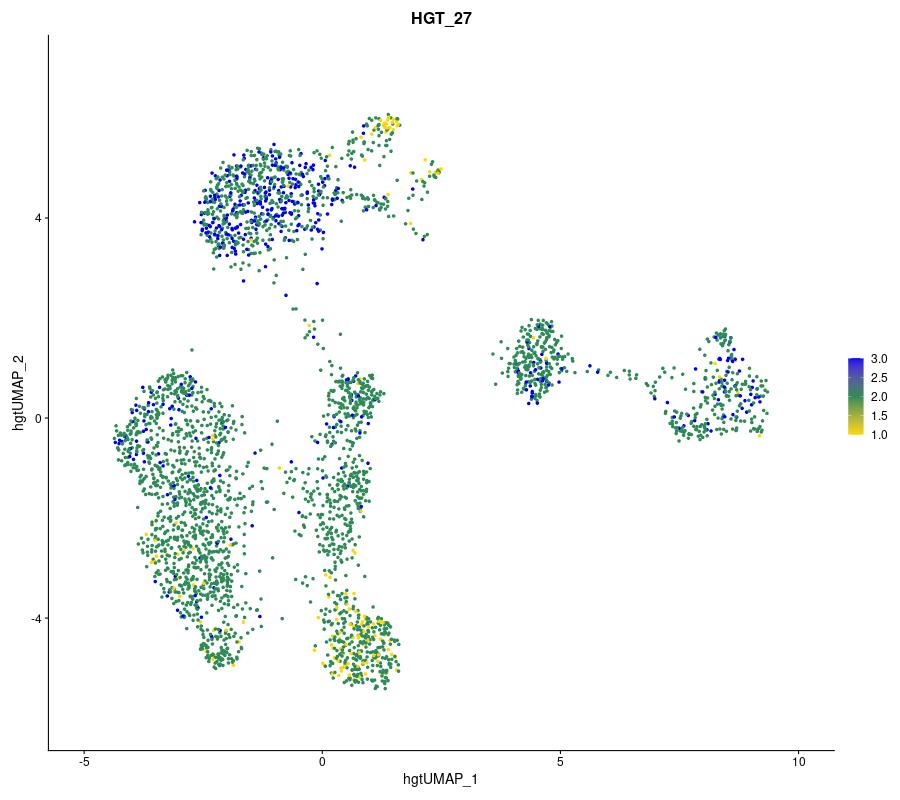

Supplement: Supplementary file 5 — Supplementary Data 3 [file 41467_2023_36559_MOESM5_ESM.zip › all128embedding/27 .jpg]

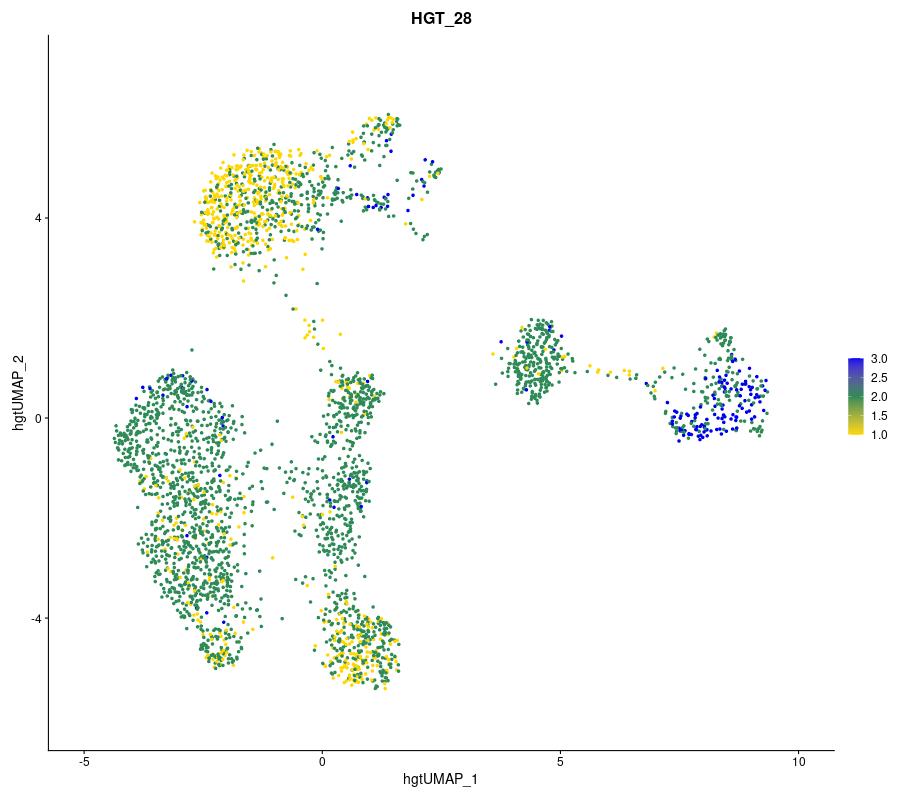

Supplement: Supplementary file 5 — Supplementary Data 3 [file 41467_2023_36559_MOESM5_ESM.zip › all128embedding/28 .jpg]

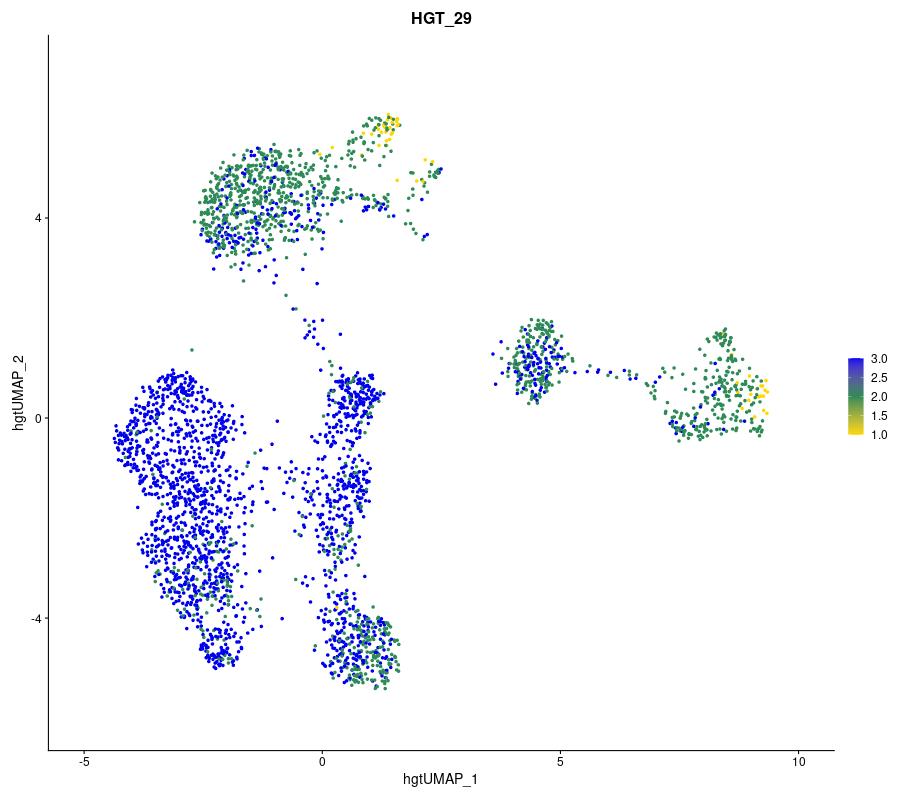

Supplement: Supplementary file 5 — Supplementary Data 3 [file 41467_2023_36559_MOESM5_ESM.zip › all128embedding/29 .jpg]

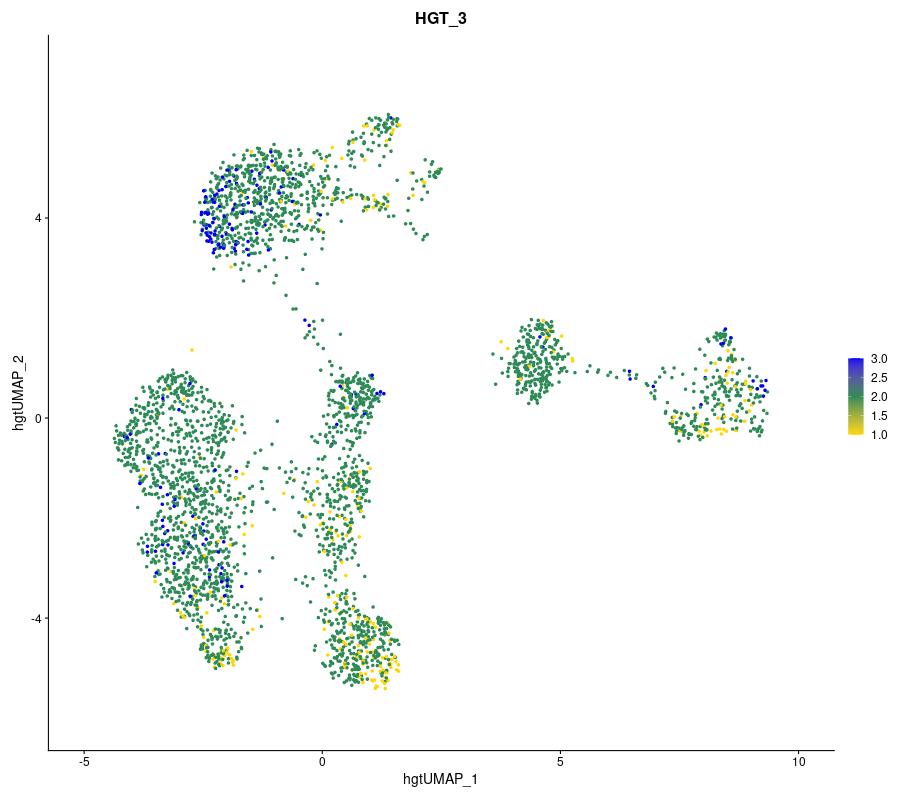

Supplement: Supplementary file 5 — Supplementary Data 3 [file 41467_2023_36559_MOESM5_ESM.zip › all128embedding/3 .jpg]

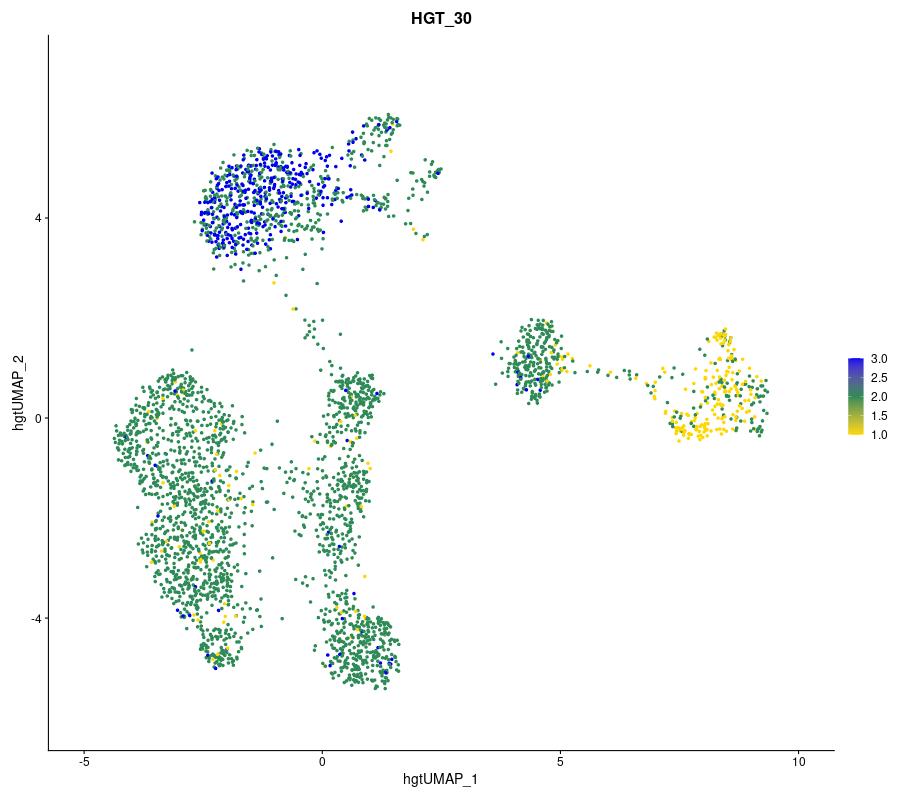

Supplement: Supplementary file 5 — Supplementary Data 3 [file 41467_2023_36559_MOESM5_ESM.zip › all128embedding/30 .jpg]

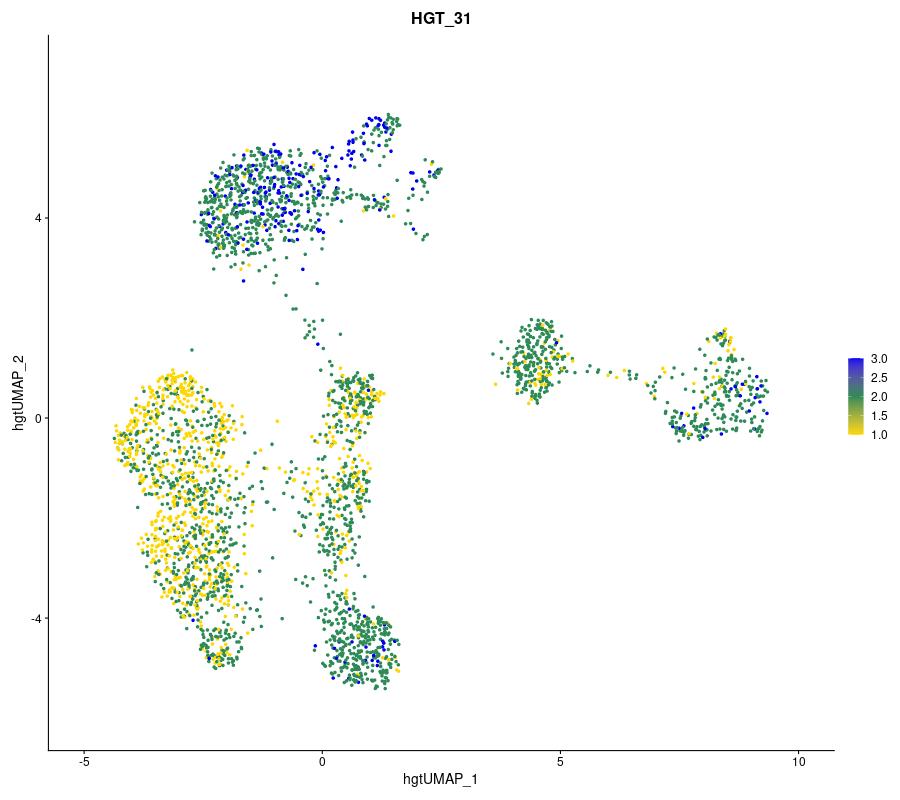

Supplement: Supplementary file 5 — Supplementary Data 3 [file 41467_2023_36559_MOESM5_ESM.zip › all128embedding/31 .jpg]

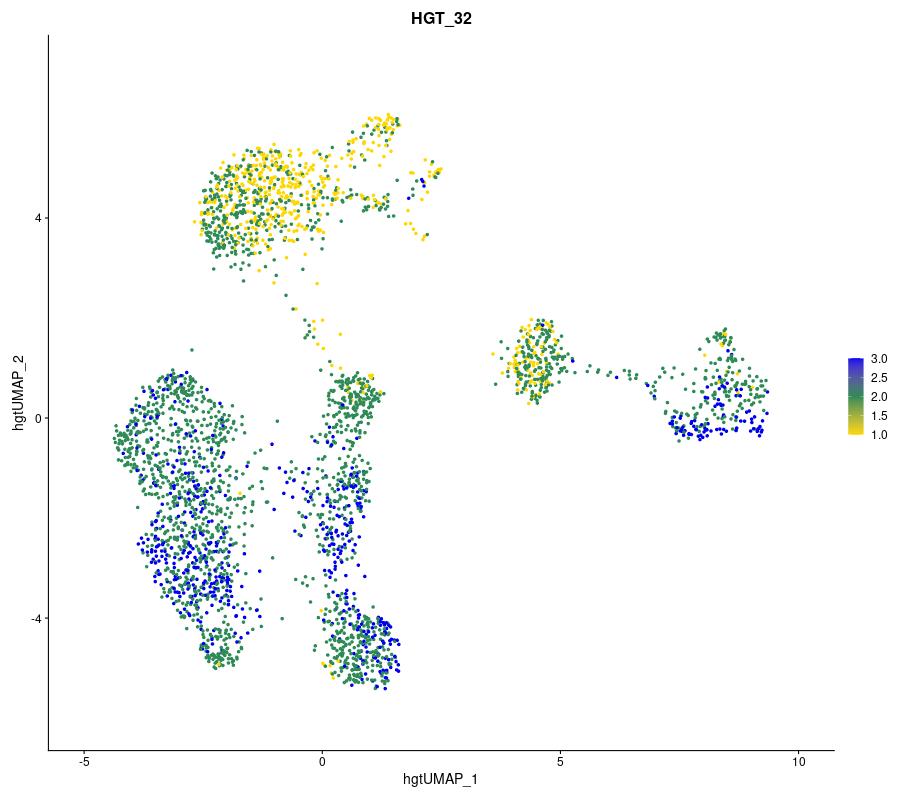

Supplement: Supplementary file 5 — Supplementary Data 3 [file 41467_2023_36559_MOESM5_ESM.zip › all128embedding/32 .jpg]

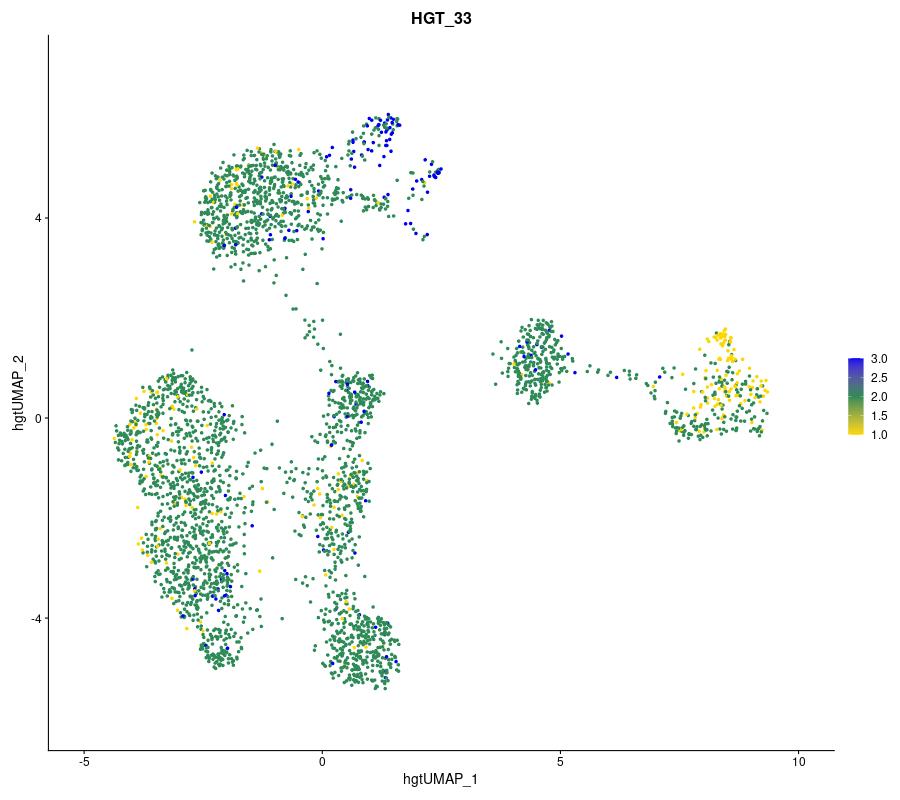

Supplement: Supplementary file 5 — Supplementary Data 3 [file 41467_2023_36559_MOESM5_ESM.zip › all128embedding/33 .jpg]

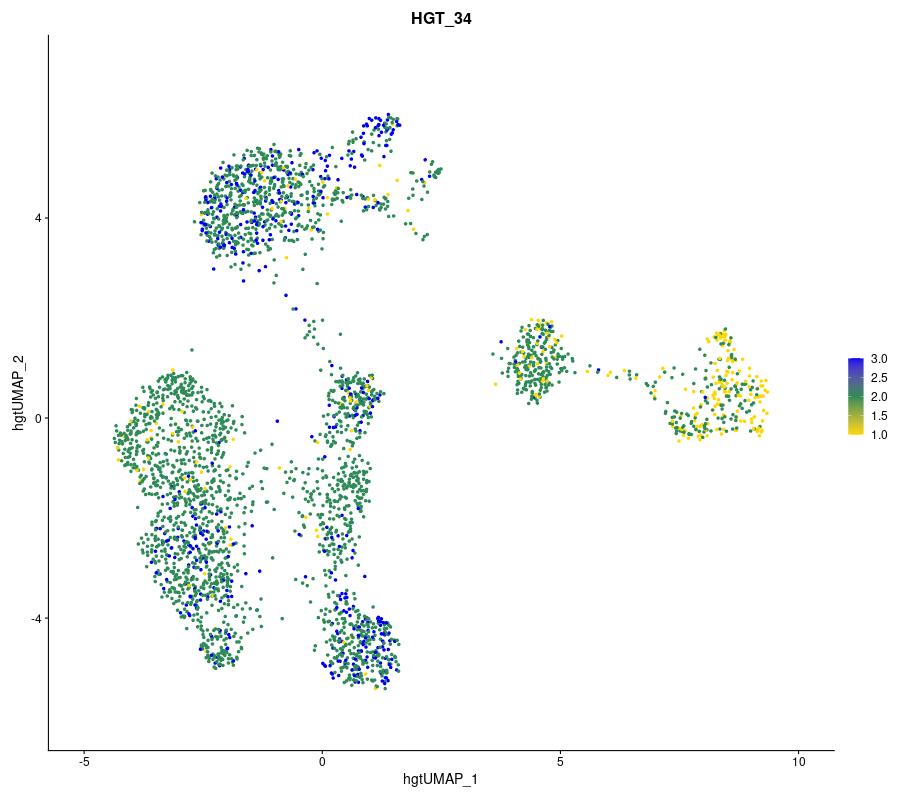

Supplement: Supplementary file 5 — Supplementary Data 3 [file 41467_2023_36559_MOESM5_ESM.zip › all128embedding/34 .jpg]

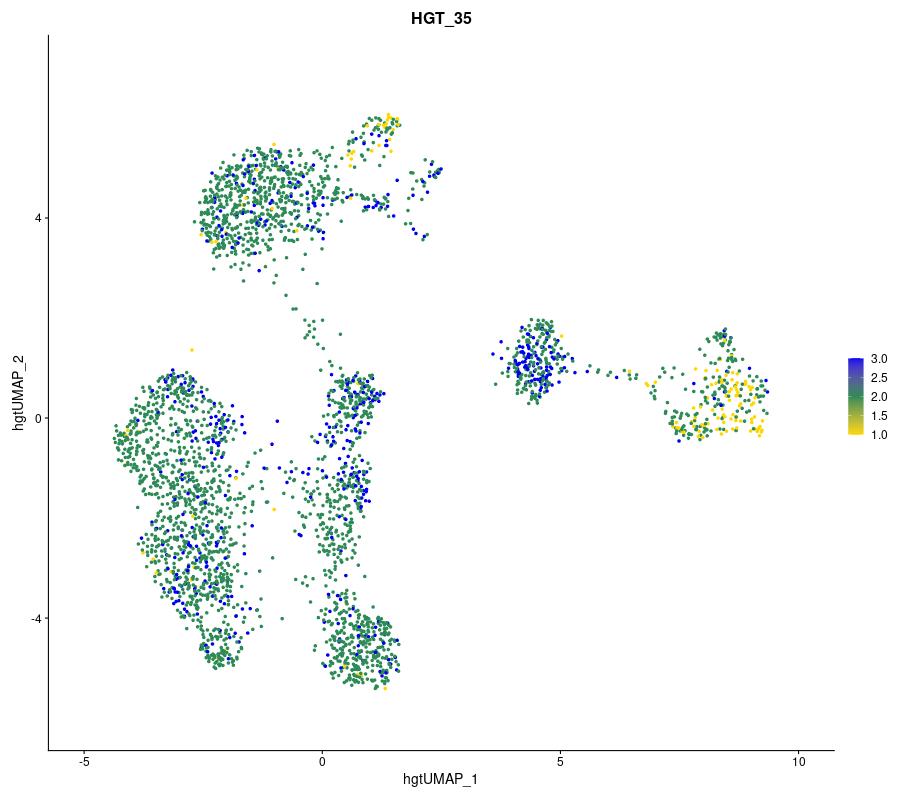

Supplement: Supplementary file 5 — Supplementary Data 3 [file 41467_2023_36559_MOESM5_ESM.zip › all128embedding/35 .jpg]

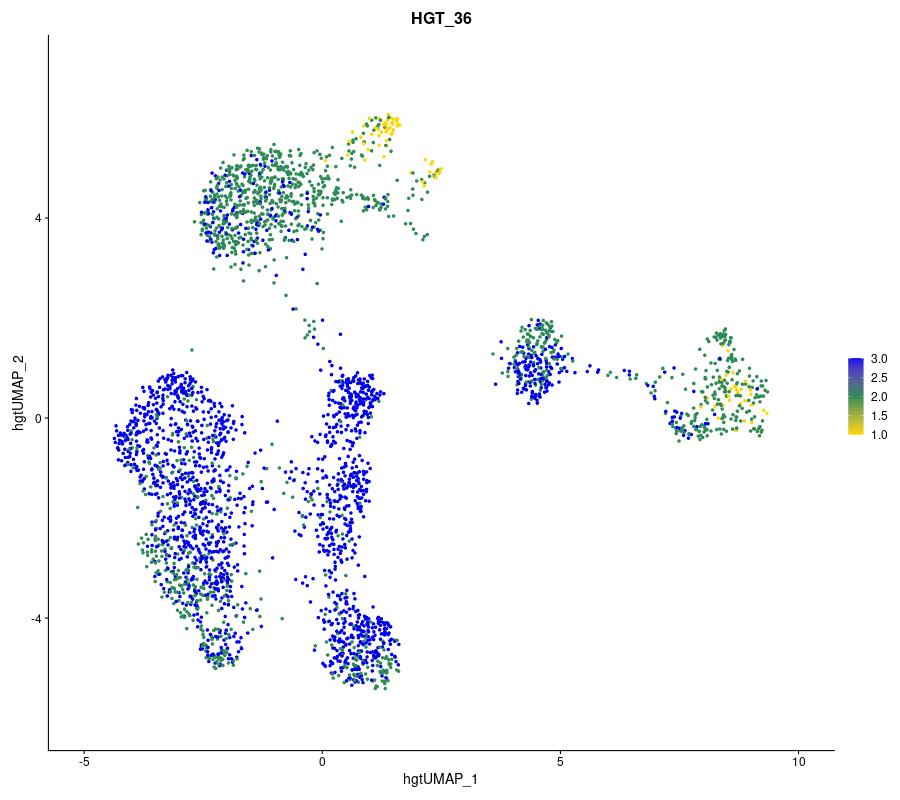

Supplement: Supplementary file 5 — Supplementary Data 3 [file 41467_2023_36559_MOESM5_ESM.zip › all128embedding/36 .jpg]

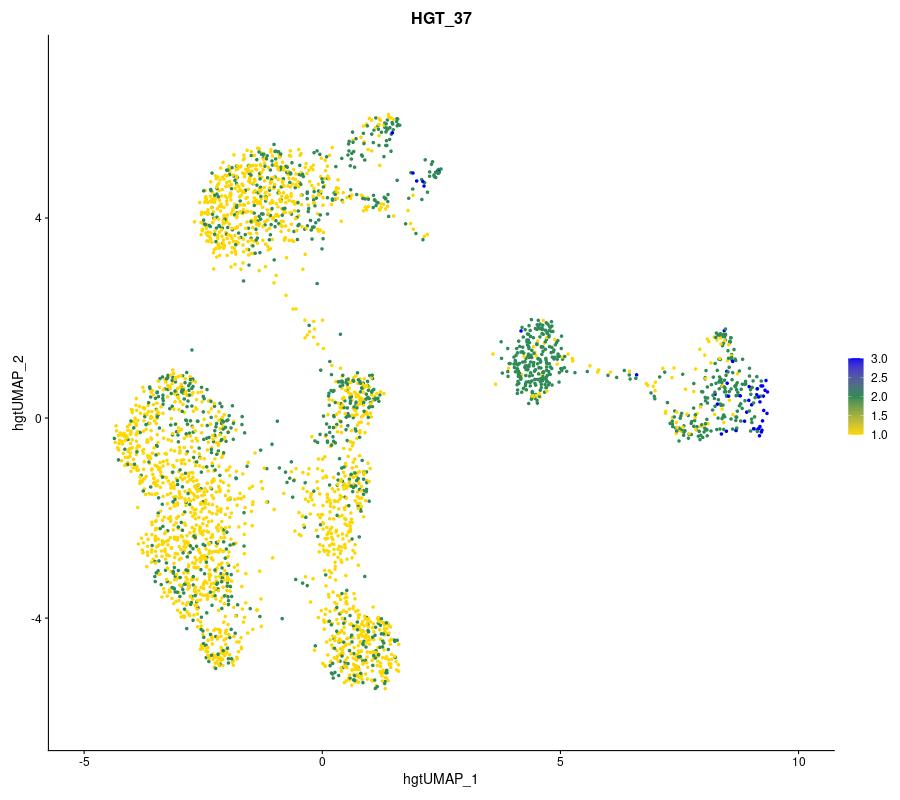

Supplement: Supplementary file 5 — Supplementary Data 3 [file 41467_2023_36559_MOESM5_ESM.zip › all128embedding/37 .jpg]

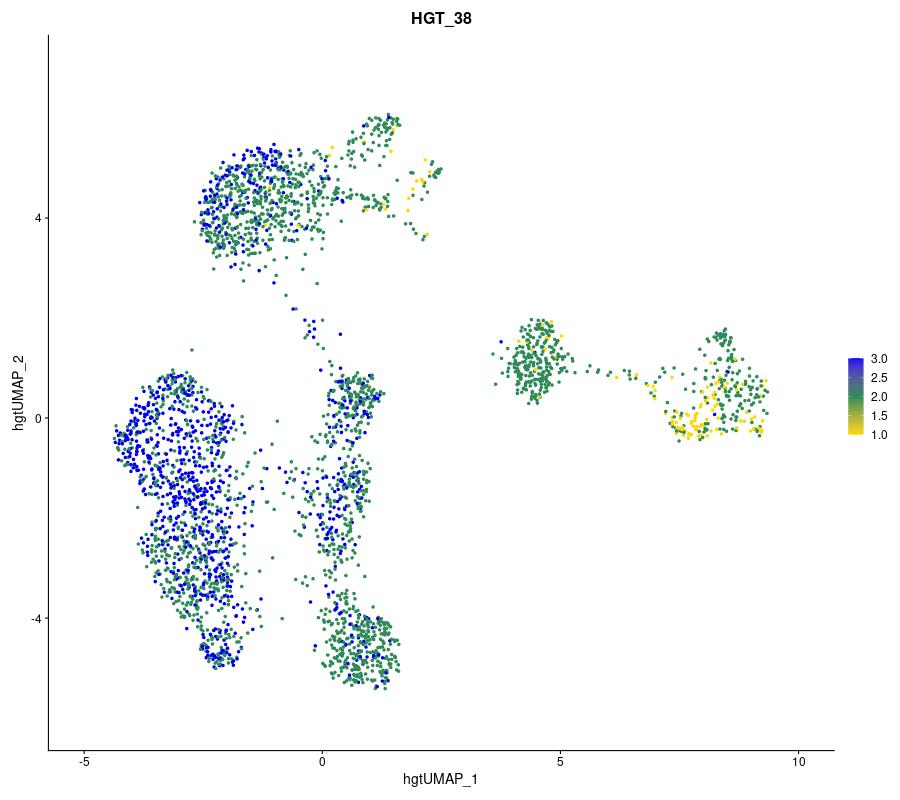

Supplement: Supplementary file 5 — Supplementary Data 3 [file 41467_2023_36559_MOESM5_ESM.zip › all128embedding/38 .jpg]

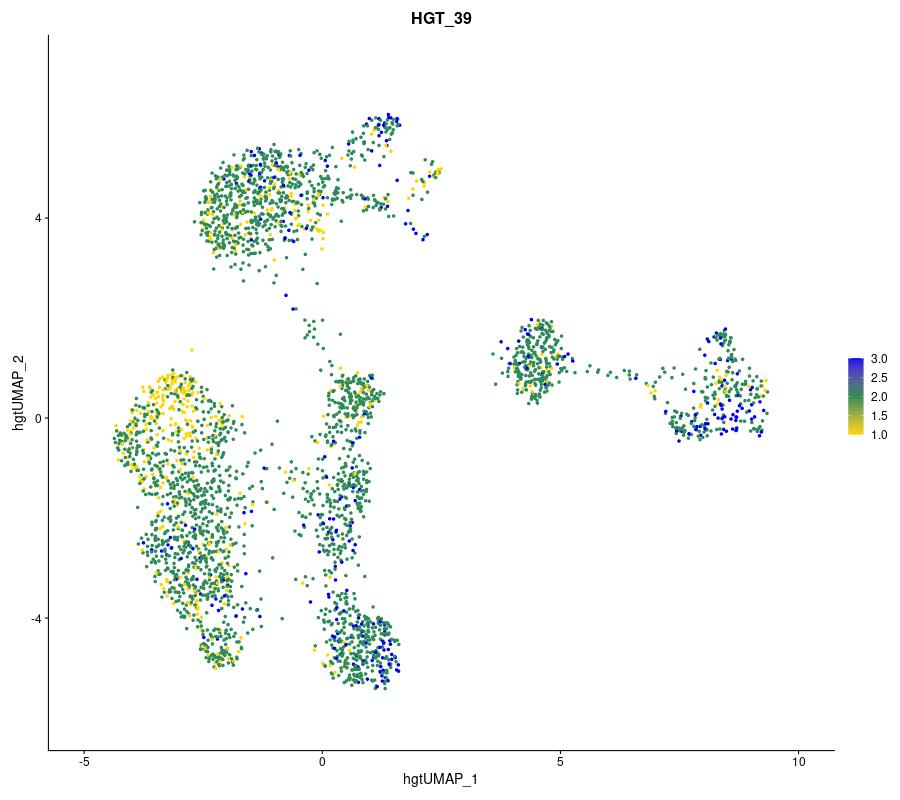

Supplement: Supplementary file 5 — Supplementary Data 3 [file 41467_2023_36559_MOESM5_ESM.zip › all128embedding/39 .jpg]

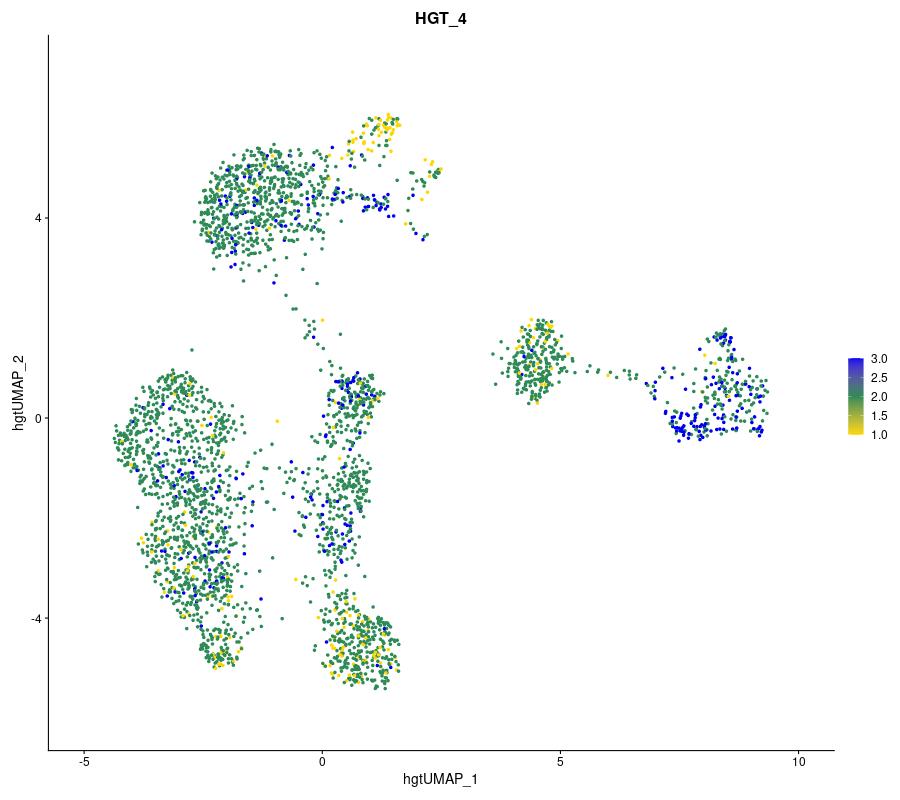

Supplement: Supplementary file 5 — Supplementary Data 3 [file 41467_2023_36559_MOESM5_ESM.zip › all128embedding/4 .jpg]

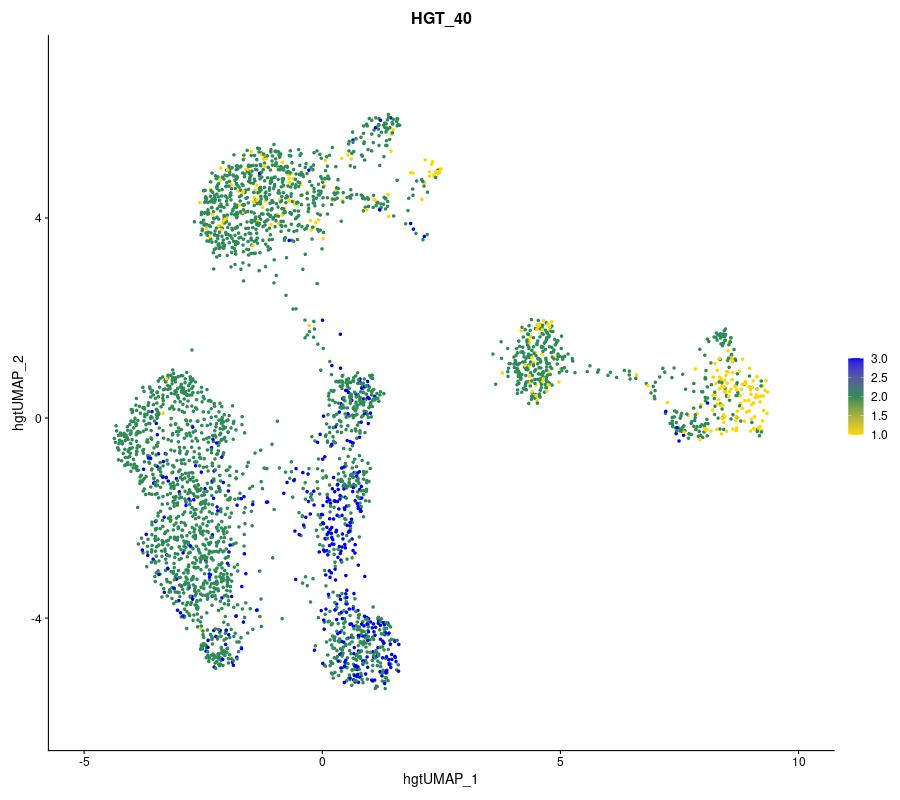

Supplement: Supplementary file 5 — Supplementary Data 3 [file 41467_2023_36559_MOESM5_ESM.zip › all128embedding/40 .jpg]

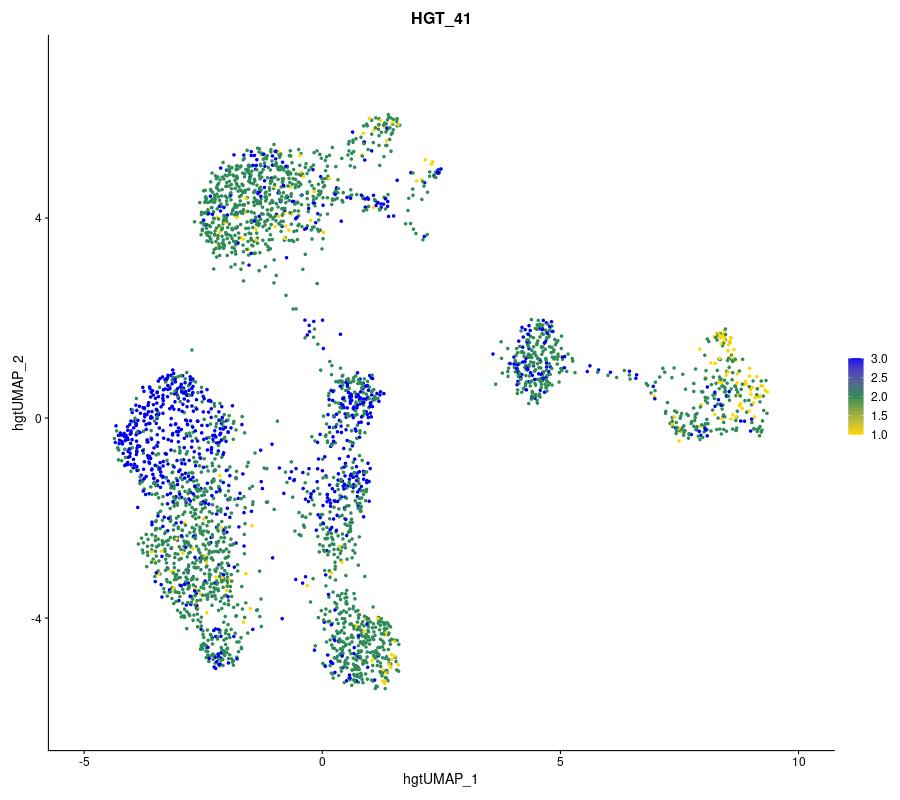

Supplement: Supplementary file 5 — Supplementary Data 3 [file 41467_2023_36559_MOESM5_ESM.zip › all128embedding/41 .jpg]

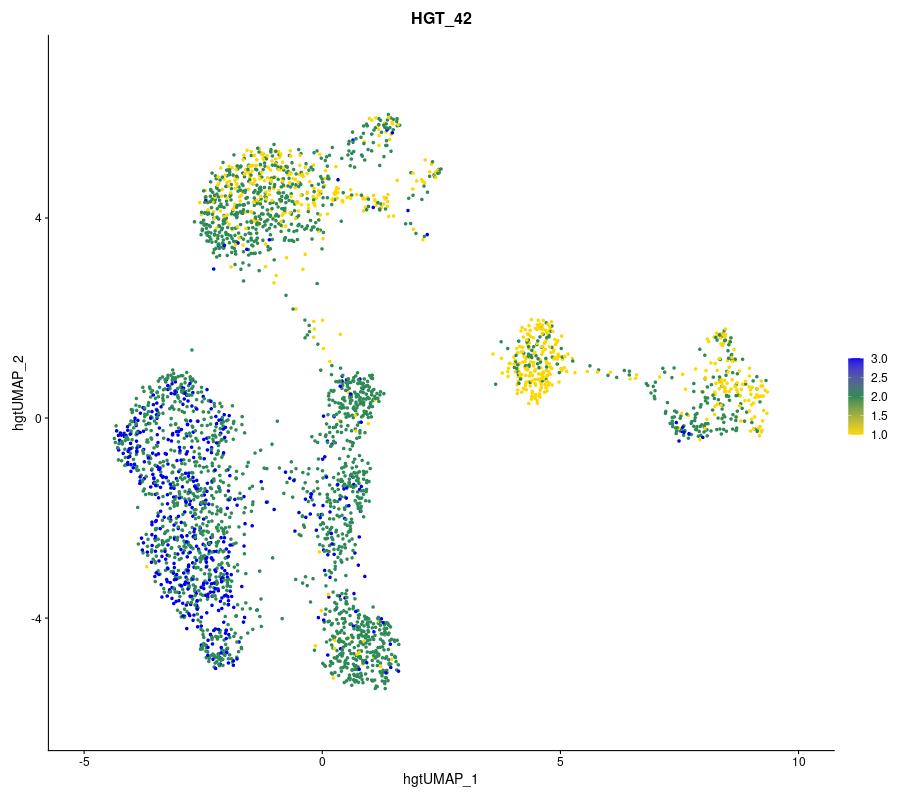

Supplement: Supplementary file 5 — Supplementary Data 3 [file 41467_2023_36559_MOESM5_ESM.zip › all128embedding/42 .jpg]

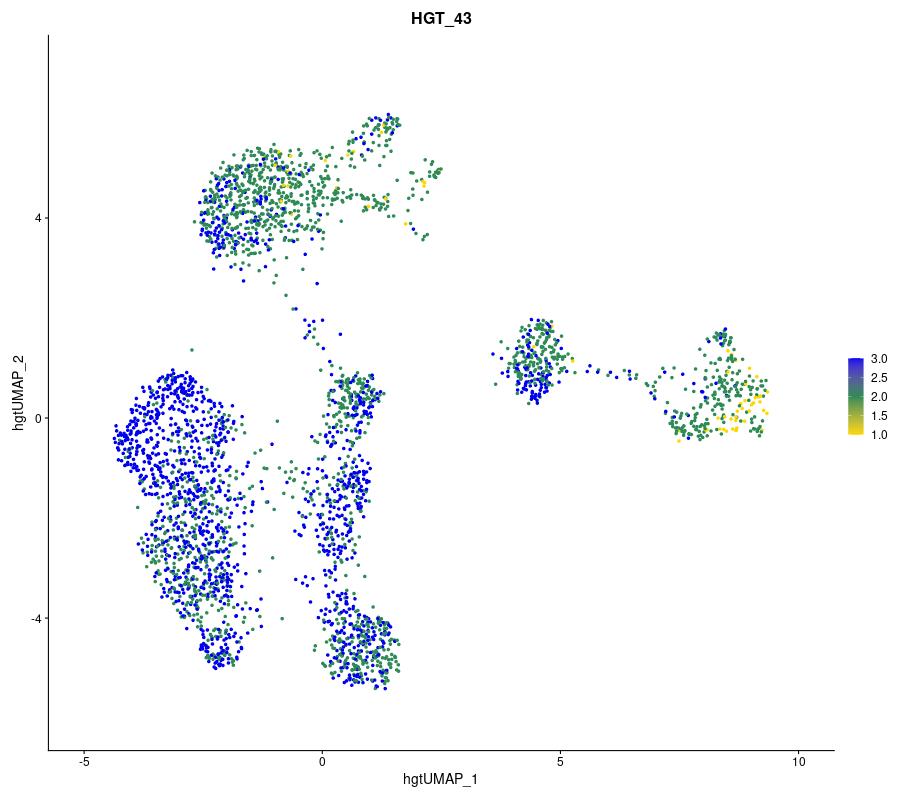

Supplement: Supplementary file 5 — Supplementary Data 3 [file 41467_2023_36559_MOESM5_ESM.zip › all128embedding/43 .jpg]

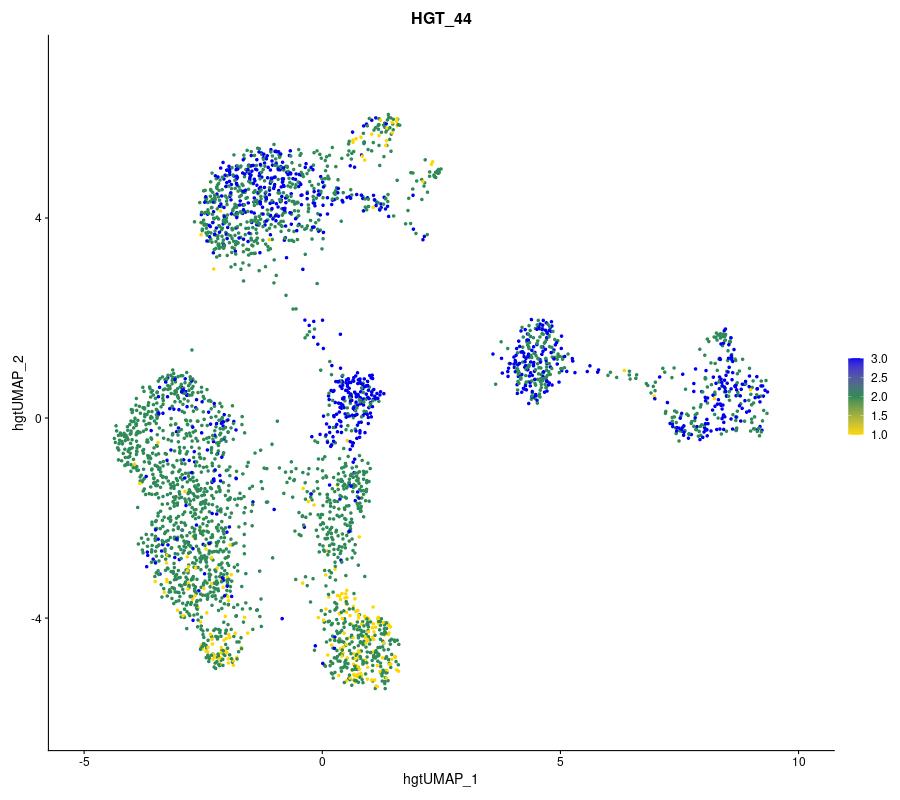

Supplement: Supplementary file 5 — Supplementary Data 3 [file 41467_2023_36559_MOESM5_ESM.zip › all128embedding/44 .jpg]

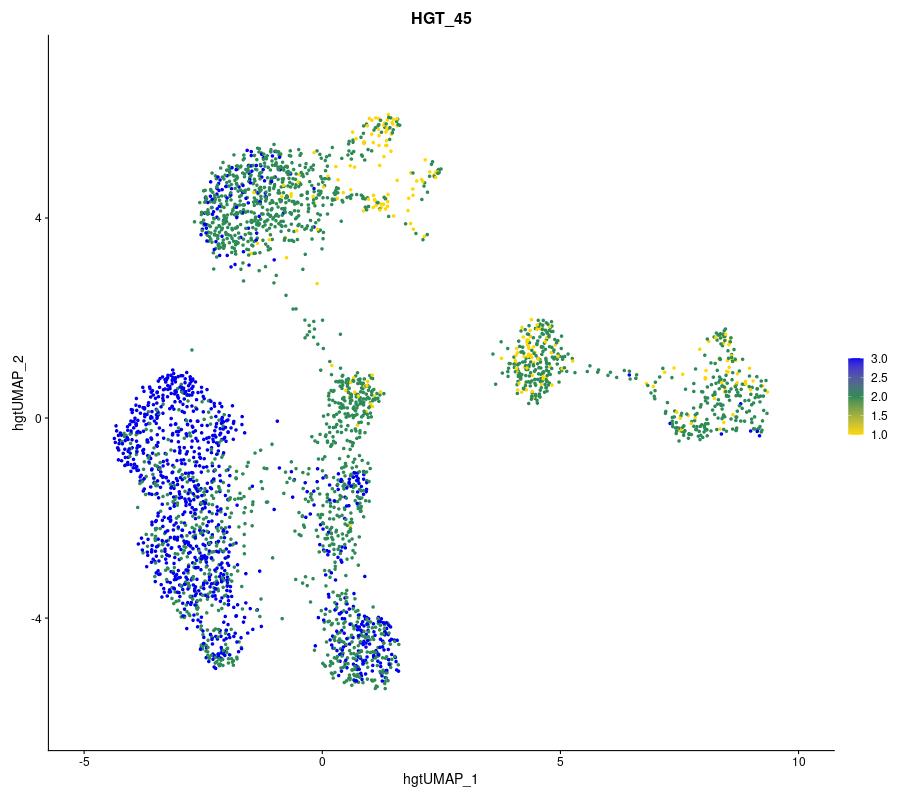

Supplement: Supplementary file 5 — Supplementary Data 3 [file 41467_2023_36559_MOESM5_ESM.zip › all128embedding/45 .jpg]

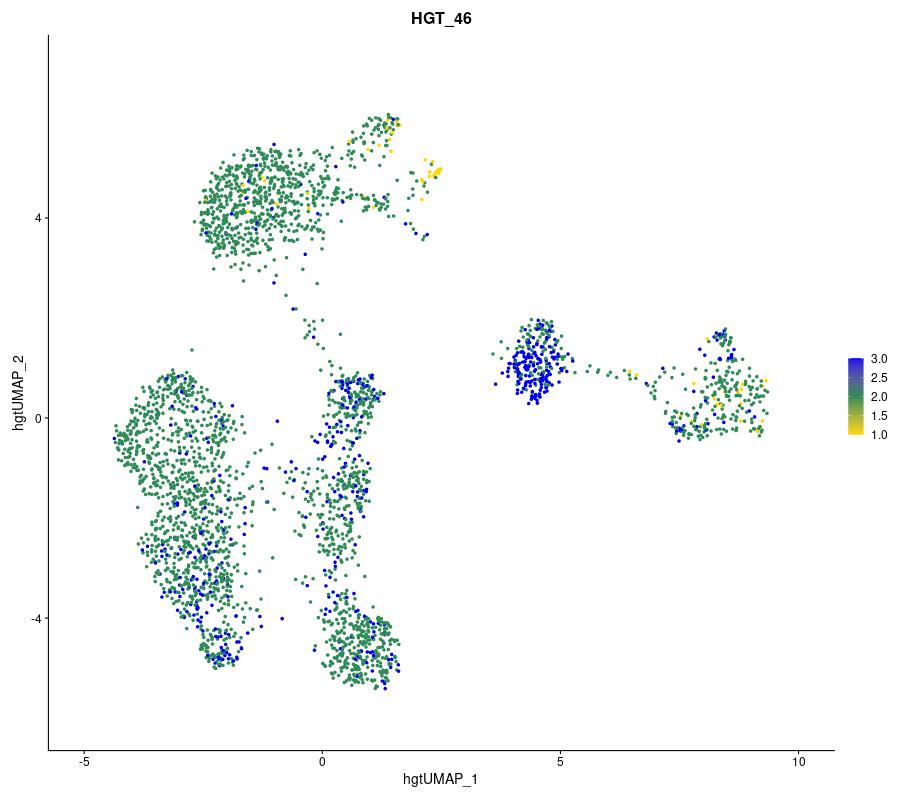

Supplement: Supplementary file 5 — Supplementary Data 3 [file 41467_2023_36559_MOESM5_ESM.zip › all128embedding/46 .jpg]

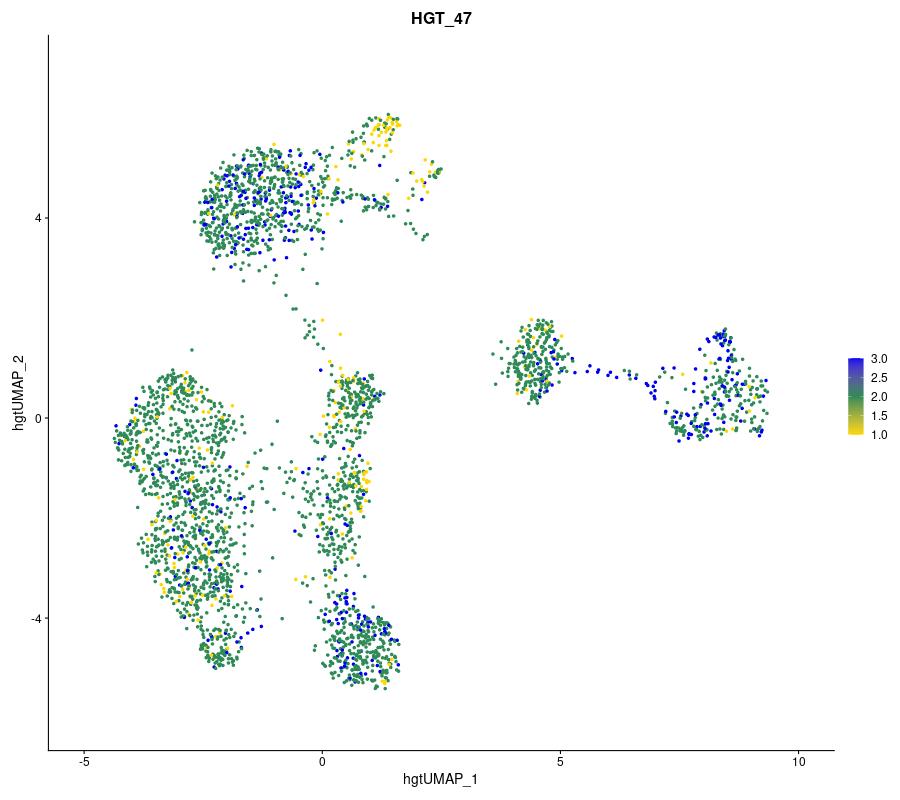

Supplement: Supplementary file 5 — Supplementary Data 3 [file 41467_2023_36559_MOESM5_ESM.zip › all128embedding/47 .jpg]

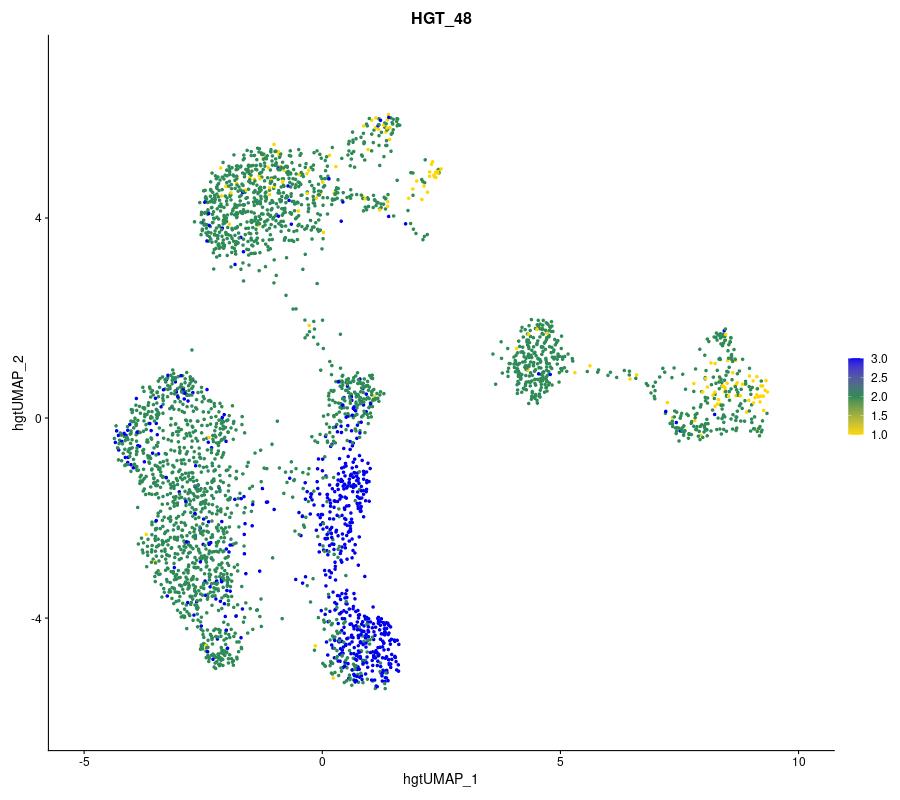

Supplement: Supplementary file 5 — Supplementary Data 3 [file 41467_2023_36559_MOESM5_ESM.zip › all128embedding/48 .jpg]

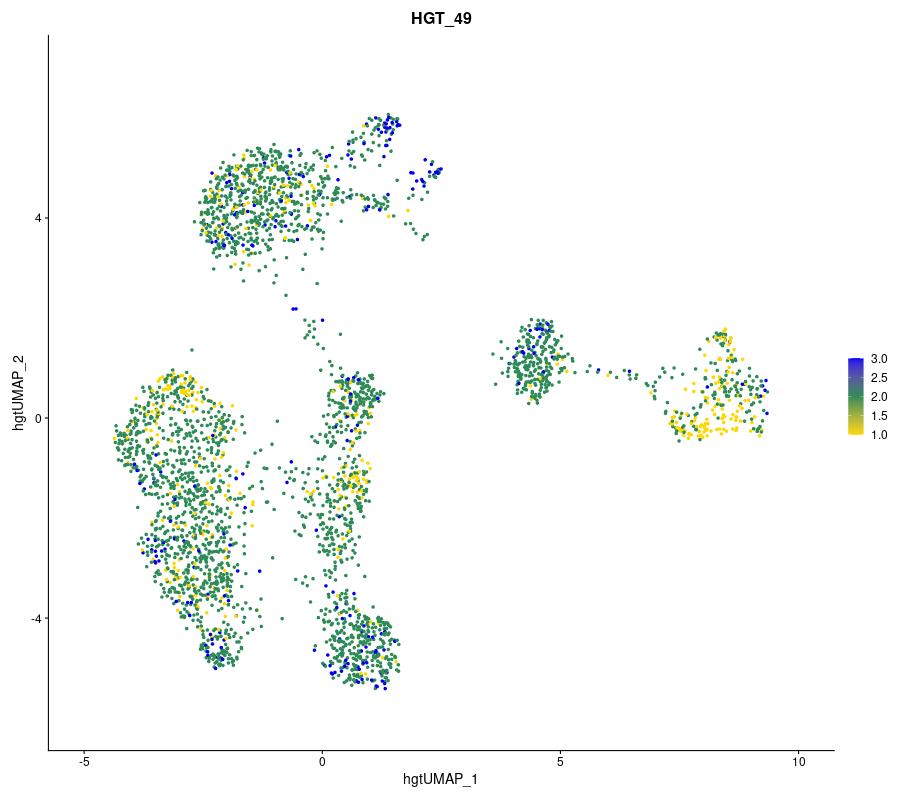

Supplement: Supplementary file 5 — Supplementary Data 3 [file 41467_2023_36559_MOESM5_ESM.zip › all128embedding/49 .jpg]

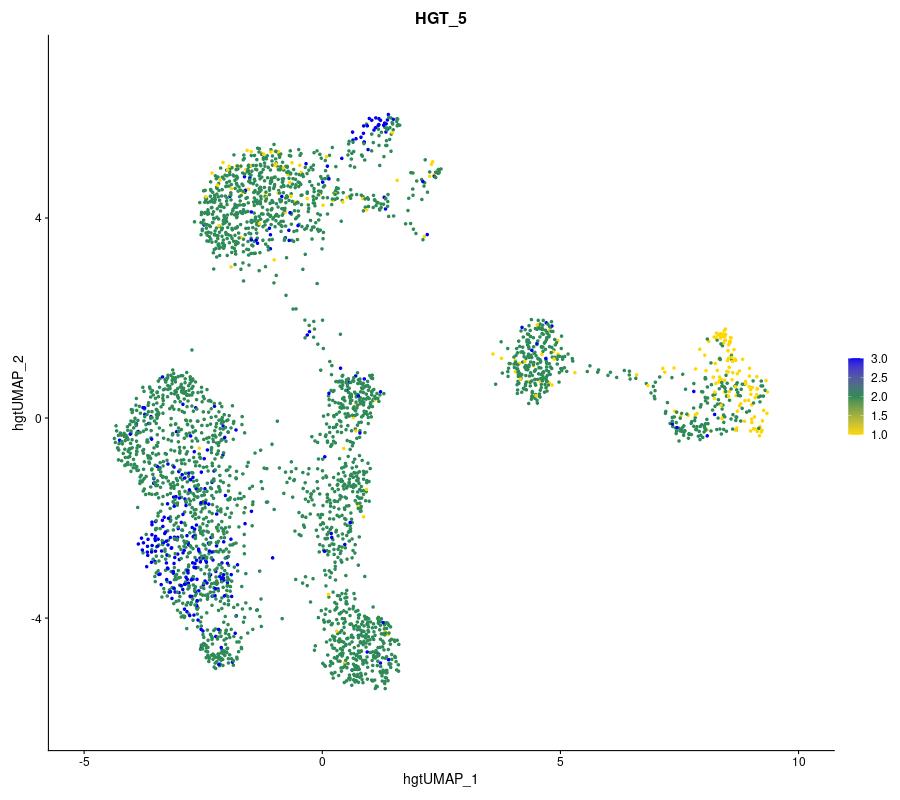

Supplement: Supplementary file 5 — Supplementary Data 3 [file 41467_2023_36559_MOESM5_ESM.zip › all128embedding/5 .jpg]

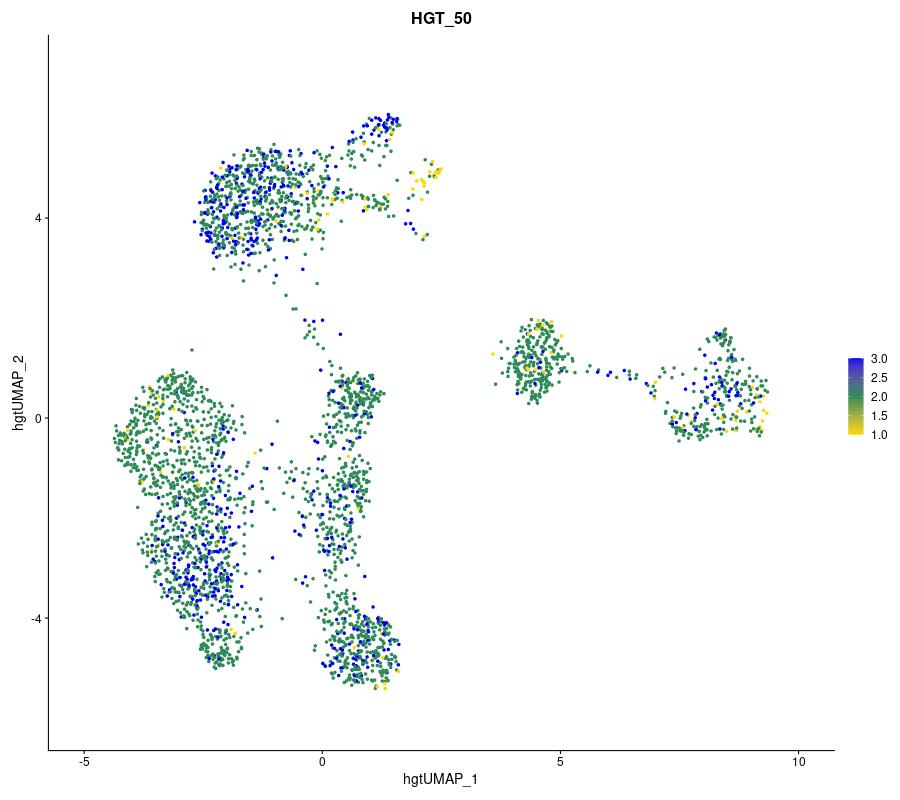

Supplement: Supplementary file 5 — Supplementary Data 3 [file 41467_2023_36559_MOESM5_ESM.zip › all128embedding/50 .jpg]

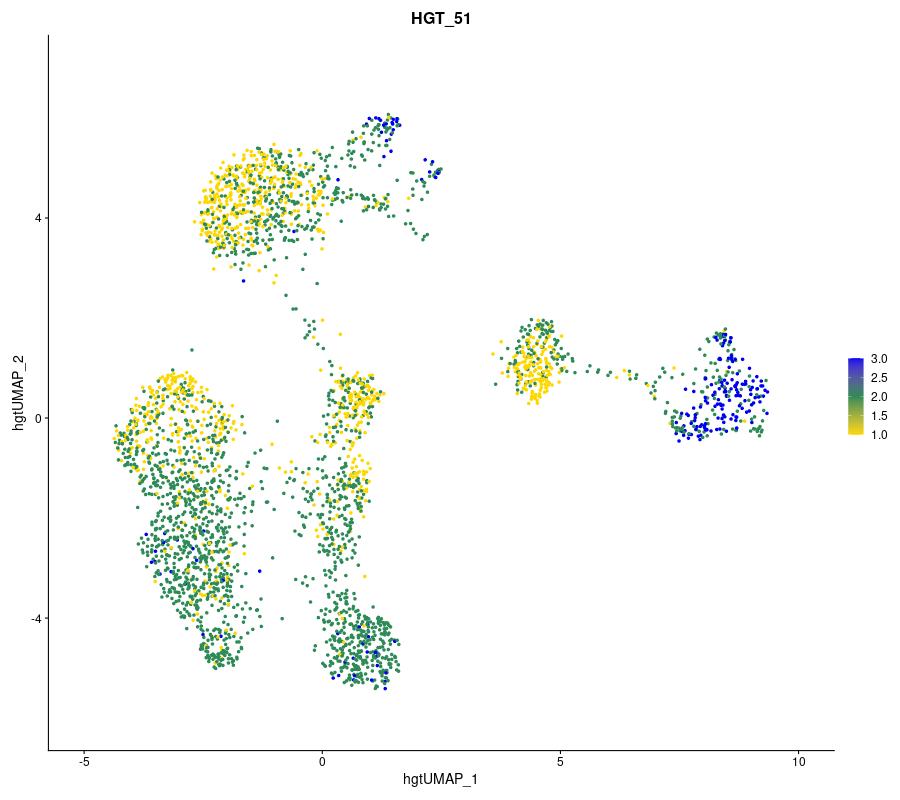

Supplement: Supplementary file 5 — Supplementary Data 3 [file 41467_2023_36559_MOESM5_ESM.zip › all128embedding/51 .jpg]

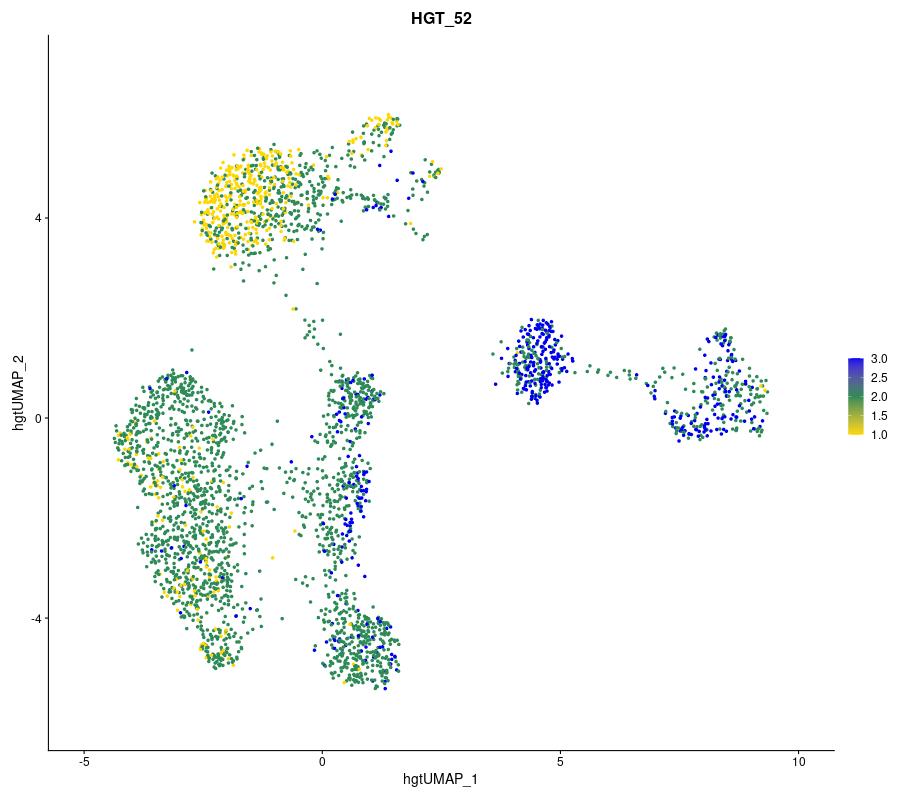

Supplement: Supplementary file 5 — Supplementary Data 3 [file 41467_2023_36559_MOESM5_ESM.zip › all128embedding/52 .jpg]

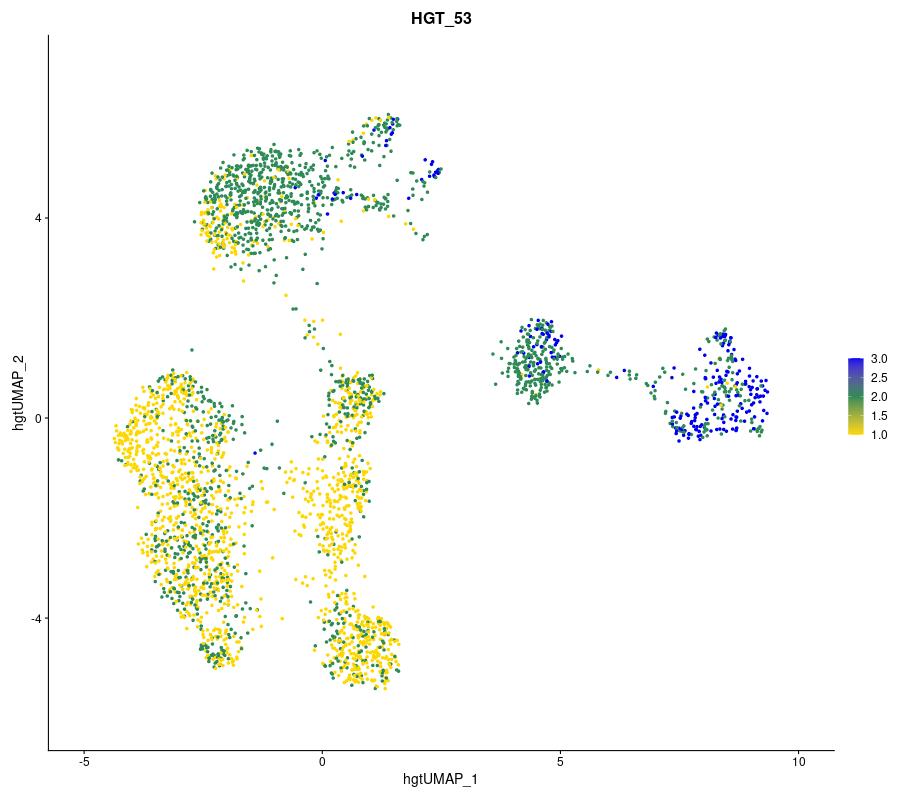

Supplement: Supplementary file 5 — Supplementary Data 3 [file 41467_2023_36559_MOESM5_ESM.zip › all128embedding/53 .jpg]

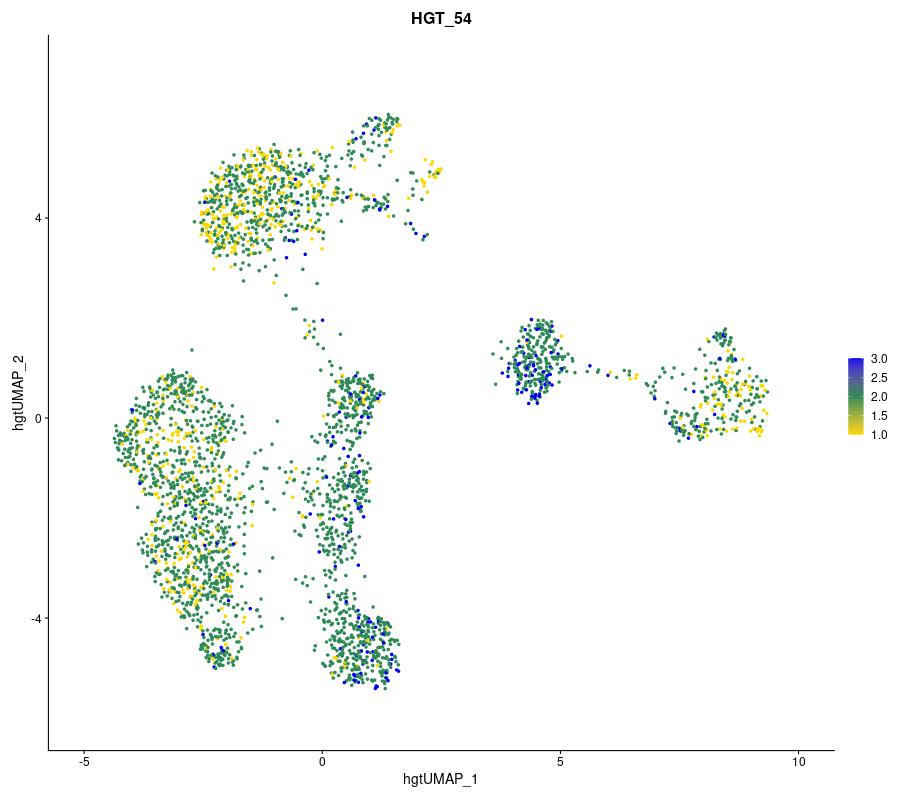

Supplement: Supplementary file 5 — Supplementary Data 3 [file 41467_2023_36559_MOESM5_ESM.zip › all128embedding/54 .jpg]

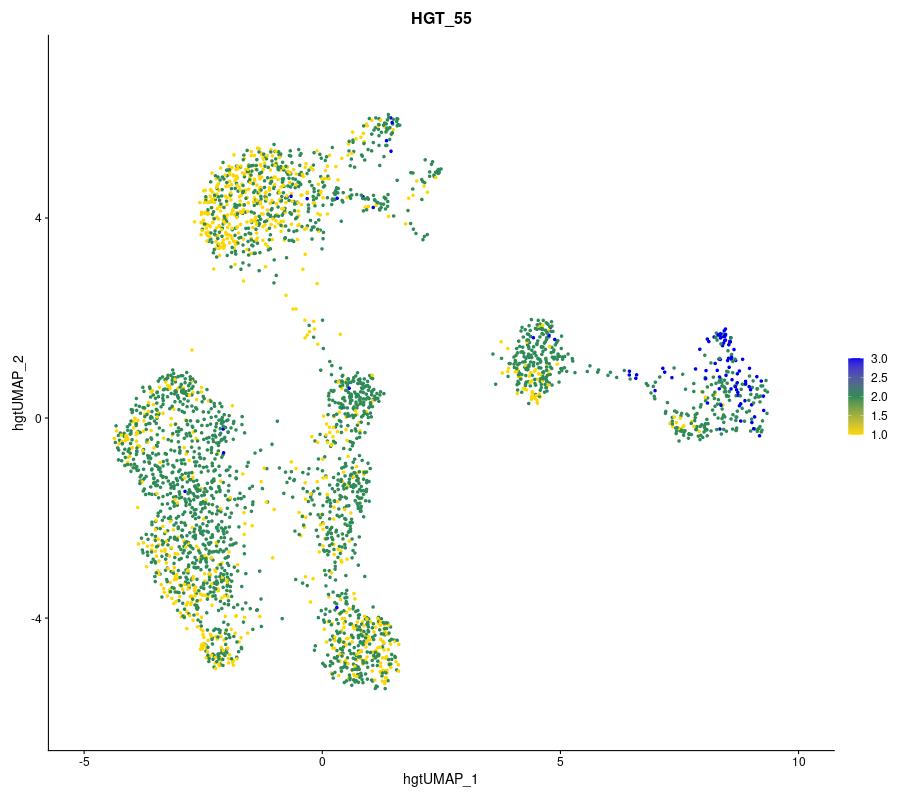

Supplement: Supplementary file 5 — Supplementary Data 3 [file 41467_2023_36559_MOESM5_ESM.zip › all128embedding/55 .jpg]

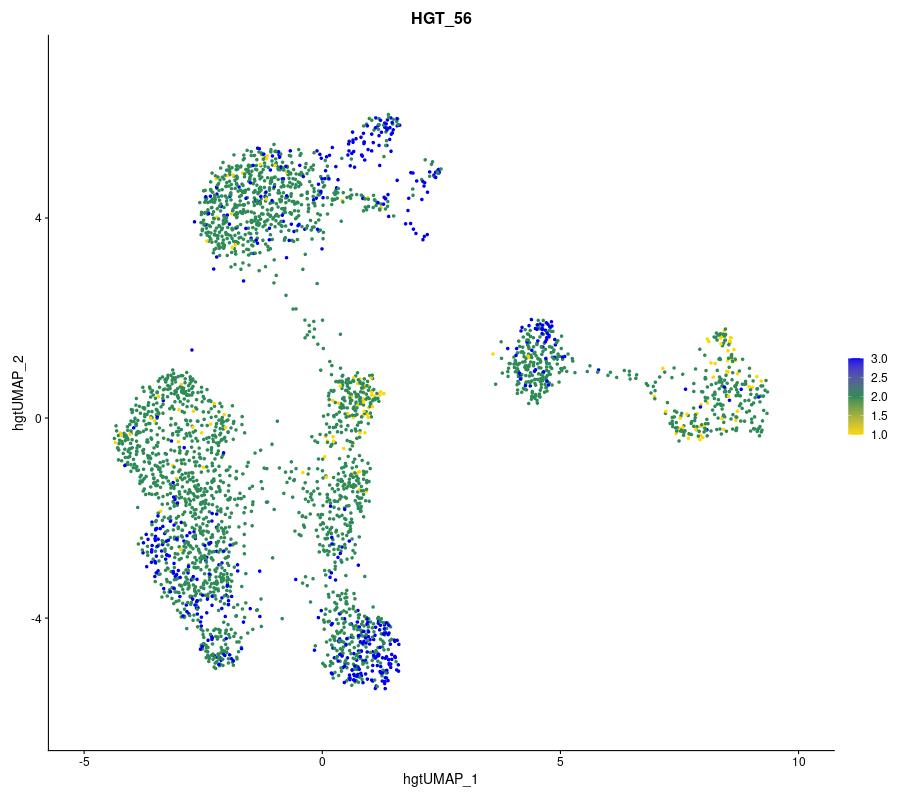

Supplement: Supplementary file 5 — Supplementary Data 3 [file 41467_2023_36559_MOESM5_ESM.zip › all128embedding/56 .jpg]

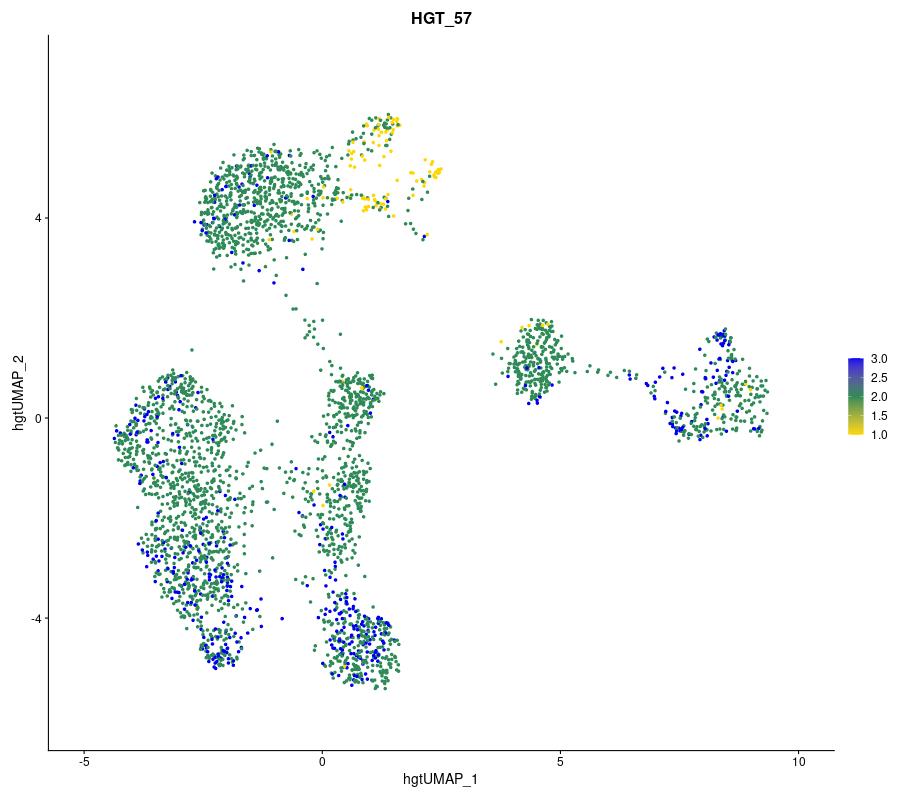

Supplement: Supplementary file 5 — Supplementary Data 3 [file 41467_2023_36559_MOESM5_ESM.zip › all128embedding/57 .jpg]

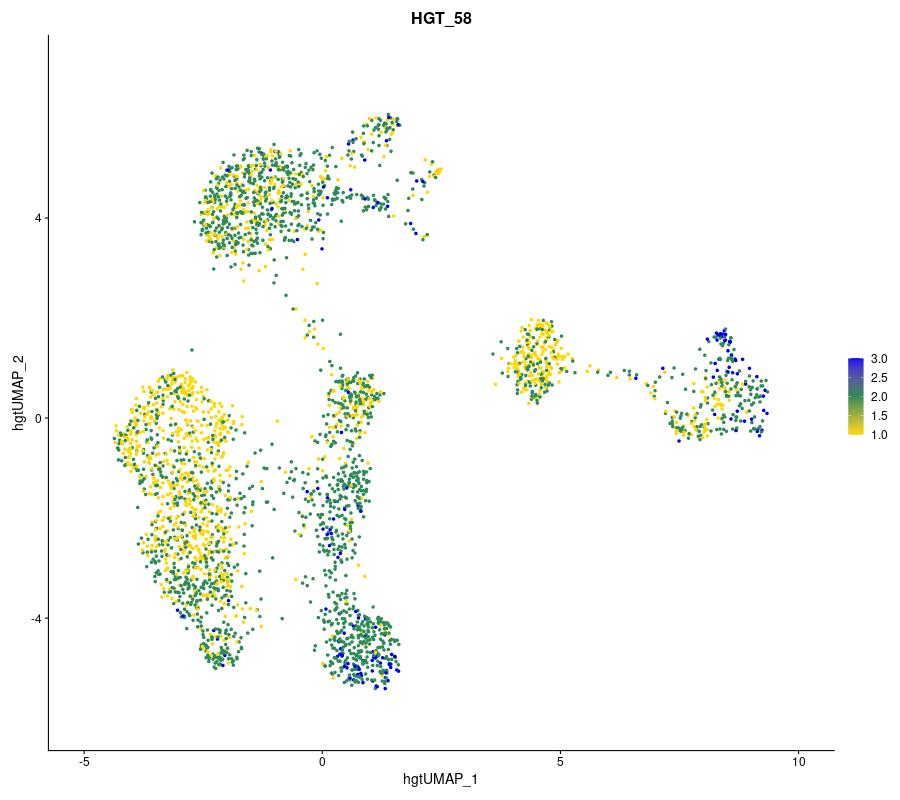

Supplement: Supplementary file 5 — Supplementary Data 3 [file 41467_2023_36559_MOESM5_ESM.zip › all128embedding/58 .jpg]

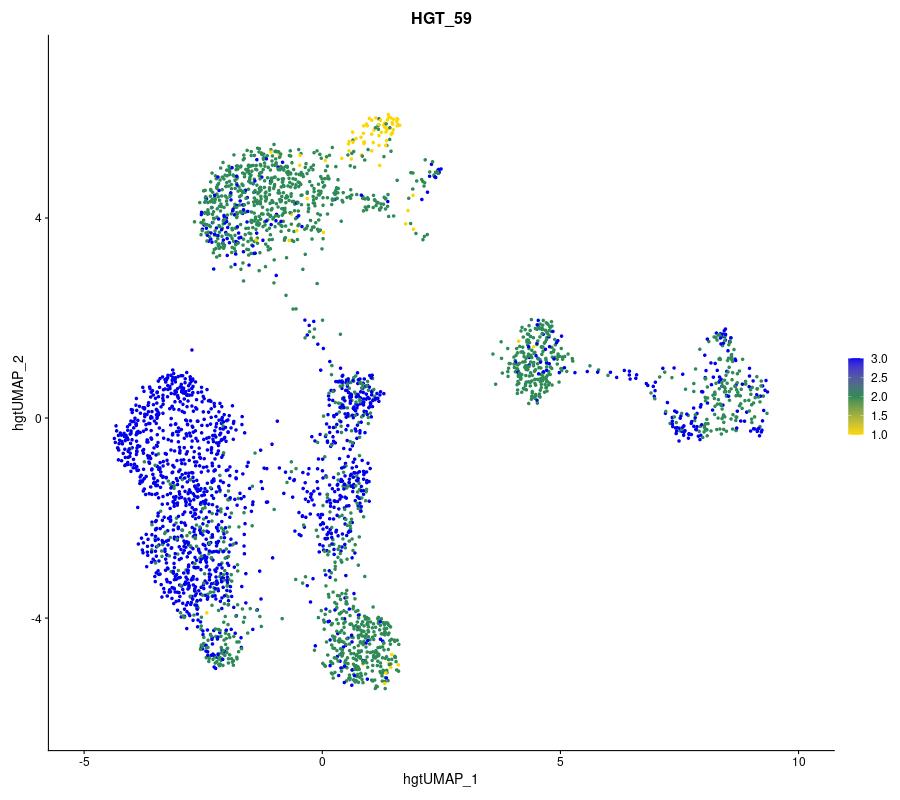

Supplement: Supplementary file 5 — Supplementary Data 3 [file 41467_2023_36559_MOESM5_ESM.zip › all128embedding/59 .jpg]

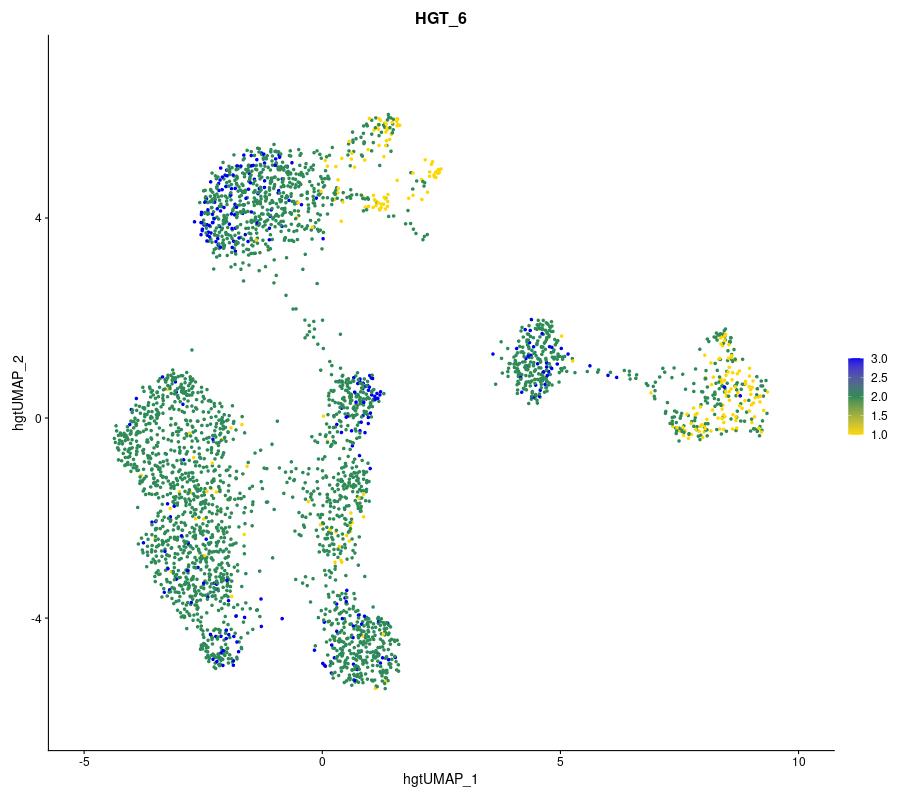

Supplement: Supplementary file 5 — Supplementary Data 3 [file 41467_2023_36559_MOESM5_ESM.zip › all128embedding/6 .jpg]

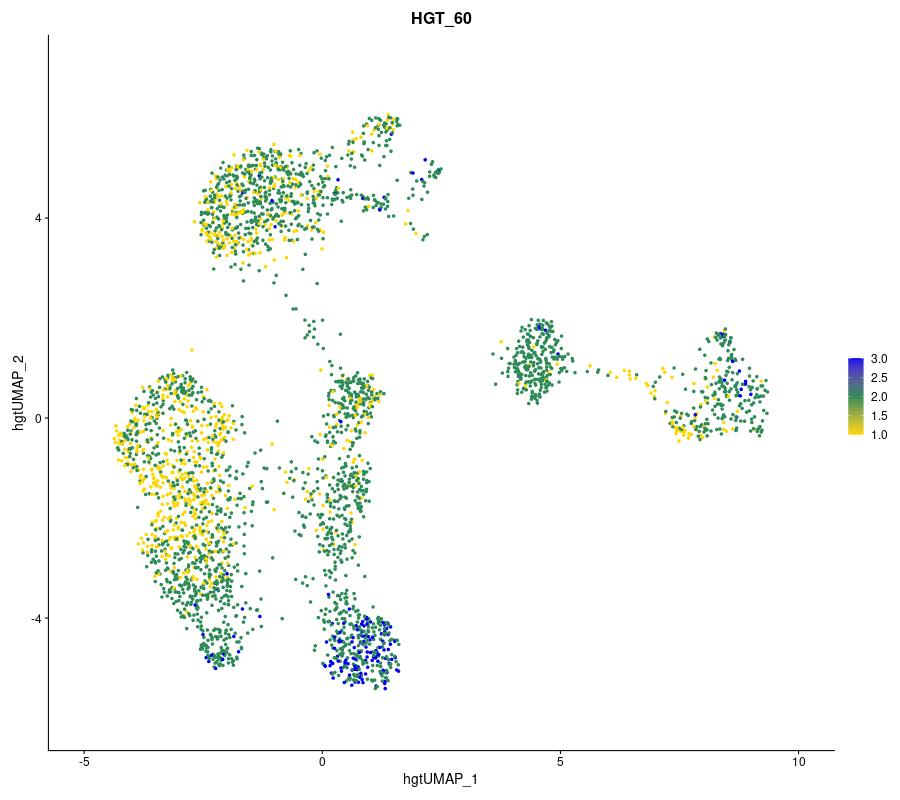

Supplement: Supplementary file 5 — Supplementary Data 3 [file 41467_2023_36559_MOESM5_ESM.zip › all128embedding/60 .jpg]

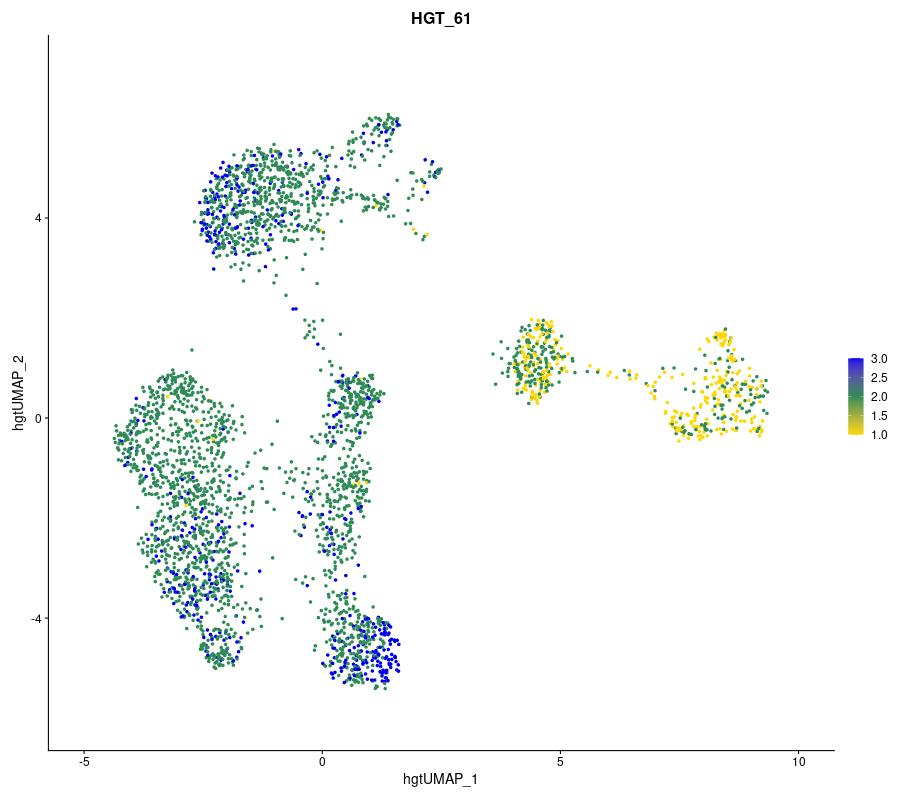

Supplement: Supplementary file 5 — Supplementary Data 3 [file 41467_2023_36559_MOESM5_ESM.zip › all128embedding/61 .jpg]

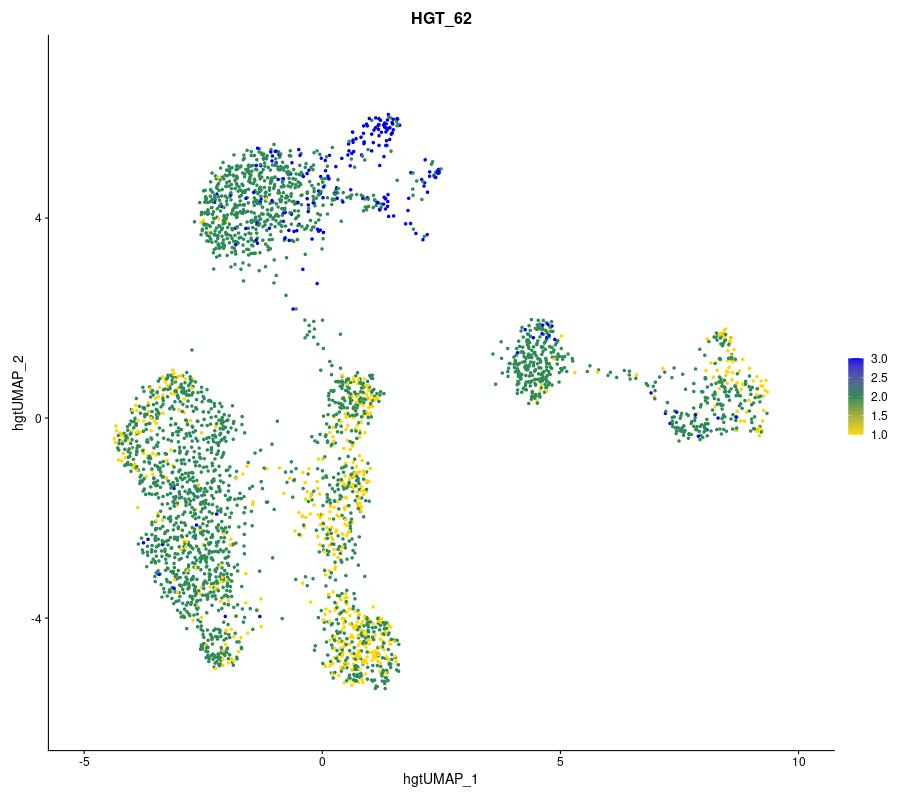

Supplement: Supplementary file 5 — Supplementary Data 3 [file 41467_2023_36559_MOESM5_ESM.zip › all128embedding/62 .jpg]

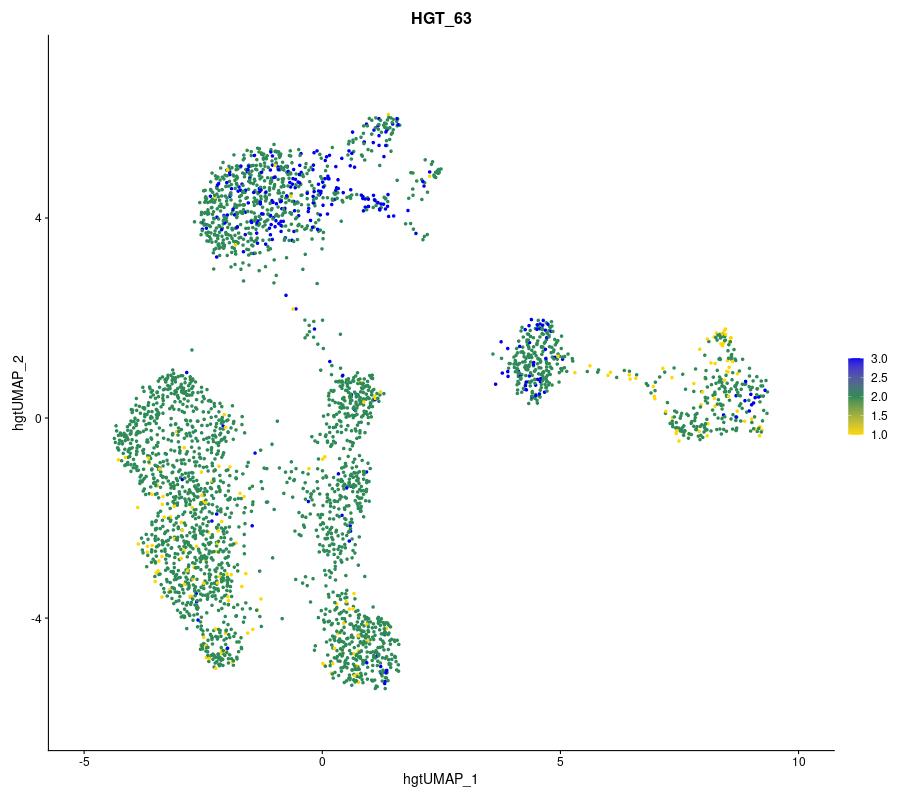

Supplement: Supplementary file 5 — Supplementary Data 3 [file 41467_2023_36559_MOESM5_ESM.zip › all128embedding/63 .jpg]

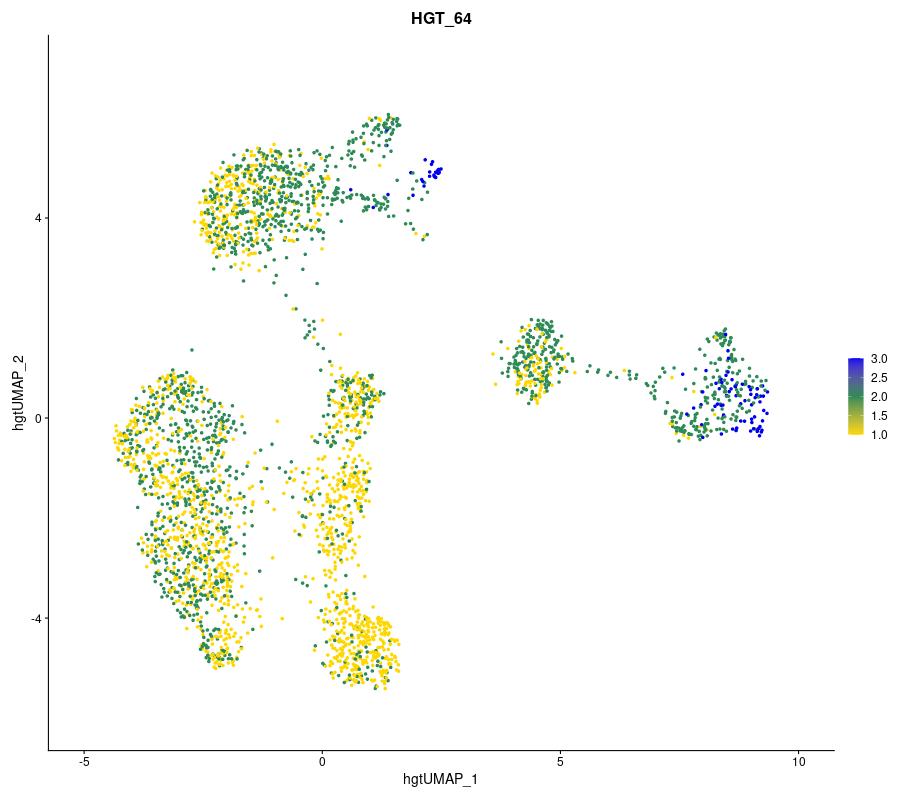

Supplement: Supplementary file 5 — Supplementary Data 3 [file 41467_2023_36559_MOESM5_ESM.zip › all128embedding/64 .jpg]

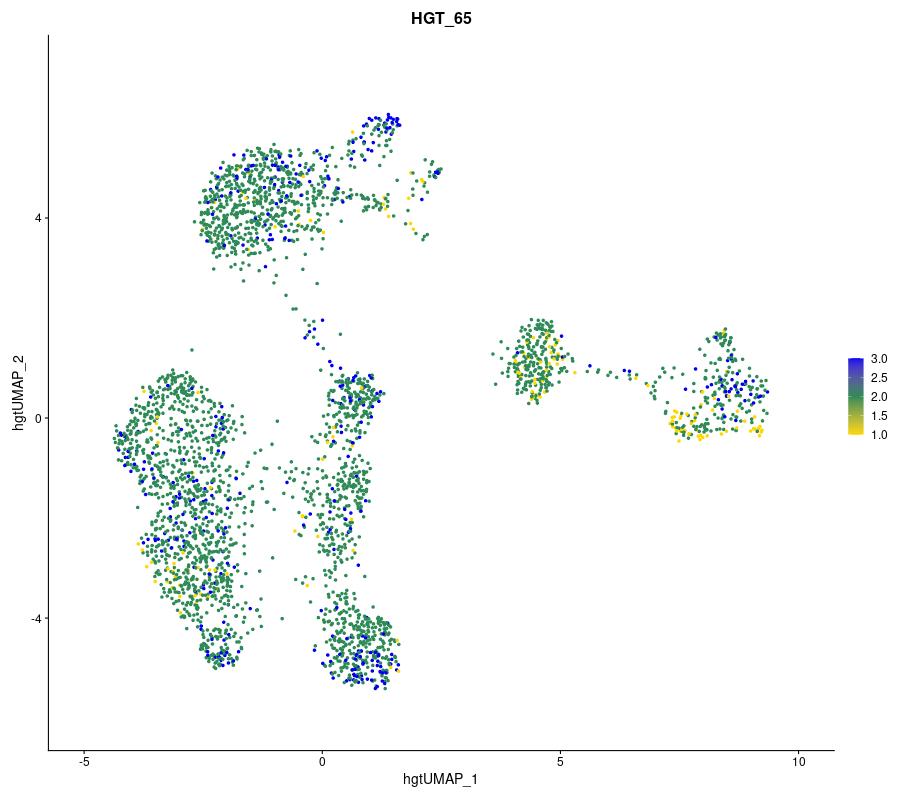

Supplement: Supplementary file 5 — Supplementary Data 3 [file 41467_2023_36559_MOESM5_ESM.zip › all128embedding/65 .jpg]

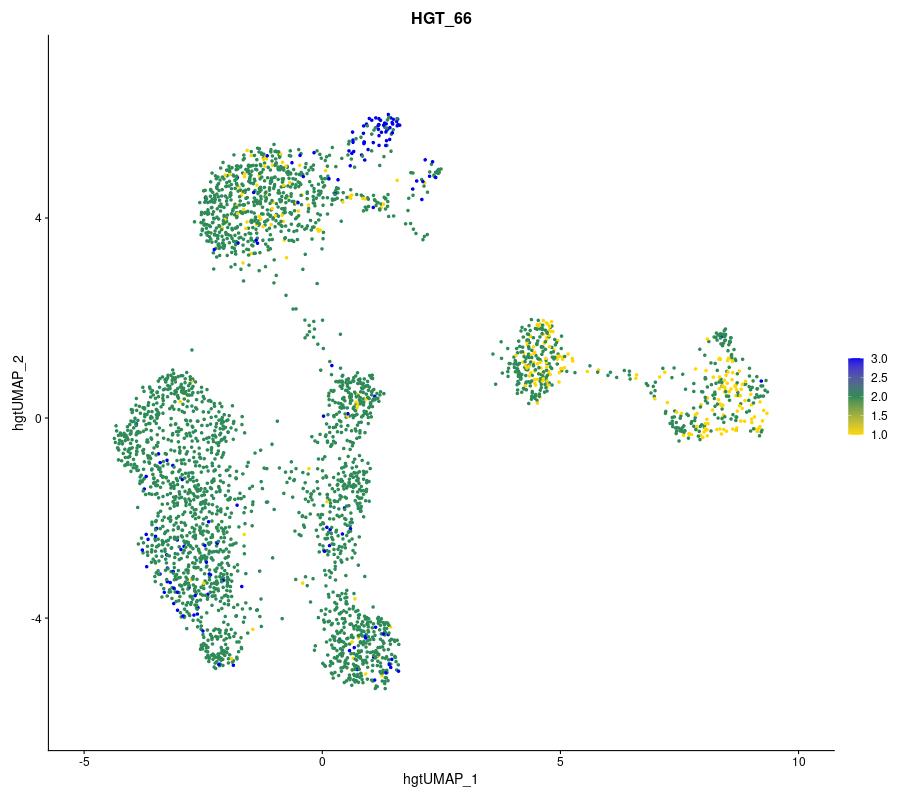

Supplement: Supplementary file 5 — Supplementary Data 3 [file 41467_2023_36559_MOESM5_ESM.zip › all128embedding/66 .jpg]

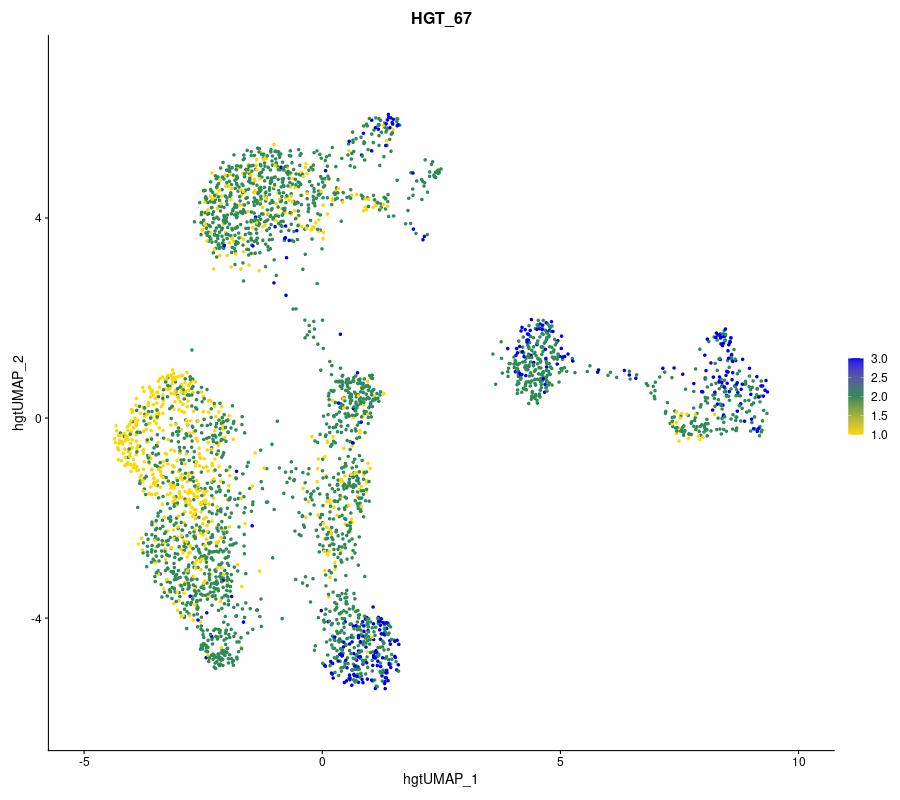

Supplement: Supplementary file 5 — Supplementary Data 3 [file 41467_2023_36559_MOESM5_ESM.zip › all128embedding/67 .jpg]

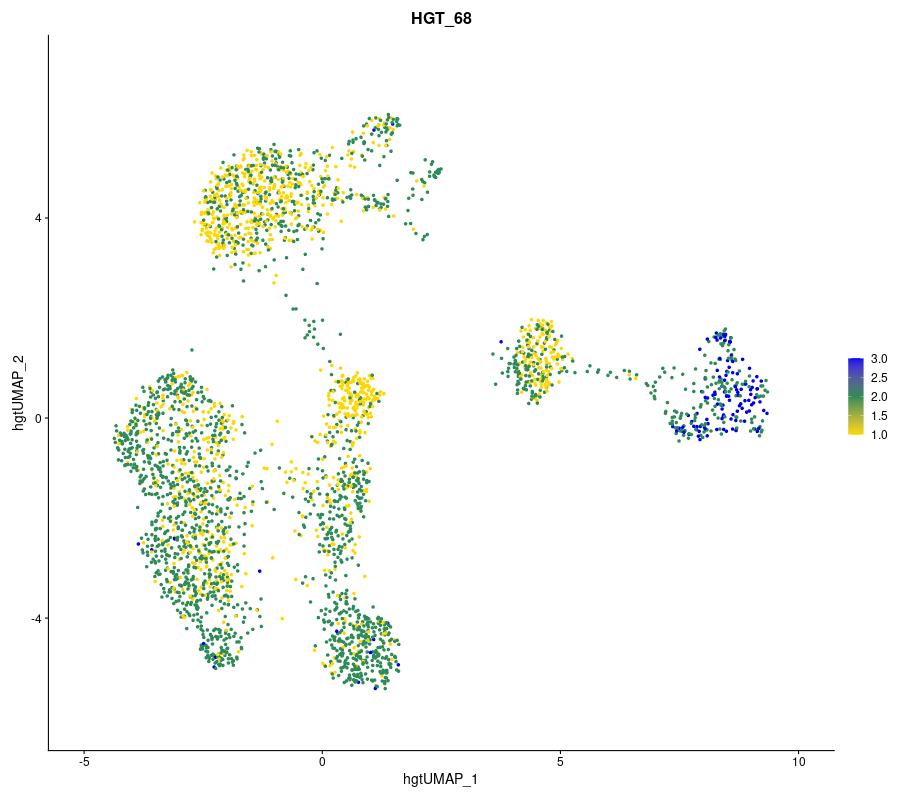

Supplement: Supplementary file 5 — Supplementary Data 3 [file 41467_2023_36559_MOESM5_ESM.zip › all128embedding/68 .jpg]

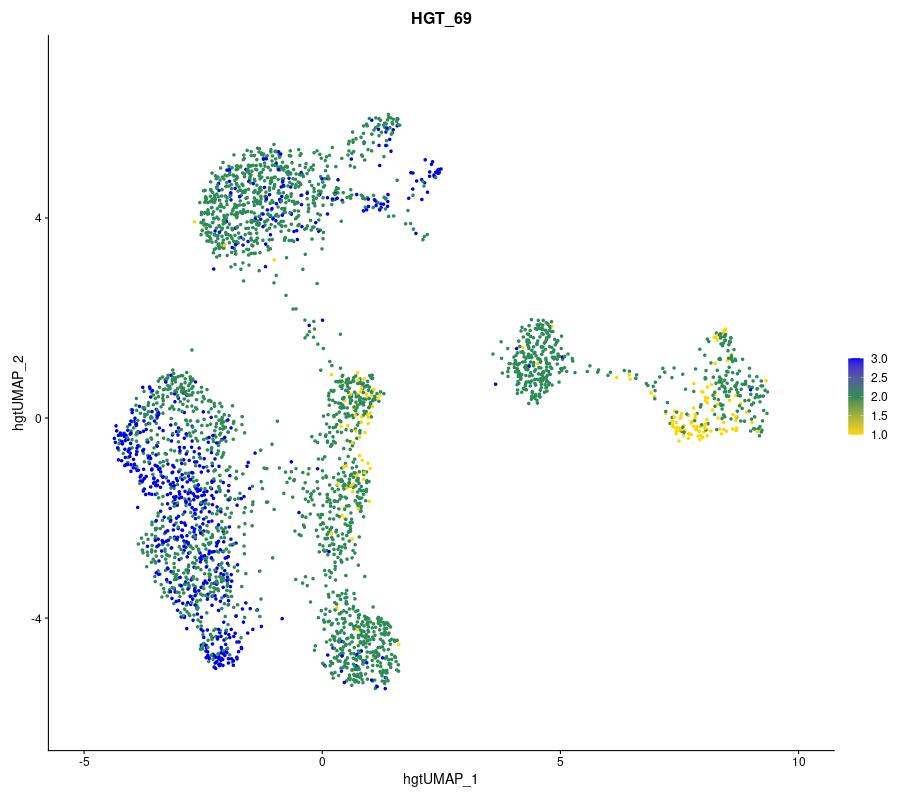

Supplement: Supplementary file 5 — Supplementary Data 3 [file 41467_2023_36559_MOESM5_ESM.zip › all128embedding/69 .jpg]

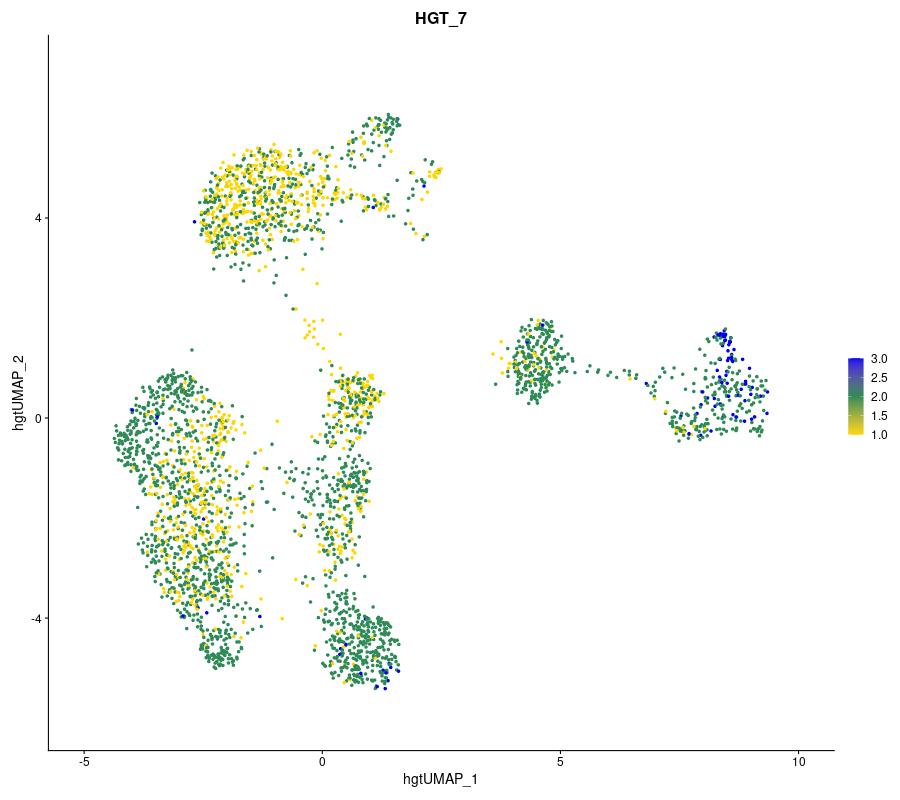

Supplement: Supplementary file 5 — Supplementary Data 3 [file 41467_2023_36559_MOESM5_ESM.zip › all128embedding/7 .jpg]

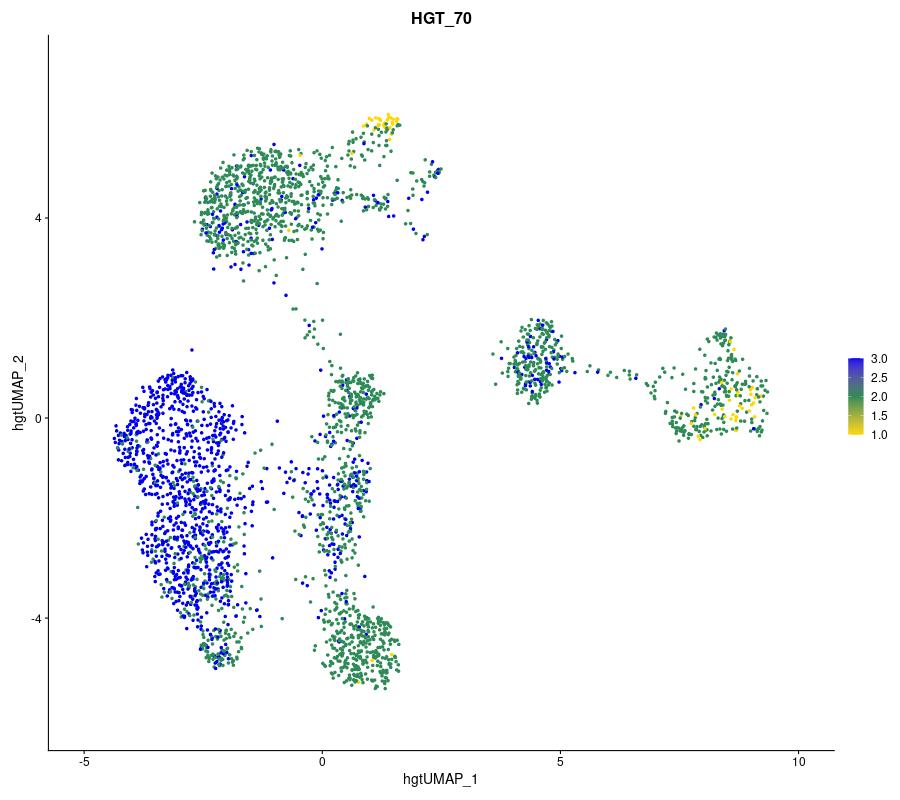

Supplement: Supplementary file 5 — Supplementary Data 3 [file 41467_2023_36559_MOESM5_ESM.zip › all128embedding/70 .jpg]

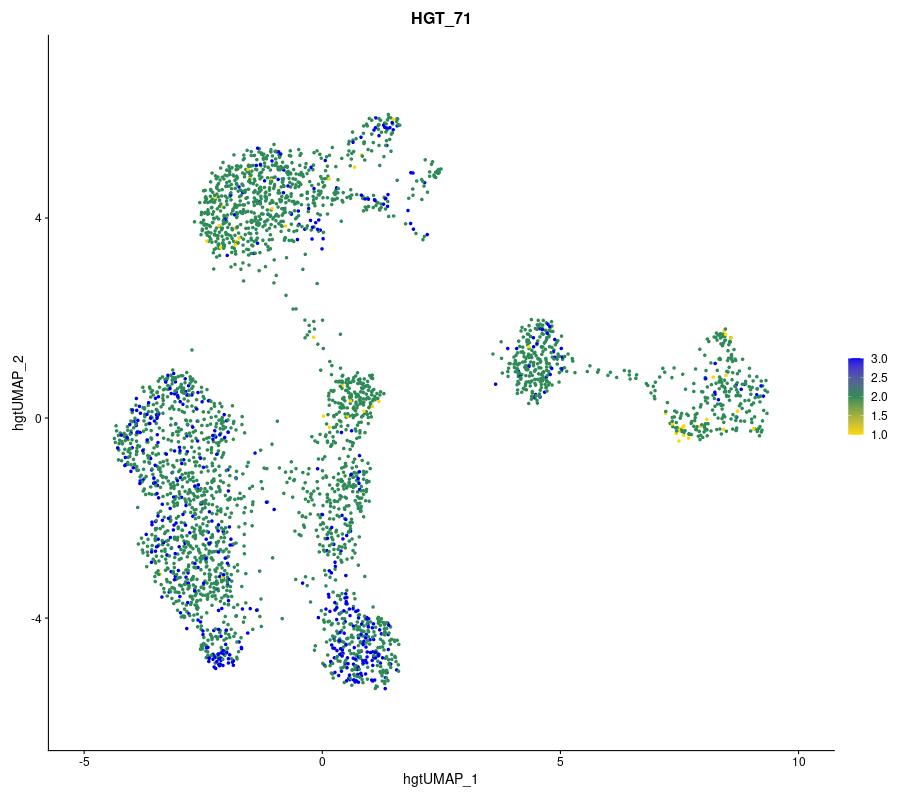

Supplement: Supplementary file 5 — Supplementary Data 3 [file 41467_2023_36559_MOESM5_ESM.zip › all128embedding/71 .jpg]

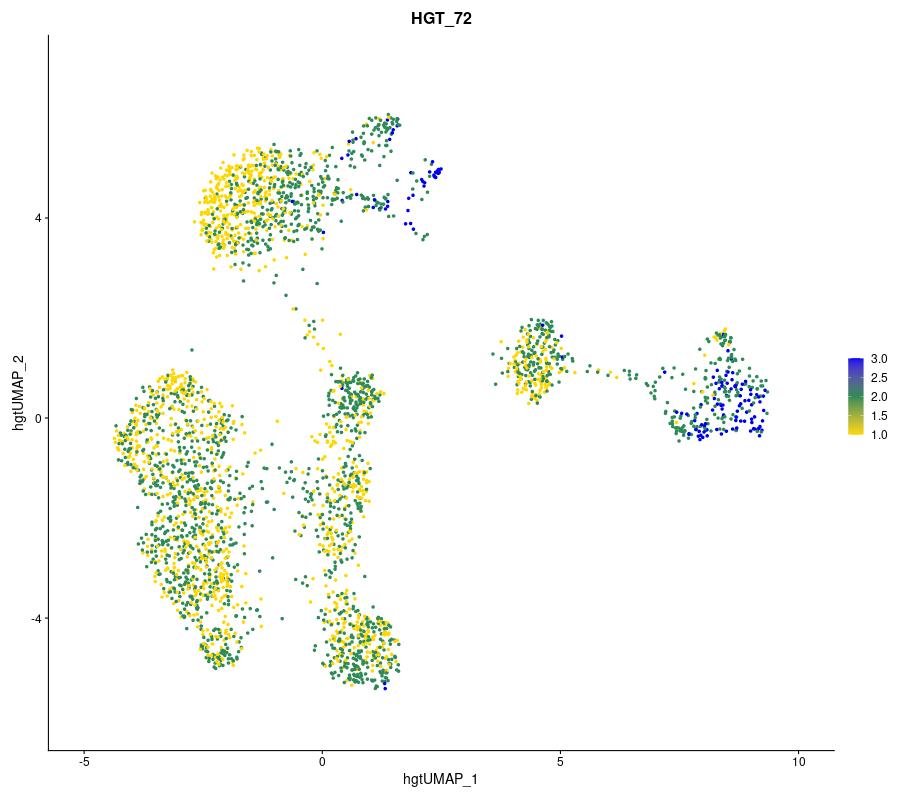

Supplement: Supplementary file 5 — Supplementary Data 3 [file 41467_2023_36559_MOESM5_ESM.zip › all128embedding/72 .jpg]

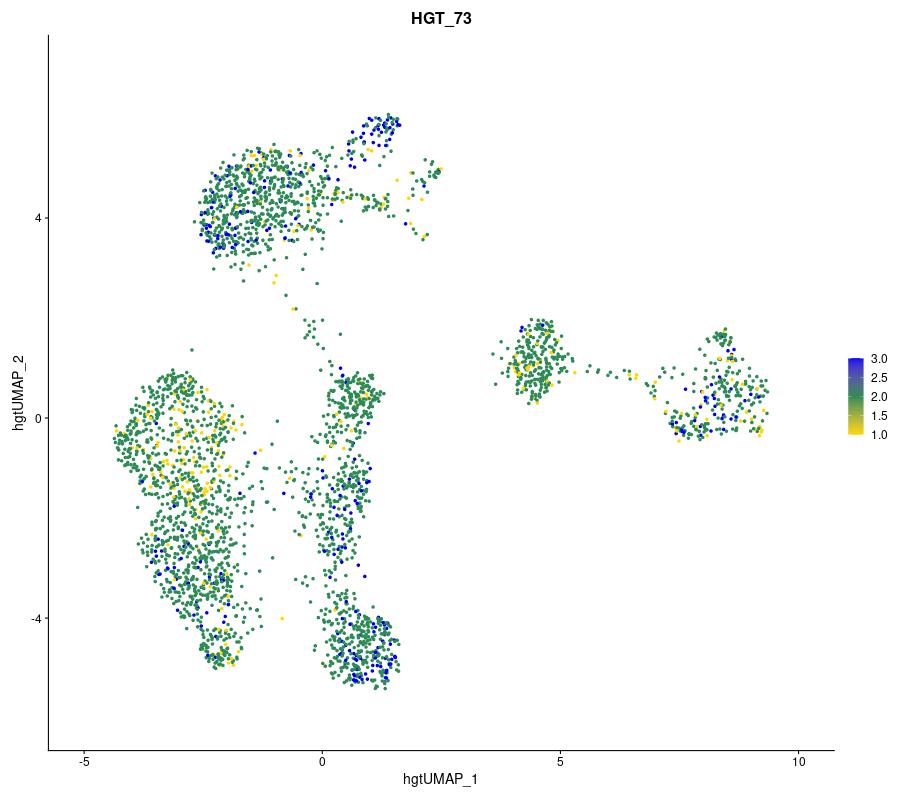

Supplement: Supplementary file 5 — Supplementary Data 3 [file 41467_2023_36559_MOESM5_ESM.zip › all128embedding/73 .jpg]
